# Effect of Cow-Calf Supplementation on Gene Expression, Processes, and Pathways Related to Adipogenesis and Lipogenesis in *Longissimus thoracis* Muscle of F1 Angus × Nellore Cattle at Weaning

**DOI:** 10.3390/metabo13020160

**Published:** 2023-01-21

**Authors:** Germán Darío Ramírez-Zamudio, Maria Júlia Generoso Ganga, Guilherme Luis Pereira, Ricardo Perecin Nociti, Marcos Roberto Chiaratti, Reinaldo Fernandes Cooke, Luis Artur Loyola Chardulo, Welder Angelo Baldassini, Otávio Rodrigues Machado-Neto, Rogério Abdallah Curi

**Affiliations:** 1 College of Animal Science and Food Engineering, São Paulo University (USP), Pirassununga 13635-900, SP, Brazil; 2 School of Agriculture and Veterinary Sciences (FCAV), São Paulo State University (UNESP), Jaboticabal 14884-900, SP, Brazil; 3 School of Veterinary Medicine and Animal Science (FMVZ), São Paulo State University (UNESP), Botucatu 18618-681, SP, Brazil; 4 Department of Genetics and Evolution, Federal University of São Carlos (UFSCAR), São Carlos 13565-905, SP, Brazil; 5 Department of Animal Science, Texas A&M University, College Station, TX 77843, USA

**Keywords:** *Bos indicus*, carcass, marbling, meat quality, nutrigenomics

## Abstract

The aim of this study was to identify differentially expressed genes, biological processes, and metabolic pathways related to adipogenesis and lipogenesis in calves receiving different diets during the cow-calf phase. Forty-eight uncastrated F1 Angus × Nellore males were randomly assigned to two treatments from thirty days of age to weaning: no creep feeding (G1) or creep feeding (G2). The creep feed offered contained ground corn (44.8%), soybean meal (40.4%), and mineral core (14.8%), with 22% crude protein and 65% total digestible nutrients in dry matter. After weaning, the animals were feedlot finished for 180 days and fed a single diet containing 12.6% forage and 87.4% corn-based concentrate. *Longissimus thoracis* muscle samples were collected by biopsy at weaning for transcriptome analysis and at slaughter for the measurement of intramuscular fat content (IMF) and marbling score (MS). Animals of G2 had 17.2% and 14.0% higher IMF and MS, respectively (*p* < 0.05). We identified 947 differentially expressed genes (log_2_ fold change 0.5, FDR 5%); of these, 504 were upregulated and 443 were downregulated in G2. Part of the genes upregulated in G2 were related to PPAR signaling (*PPARA*, *SLC27A1*, *FABP3*, and *DBI*), unsaturated fatty acid synthesis (*FADS1*, *FADS2*, *SCD*, and *SCD5*), and fatty acid metabolism (*FASN, FADS1*, *FADS2*, *SCD*, and *SCD5*). Regarding biological processes, the genes upregulated in G2 were related to cholesterol biosynthesis (*EBP*, *CYP51A1*, *DHCR24*, and *LSS*), unsaturated fatty acid biosynthesis (*FADS2*, *SCD*, *SCD5*, and *FADS1*), and insulin sensitivity (*INSIG1* and *LPIN2*). Cow-calf supplementation G2 positively affected energy metabolism and lipid biosynthesis, and thus favored the deposition of marbling fat during the postweaning period, which was shown here in an unprecedented way, by analyzing the transcriptome, genes, pathways, and enriched processes due to the use of creep feeding.

## 1. Introduction

Different strategies have been used in beef cattle to increase intramuscular fat (IMF) deposition. Early weaning and supplementation during the cow-calf phase are nutritional management practices that can induce different metabolic adaptations when compared to conventionally weaned animals [1]. The approach known as creep feeding consists of supplementation during the cow-calf (lactation) phase with grains or forage to obtain heavier individuals at weaning, to reduce the time to carcass finishing for slaughter, and to allow the dam to rest [2,3].

It is well-known that animals can respond to different environmental/nutritional factors by exhibiting phenotypic plasticity as a result of changes in gene expression patterns [4]. Thus, animal characteristics can be modified by nutritional modulations, which means that dietary exposures can have consequences for growth and health [5]. New evidence is constantly emerging that alterations in gene expression and in the phenotype of individuals, as a result of nutritional stimuli, may be due to epigenetic factors, including DNA methylation [6,7,8]. These modifications in gene expression can occur at specific loci or on a genomic scale [9,10,11,12,13].

In *Bos taurus*, it was identified that glucose is the best precursor for the synthesis of fatty acids in the intramuscular adipose tissue and the only carbonic compound with a higher concentration of its carbons in the intramuscular adipose tissue compared to the subcutaneous tissue, and in addition, lower glucose incorporation in post-pubertal animals was also noted [14]. Thus, the adequate intake of concentrate during the lactation period, especially the one rich in starch (grains), favors the proliferation and growth of adipocytes. This is due to the increase in glucose metabolism, which can lead to increased marbling without, however, increasing fat in the viscera, which contributes to overall meat quality and higher carcass yield [15]. Furthermore, Lopez et al. [16] proposed that higher levels of crude protein in the diet increase the digestion and intestinal absorption of starch, and promote the elevation of plasma insulin and glucose, which, consequently, increases the deposition of IMF, by favoring precursors of fatty acids.

Brazil, the world’s largest exporter of unprocessed beef for almost 20 years [17], has been witnessing the growing use of crossbred animals (*Bos taurus* × *Bos indicus*), especially Angus × Nellore. This strategy is used for the production of more tender meat with more IMF in an attempt to add value to the final product and to meet the demands of consumers who are willing to pay more for quality. IMF deposition in beef can positively influence sensory attributes such as flavor, juiciness, and tenderness [18]. In contrast, less IMF (marbling), which is observed in animals with a predominance of the *Bos indicus* genotype, compromises the sensory attributes of meat. This fact can be explained by the stimulation of fibrogenesis with declining intramuscular adipogenesis, with a consequent increase in connective tissue content [19]. However, the molecular mechanisms that control the growth of these tissues have not yet been completely elucidated.

Considering the lack of information about the changes in gene expression that occur in crossbred *Bos taurus* × *Bos indicus* calves supplemented during the cow-calf phase, this study aimed to evaluate the effect of creep feeding on gene expression, biological processes, and metabolic pathways related to adipogenesis and lipogenesis in *Longissimus thoracis* (LT) muscle biopsies collected at weaning from F1 Angus × Nellore cattle. Such data are of great economic importance for cattle production systems that aim to manipulate marbling and to obtain better quality meat to attend more demanding markets that pay for quality.

## 2. Materials and Methods

### 2.1. Animals

Forty-eight uncastrated (intact) F1 Angus × Nellore males born to the same Aberdeen Angus (*Bos taurus*) sire (half-siblings) were used.

The experiment (cow-calf phase) was conducted on a commercial farm located in the city of Anhembi, State of São Paulo, Brazil. The animals were submitted to two different treatments during most of the cow-calf phase (from 30 days of age to weaning—approximately 210 days), with 24 animals per treatment: group 1 (G1/T1)—no creep feeding (conventional weaning), and group 2 (G2/T2)—creep feeding. In the creep feeding system, the animals were kept with their mothers in the same paddock but had exclusive and free access to supplement corresponding to approximately 1% of body weight during all the cow-calf phase. The creep feed offered to animals of G2 (from 30 to 210 days of life) consisted of dry matter (22% crude protein and 65% total digestible nutrients) containing ground corn (44.8%), soybean meal (40.4%), and mineral core (14.8%).

After weaning (mean of 210 days), the animals of the 2 treatments were transferred to an experimental feedlot (Botucatu, São Paulo, Brazil), where they were housed in covered collective pens (three animals/pen with 10 m^2^ per animal) for approximately 180 days. The two groups received the same diet, containing 12.6% forage and 87.4% corn-based concentrate. The diet was formulated with the RLM 3.3 software (Ração de Lucro Máximo–Maximum Profit Ration, Piracicaba, São Paulo, Brazil) [20] using the NRC Tropicalizado ESALQ system and consisted of corn, soybean meal, Tifton hay, sugarcane bagasse, urea, and vitamin–mineral supplement (Table S1). The diet was offered ad libitum, twice a day at 8 am and 4 pm.

The animals were weighed after a 16 h fast at the beginning of the cow-calf phase (initial weight—BWi), at the end of weaning (weaning weight—WW), and at the end of the feedlot period (final weight—BWf). The average daily weight gain 1 (ADG1: beginning of cow-calf phase to weaning) was calculated from BWi and WW, and ADG2 (weaning to the end of the feedlot period) from BWf and WW.

### 2.2. Collection of Muscle Tissue at Weaning and Slaughter

At weaning (210 days), fragments of the LT muscle were collected by biopsy from 12 animals randomly selected from each treatment (n = 24). For biopsy, the lumbar region was shaved, and a local anesthetic was subcutaneously administered. The biopsies were performed at the height of the 13th rib. After cleaning the biopsy site, a 1 cm incision was made with a scalpel and a sterile Bergstrom biopsy needle was used to obtain 1 g of muscle tissue. The sample was immediately transferred to liquid nitrogen and stored in an ultra-freezer at −80 °C.

After the feedlot period, the 48 animals were slaughtered using the cerebral concussion technique and sectioning of the jugular vein. The carcasses were identified, washed, and divided into two halves. The half-carcasses were weighed individually to obtain the hot carcass weight (HCW) and kept in a cold room for approximately 24 h at 1 °C.

After cooling, the carcasses were removed from the cold room and weighed. After weighing, the LT muscle of the left half-carcass was separated and backfat thickness (BFT) and rib eye area (REA) were evaluated between the 12th and 13th ribs before deboning. Beef samples (sirloin steaks) were collected between the 12th and 13th ribs (cranial direction) of the left half-carcass and used for the laboratory analysis of physicochemical quality attributes.

### 2.3. Analysis of Meat Quality

The following physicochemical meat quality attributes were analyzed in the 48 slaughtered animals (n = 24/treatment): marbling score (MS), total lipids/intramuscular fat percentage (IMF), and Warner–Blatzler shear force (WBSF). The MS was determined by a single, previously trained evaluator following the Brazil Beef Quality reference standards (https://www.brazilbeefquality.com/) (accessed on 12 June 2021). These standards are numbered from 1 to 11 and provide a point scale ranging from 100 to 1100 (adapted from AUS-MEAT [21]), where the closer to 1100, the more marbled the meat, and the closer to 100, the less marbled the meat. The IMF was determined by infrared spectroscopy using a FoodScan^TM^ (Foss NIRSystems, Laurel, MD, USA). The procedure standardized by Shackelford et al. [22] was adopted to measure WBSF at 7 and 14 days of aging (WBSF7 and WBSF14).

### 2.4. Statistical Analysis of Weight, Weight Gain, Carcass, and Meat Data

The weight and weight gain data and carcass and meat traits (BWi, WW, ADG1, BWf, ADG2, HCW, REA, BFT, MS, IMF, WBSF7, and WBSF14) of the 48 samples (n = 24/group) were analyzed regarding the presence of outliers, homogeneity of variance, and normality of residuals. The data were expressed as means and their respective standard errors. Transformations of the variables to provide an approximation of the normal distribution were not necessary. The means of the groups were compared by the *t*-test to detect significant differences (*p* < 0.05). These analyses were performed using the R software v.4.2.1 (Vienna, Austria) [23].

### 2.5. Analysis of Differential Gene Expression

#### 2.5.1. RNA Extraction and Sequencing

After the extraction of total RNA, 24 genomic libraries were prepared, which consisted of 12 samples from each group/treatment (G1 and G2) collected at weaning. Total RNA was extracted individually from 100 mg of LT muscle using TRIzol^®^ (Life Technologies, Carlsbad, CA, USA), according to the manufacturer’s instructions, and its quality was analyzed in a Bioanalyzer 2100^®^ (Agilent, Santa Clara, CA, USA). A minimum RNA integrity number (RIN) ≥ 7 was adopted to ensure adequate quality of the total RNA. RNAs with a poly-A tail, mainly the mRNA, were purified from total RNA using oligo-dT beads. It is well-known that many eukaryotic non-coding RNAs can have a poly-A tail in intermediary or in mature forms [24] and, in this way, eventually be captured by oligo-dT bead columns.

The cDNA libraries of each sample were prepared and multiplexed using the TruSeq RNA Sample Preparation kit (Illumina, San Diego, CA, USA) from 2 µg of total RNA, according to the TruSeq RNA Sample Preparation kit v2 guide (Illumina). The Bioanalyzer 2100^®^ (Agilent) was used to estimate the average size of the libraries and quantitative PCR (RT-qPCR) using the KAPA Library Quantification kit (KAPA Biosystems, Wilmington, MA, USA) to quantify them. Clustering and sequencing were performed in one lane using the HiSeq2500 kit v4 2 × 100 bp (Illumina) to produce 100 bp paired-end (PE) reads. The HiSeq 2500^®^ sequencer (Illumina) was used to reach a minimum coverage of 16 million reads per sample.

#### 2.5.2. Mapping of Sequences to the Reference Genome and Identification of Differentially Expressed Genes

The sequence data generated by the HiSeq System Illumina platform were converted to FastQ format and separated by library (multiplexed data) using the Casava 1.8.2 software (Illumina). The FastQC v.0.11.9 software [25] was used to analyze the quality of raw reads. Adapter sequences and low-quality sequences were removed using Fastp v.0.23.1 [26]. After this step, the quality of the reads was reassessed by combined visualization of all FastQC outputs using the MultiQC v.1.13 program [27] to confirm improvements in quality. Next, the reads were mapped to the bovine reference genome (*Bos taurus*—ARS-UCD1.3), available at: http://www.ensembl.org/Bos_taurus/Info/Index/ (accessed on 19 August 2021), using the STAR v.2.7.20 program [28]. Mapping was performed independently for each sample. For each library, a file with the .bam extension was generated, which contained the alignment of the fragments to the reference genome. Mapped reads were counted using featurecounts v.2.0.3 [29] and only PE reads mapped to a single position of the genome (uniquely mapped PE reads) and to known chromosomes were used for differential gene expression analysis.

Differential gene expression was compared between the different groups/treatments at weaning (i.e., G1 vs. G2). First, graphical principal component analysis (PCA) of the read counts normalized to counts per million (CPM) was performed using the factoextra package [30] of the R software [23] to divide the samples based on gene expression patterns, examining the level of similarity/dissimilarity between groups. Differentially expressed genes (DEGs) were identified using the method implemented in edgeR v.3.40.0 [31] of the R software [23] for parameter estimation by the maximum likelihood method. Generalized linear models were used, assuming a negative binomial distribution of the count data. For this purpose, size factors were computed by the trimmed mean of M-values (TMM) for each pair of samples and overdispersion parameters were estimated for each gene by the Cox–Reid method [32]. The expression of each gene was calculated as the mean expression in all samples of each group and is reported as the mean of the logarithmic function of CPM. The fold change was calculated as the logarithmic function of the ratio between the CPM of G2 and G1 for each gene. The *p*-value associated with the difference in gene expression between groups was obtained by the likelihood ratio test. The Benjamini–Hochberg procedure [33] was used to control the false discovery rate (FDR). A log_2_ fold change of 0.5 and significance adjusted to FDR < 0.05 were adopted to identify DEGs. The expression profiles of 13 constitutive genes were analyzed to evaluate the quality of sequencing. These genes were: *actin beta—ACTB*; *beta-2-microglobulin—B2M*; *glyceraldehyde-3-phosphate dehydrogenase—GAPDH*; *glucuronidase beta—GUSB*; *hydroxymethylbilane synthase—HMBS*; *hypoxanthine phosphoribosyltransferase 1—HPRT1*; *phosphoglycerate kinase 1—PGK1*; *peptidylprolyl isomerase A—PPIA*; *ribosomal protein L13a—RPL13A*; *ribosomal protein lateral stalk subunit P0—RPLP0*; *succinate dehydrogenase complex flavoprotein subunit A—SDHA*; *TATA-box binding protein—TBP*; *transferrin receptor—TFRC*.

#### 2.5.3. Functional Analysis of Differentially Expressed Genes

To understand the functional role of the genes identified as DEGs between groups at weaning, lists of up- and down-regulated genes were used in over-representation analysis (ORA) of gene ontology terms (GO terms: biological processes) and metabolic pathways (Kyoto Encyclopedia of Genes and Genomes (KEGG) database) using the online Database for Annotation, Visualization, and Integrated Discovery (DAVID v.6.8; https://david.ncifcrf.gov/home.jsp) (accessed on 12 October 2021) [34]. Biological processes and metabolic pathways were defined as enriched in the presence of at least three genes in each pathway or process and a *p*-value < 0.05.

The functional annotation clustering function of DAVID and the ClueGO package [35] of Cytoscape v.3.9.1 were used to identify the relationships between enriched processes and pathways. Due to their relationships with each other and their potential relationships with intramuscular fat content in cattle, processes and pathways were highlighted. To identify/visualize the participation of DEGs in the different enriched and highlighted biological processes and pathways, a HeatMap was generated using the ComplexHeatMap package [36] of the R software. The ClueGO and CluePedia packages [37] of Cytoscape were used to generate networks of shared DEGs between KEGG pathways and biological processes (FDR < 5%).

The online STRING (Search Tool for the Retrieval of Interacting Genes/Proteins) database (version 11.5), which collects and integrates information on functional interactions between genes/proteins for a large number of organisms [38], was used to reveal and visualize functional interactions between the DEGs of biological processes and KEGG pathways identified as enriched by DAVID and highlighted due to their relationships visualized in ClueGO and potential relationships with intramuscular adipogenesis and lipogenesis. Protein–protein interactions (PPIs) with a confidence score > 0.4 (a commonly used threshold) and an FDR < 0.05 were considered and are shown in the graph. The Markov cluster algorithm (MCL), an unsupervised clustering algorithm for graphs based on simulation of stochastic flow, was used for the clustering of genes (nodes) using the default inflation parameter 3 of STRING.

The *Bos taurus* database was used for the analyses that employed the DAVID, ClueGO/CluePedia, and STRING programs/packages.

## 3. Results

### 3.1. Pre- and Post-Weaning Performance, Carcass and Meat Quality

Table 1 shows the body weight of the animals at the different time points, weight gain, and carcass-related information. Significant differences (*p* < 0.05) between groups/treatments were observed for WW and ADG1. Regarding WW, animals of G1 (no supplementation) were lighter and those of G2 (creep feeding) were heavier. The same situation was observed for ADG1, i.e., G2 > G1. Animals of G1 and G2 did not differ in terms of BWf, and likewise for ADG2 during the feedlot period. No significant differences in BWi or HCW were observed between groups.

Table 2 shows the quality variables, which were measured or estimated objectively or subjectively in the carcasses or meat samples of animals by laboratory analysis. No significant differences (*p* > 0.05) between groups were observed for REA, WBSF7, or WBSF14. On the other hand, there were significant differences (*p* < 0.05) in BFT, IMF, and MS. Animals of G2 (creep feeding) exhibited a higher mean BFT than G1 animals (no supplementation). Regarding IMF, an objective measure for the assessment of marbling, G2 animals had a higher fat content/percentage than G1 animals. Visual differences in relation to the fat content between LT muscle samples collected from animals of G1 and G2 are shown in Figure S1. The MI, a subjective measure of marbling, was higher in G2 animals compared to G1.

### 3.2. Analysis of Differential Gene Expression

#### 3.2.1. Concentration and Integrity of Total RNA

The mean total RNA concentration extracted from the 24 samples was 260.27 ng/µL. The 260/280 nm (nucleic acid/protein) and 260/230 nm (nucleic acid/extraction contaminants) ratios were approximately 1.9, a value considered to be adequate. The mean contamination with genomic DNA was 1.04% (range: 0.73% to 1.11%). The mean RIN was 7.6 (range: 7.0 to 8.0). Thus, the samples were intact (all RINs > 7) and free of contaminants.

#### 3.2.2. RNA Sequencing and Mapping of Reads to the Reference Genome

A total of 241.2 million PE reads (2 × 100 bp) were obtained; of these, 230.5 million were uniquely mapped PE reads. The coverage achieved by sequencing was 37X (coverage for all transcripts of all samples). An average of 9.6 million uniquely mapped PE reads were obtained per sample, corresponding to 95.56% of all PE reads generated.

The reads were mapped to 27,607 genes (protein coding and non-coding). However, considering a count of uniquely mapped PE reads ≥ 3 in at least 12 samples, the total number of expressed genes, and thus used in the differential gene expression analyzed, was 16,604. The number of genes detected by functional category after application of a filter/threshold that excludes genes with a low count is presented in Figure S2. Following protein-coding genes (15,672), the largest number of sequenced genes were those encoding transcription factors (656) and non-coding genes such as long non-coding RNA (lncRNA), short nuclear RNA (snRNA), and micro-RNA (miRNA). The latter are related to the control of gene expression and processing of messenger RNA.

The number and percentage of transcripts/fragments aligned to the bovine reference genome identified in samples collected from non-creep-fed (G1) and creep-fed (G2) animals, which showed significant differences between means for marbling measurements—IMF and MS (G1 < G2, *p* < 0.05), are shown in Table 3 and Table 4, respectively.

The boxplots of the read counts normalized by the size factor showed that the distribution of quartiles was consistent between the samples of the two groups, indicating good quality of the sequencing data (Figure S3).

**Figure 1:**
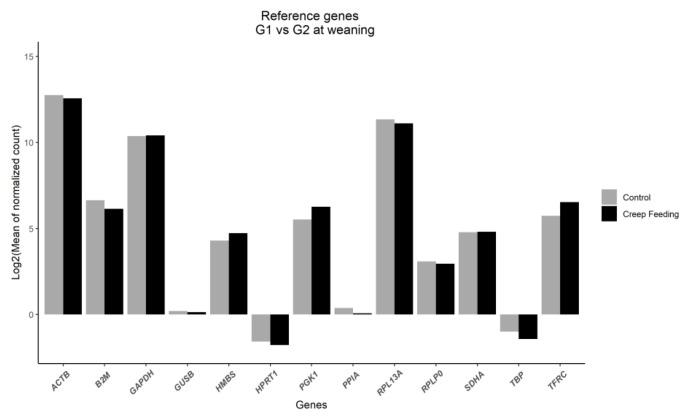
Expression profile of reference genes in the control group (G1, no creep feeding) and the group submitted to creep feeding (G2).

In relation to the expression profile of the constitutive genes, the expression was similar between the experimental groups (Figure 1).

**Figure 2:**
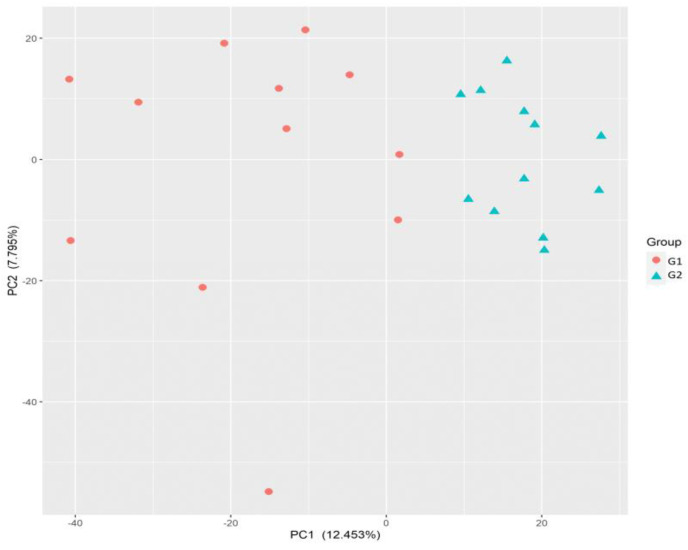
Principal component (PC) analysis performed based on normalized count data of gene expression for samples collected at weaning from G1 (control, no creep feeding) and G2 (creep feeding).

PCA showed that the first two principal components explained more than 20% of the variation among samples (Figure 2). In addition, the formation of clearly distinct groups of samples was observed at weaning, indicating an evident difference in the expression of genes between treatments. This fact illustrates the effect of cow-calf supplementation (creep feeding) on gene expression at weaning.

#### 3.2.3. Identification of Differentially Expressed Genes

Fragments of LT muscle obtained by biopsy were used to identify differences in global gene expression between G1 and G2 at weaning. A total of 947 DEGs were identified (log_2_ fold change < −0.5 or >0.5, FDR < 5%) between groups at weaning; of these, 443 were downregulated and 504 were upregulated in G2 (creep feeding).

**Figure 3:**
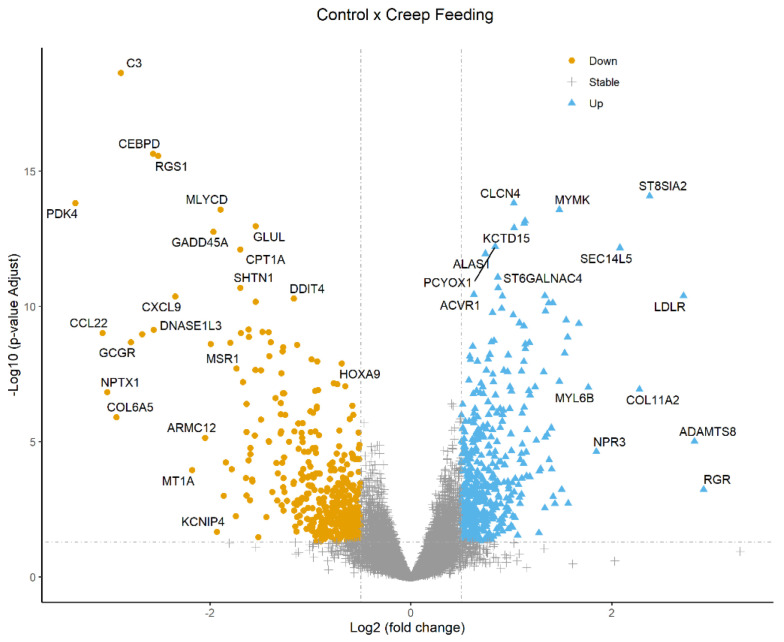
Volcano plot of log_2_ fold change (*x*-axis) versus -log_10_ *p*-value (FDR, *y*-axis) indicating differences in DEGs identified by the edgeR method (downregulated: log_2_ fold change < −0.5 and FDR < 0.05; upregulated: log_2_ fold change > 0.5 and FDR < 0.05) between G1 (control) × G2 (creep feeding) at weaning.

Figure 3 shows a Volcano plot that illustrates genes differentially expressed in G1 × G2 at weaning. The top 30 DEGs for comparison, with an adjusted *p*-value (FDR) < 0.05, are shown in Table 5. The complete list of the differentially expressed genes, with log_2_ fold change, *p*-value, and adjusted *p*-value of down- and up-regulated genes for comparison between groups at weaning, is provided in Table S2.

#### 3.2.4. Functional Enrichment Analysis of Differentially Expressed Genes

Regarding the genes upregulated in G2 at weaning (n = 504), ORA identified 22 enriched metabolic KEGG pathways (*p* < 0.05) (Table S3). Among the pathways identified, five are highlighted due to their relationships with each other (identified by DAVID and ClueGO) and their potential relationships with adipose cell proliferation/adipogenesis and synthesis and degradation of intramuscular fat (lipolysis/lipogenesis) in cattle: PPAR signaling pathway (bta03320), steroid biosynthesis (bta00100), biosynthesis of unsaturated fatty acids (bta01040), apelin signaling pathway (bta04371), and fatty acid metabolism (bta01212). The upregulated DEGs in the enriched pathways and additional information are provided in Table 6. Regarding GO terms, 34 enriched biological processes were identified (*p* < 0.05) (Table S4). Four of these processes are highlighted due to their relationships with each other and potential relationships with intramuscular fat content in cattle: cholesterol biosynthetic process (GO:0006695), unsaturated fatty acid biosynthetic process (GO:0006636), sterol biosynthetic process (GO:0016126), and cellular response to insulin stimulus (GO:0032869). Table 7 shows the upregulated DEGs related to these processes.

Considering the genes downregulated in G2 at weaning (n = 443), 46 enriched pathways were identified (*p* < 0.05) (Table S5), and 3 of these pathways are highlighted due to the aforementioned relationships: AMPK signaling pathway (bta04152), glucagon signaling pathway (bta04922), and PPAR signaling pathway (bta03320). The downregulated DEGs participating in these pathways and additional information are provided in Table 6. Regarding GO terms, 52 enriched biological processes were identified (*p* < 0.05) (Table S6), and 3 are highlighted due to the aforementioned relationships: positive regulation of lipid storage (GO:0010884), fatty acid metabolic process (GO:0006631), and negative regulation of glycolytic process (GO:0045820). Table 7 shows the downregulated DEGs related to these processes.

**Figure 4:**
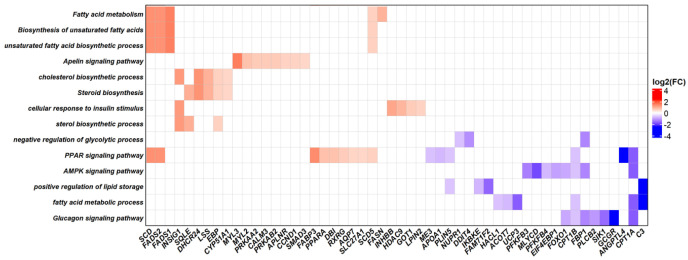
Relationship between up- and down-regulated DEGs identified in G2 at weaning (*x*-axis) and enriched biological processes and pathways, which are highlighted due to their relationships with each other and possible relationships with intramuscular fat content in cattle (*y*-axis). The color scale indicating the log_2_ fold change is given on the right side of the figure.

Figure 4 shows the HeatMap that illustrates the relationship between each of the 52 up- and down-regulated DEGs identified in G2 at weaning and the 14 biological processes and metabolic pathways enriched in ORA, which are highlighted due to their relationships with each other and potential relationships with intramuscular fat content in cattle. This approach permitted to observe the participation of DEGs in one or more processes or pathways, as well as the magnitude of differences in gene expression between treatments.

**Figure 5:**
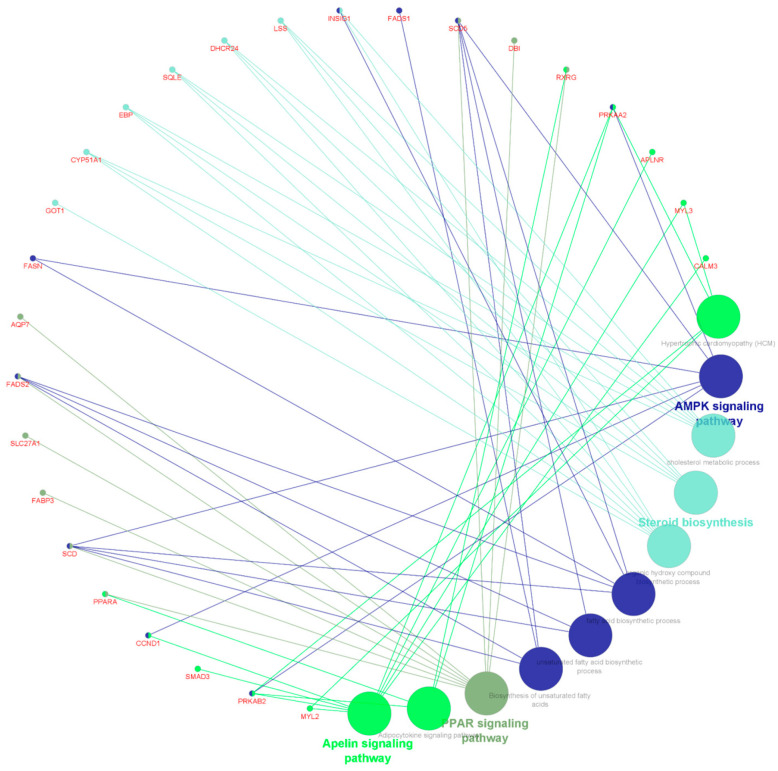
Circular network of shared upregulated DEGs in G2 at weaning between biological processes and KEGG pathways, which were highlighted in ORA and due to their potential relationship with intramuscular fat in cattle.

**Figure 6:**
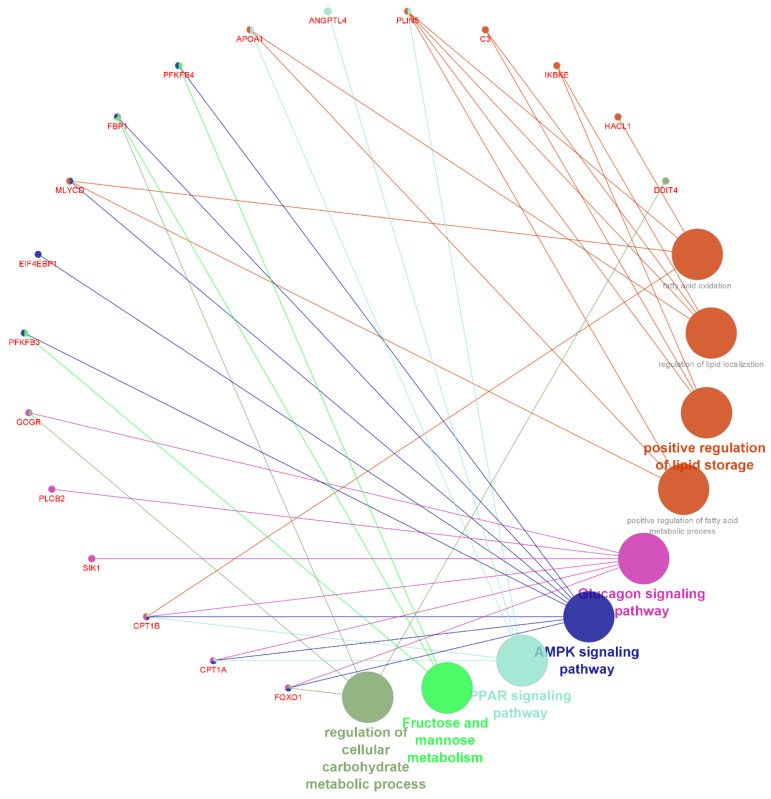
Circular network of shared downregulated DEGs in G2 at weaning between biological processes and KEGG pathways, which were highlighted in ORA and due to their potential relationship with intramuscular fat in cattle.

Networks of shared up- and down-regulated DEGs in G2 at weaning between biological processes (GO_BP) and KEGG pathways, which were highlighted in ORA and due to the potential relationship with intramuscular fat/marbling in *Bos taurus,* are shown in Figure 5 and Figure 6, respectively.

**Figure 7:**
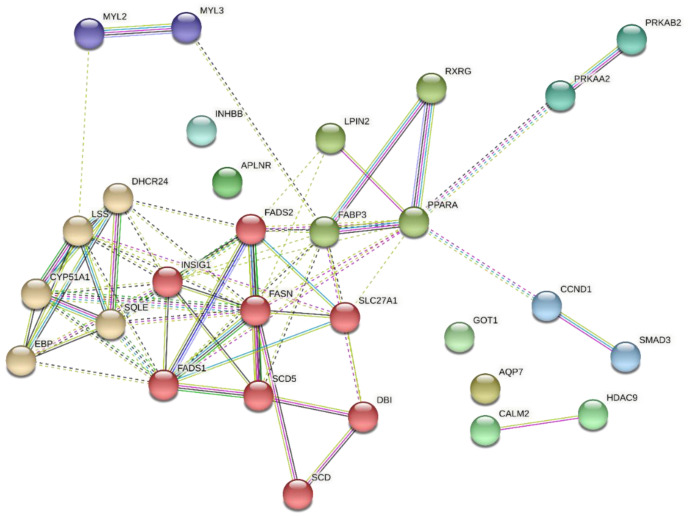
Protein–protein interaction network of DEGs upregulated in G2 that participate in the biological processes and KEGG pathways identified as enriched and highlighted in the G1 × G2 comparison at weaning. Type of interaction (edge color) between nodes (DEGs): light green—text mining; dark green—neighborhood; light blue—curated databases; dark blue—co-occurrence; pink—experiments; purple—protein homology; black—co-expression. A larger number of edges indicate stronger evidence/greater strength of the interaction between two nodes. The nodes that make up the clusters formed (joined by dotted edges) are illustrated with different colors.

Figure 7 shows the network of PPI of 29 DEGs upregulated in G2 that participate in the biological processes (GO_BP) and metabolic pathways (KEGG) identified as enriched and highlighted in the G1 × G2 comparison at weaning due to their relationships with each other and potential relationships with intramuscular adipogenesis and lipogenesis. Sixty-nine edges (significant interactions) and six clusters consisting of eight to two genes were identified. The red and yellow clusters were the largest, with eight and five genes, respectively. Three clusters (dark green, light blue, and dark blue) contained only two genes. The mean clustering coefficient was 0.512. Six DEGs were disconnected from the network.

**Figure 8:**
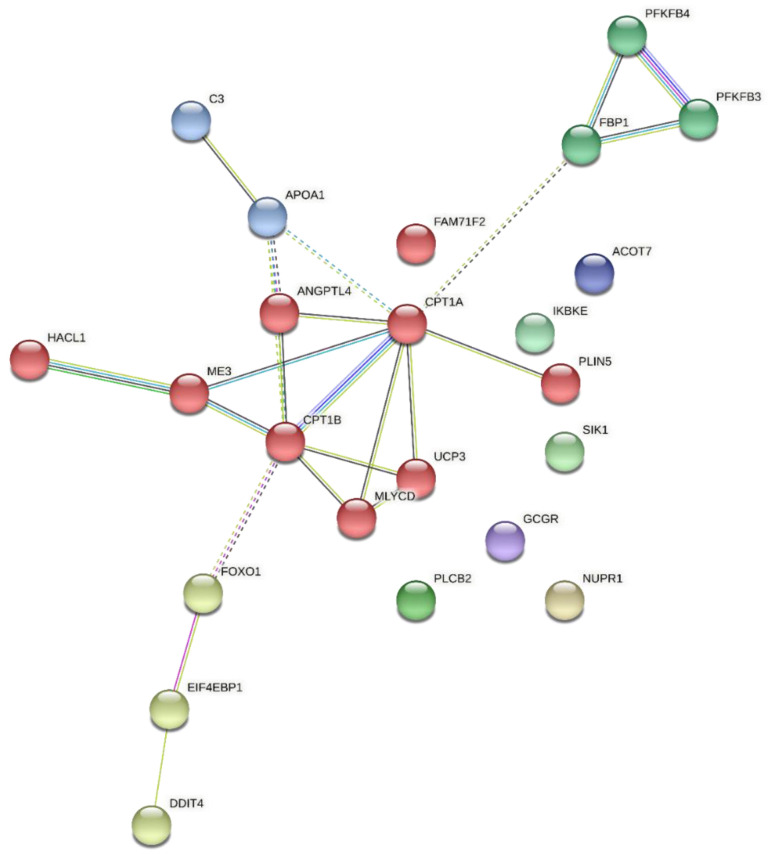
Protein–protein interaction network of DEGs downregulated in G2 that participate in the biological processes and KEGG pathways identified as enriched and highlighted in the G1 × G2 comparison at weaning. Type of interaction (edge color) between nodes (DEGs): light green—text mining; dark green—neighborhood; light blue—curated databases; dark blue—co-occurrence; pink—experiments; purple—protein homology; black—co-expression. A larger number of edges indicate stronger evidence/greater strength of the interaction between two nodes. The nodes that make up the clusters formed (joined by dotted edges) are illustrated with different colors.

Figure 8 shows the network of PPI of 23 DEGs downregulated in G2 that participate in the biological processes (GO_BP) and metabolic pathways (KEGG) identified as enriched and highlighted in the G1 × G2 comparison at weaning due to their relationships with each other and potential relationships with intramuscular adipogenesis and lipogenesis. Twenty-three edges (significant interactions) and four clusters consisting of eight to two genes were identified. The red, yellow, and dark green clusters were the largest, with eight, three, and three genes, respectively. One of the clusters (dark blue) contained only two genes. The mean clustering coefficient was 0.467. Seven DEGs were disconnected from the network.

## 4. Discussion

### 4.1. Pre- and Post-Weaning Performance, Carcass and Meat Quality

In tropical breeding systems, milk production of Nellore cows, which form the basis of the Brazilian cattle herd, no longer meets the calf’s requirements for expression of its growth potential after the third month of lactation [39]. In F1 Angus × Nellore cattle, this interval is believed to be even shorter. Thus, obtaining the nutrients necessary to meet the growth requirements during lactation increasingly depends on the forage consumed by the animal. An important fact of the pasture-based systems in Brazil is that, in addition to the decline in the lactation curve of cows, the mass and nutritive value of pastures decrease due to seasonality, while the calf’s nutritional requirements increase with the progression of growth [40]. Within this context, creep feeding is a strategy used to compensate the nutrient deficits of milk and forage, in addition to stimulating greater muscle development in the animal, which results in a shorter time to slaughter and improvement in carcass quality [41]. However, due to issues related to the offer of supplement, high supplies can affect fiber digestion from pasture, with negative consequences for feed efficiency [42,43] and calf performance [44]. This fact was not observed in the present study; on the contrary, calves that received 1% of their body weight as a supplement exhibited an additional daily weight gain of approximately 100 g and an additional total gain of 14.6 kg at weaning when compared to non-supplemented animals. However, in a meta-analysis conducted by Carvalho et al. [38], the additional daily weight gain was about 200 g and the total gain at weaning was 30 kg in male calves supplemented between 3 and 8 months of age when compared to non-supplemented animals. In the present study, supplementation during the lactation period did not exert any long-term effect on the performance of animals during the finishing period (BWf, ADG2, and HCW; Table 1). This finding suggests that the lack of difference in the efficiency of animals during the postweaning period may be related to gain composition [45], i.e., an early increase in the fat deposition rate may reduce the rate of lean tissue deposition [46]; consequently, weight gain in subsequent phases may be moderate. Similarly, calves supplemented during the lactation period showed a higher degree of carcass finishing (BFT) and greater fat deposition in beef, while no differences in weight gain were observed (Table 2).

### 4.2. Differentially Expressed Genes and Alterations in Biological Processes and Metabolic Pathways

Intramuscular fat is a desirable characteristic in some niche markets because of its positive effect on flavor, juiciness, and greater consumer perception of meat tenderness [47]. Although this quality trait is attributed to intense cell proliferation during the fetal period, there is a period of postnatal life within the first 250 days, known as the “marbling window”, during which a high-grain diet can specifically lead to the recruitment of intramuscular adipocytes (adipocyte hyperplasia) that provide sites for fat deposition during finishing [48].

Studies indicate that the PPAR signaling pathway is important for the regulation of cell differentiation, energy balance, and lipid metabolism [49,50]. During lipogenesis, activation of this pathway upregulates the expression of *FABP*, *FASN*, and *SCD* [51,52]. The last two genes are important for de novo fatty acid synthesis [53] and were upregulated in creep-fed animals in the present study. Furthermore, Ward et al. [54] reported that, in addition to the upregulation of *FASN*, an increase in the level of marbling in beef is associated with the expression of Δ-6 and Δ-5 desaturases, enzymes that are regulated by *PPARA* [55]. This confirms that *PPARA* also acts on lipogenesis in skeletal muscle [56]. The Δ-5 and Δ-6 desaturases encoded by the *FADS1* and *FADS2* genes, respectively, belong to the fatty acid desaturase family, key proteins in the first desaturation reaction for endogenous formation of polyunsaturated fatty acids from dietary essential fatty acids (linoleic and linolenic) [57]. Linoleic and linolenic acids are unsaturated fatty acids found in oilseeds such as soybeans [58]. Although these fatty acids may undergo high ruminal biohydrogenation, part of them may escape into the duodenum and be absorbed [59]. In the present study, consumption of approximately 375 g of soybean meal per day is estimated in animals supplemented with 1% of body weight. Thus, part of the unsaturated fatty acids consumed from the supplement may have reached the skeletal muscle, with consequent upregulation of *FADS1* and *FADS2*.

Although calf supplementation during the lactation period had positive effects on the PPAR signaling pathway in lipid metabolism, downregulation of this pathway can downregulate the *APOA1* and *ANGPTL4* genes [60]. The *APOA1* gene encodes the structural and functional protein component of high-density lipoprotein (HDL), which promotes the reverse flow of cholesterol from tissues to excretion in the liver [61]. In the present study, downregulation of *APOA1* in supplemented calves may be a long-term effect of unsaturated fatty acid intake from soybean meal. Some studies have shown that the consumption of unsaturated fatty acids promotes a decrease in the expression of *APOA1* [62,63]. On the other hand, angiopoietin-like 4 encoded by the *ANGPTL4* gene mediates the inactivation of lipoprotein lipase involved in lipid metabolism [64], thereby inhibiting the uptake of triglycerides in adipocytes [65]. Downregulation of *APOA1* and *ANGPTL4* expression in supplemented calves suggests the accumulation of fatty acids, probably controlled by lipoprotein lipase that reduces the efflux of triglycerides from adipose tissue.

Calf supplementation at 1% of body weight during the lactation period positively influenced the lipogenic program by regulating genes related to the biosynthesis (*FASN*, *SCD*, *SCD5*, *FADS1*, and *FADS2*) and uptake of fatty acids (*FABP3*), while reducing the expression of transcription factors related to β-oxidation (*CPT1A, CPT1B*, and *UCP3*). A similar result was reported in a study on yaks, herbivores of the genus *Bos*, in which supplementation during the growth period stimulated de novo synthesis of fatty acids by upregulating *SREBF1*, *ACACA*, *FASN,* and *SCD1*, as well as the transcription factor *H-FABP* (*FABP3*), and downregulating *CPT1* [66]. Studies with rodents concluded that increased intracellular glucose availability inhibits CPT1 due to elevated malonyl-CoA concentrations which, in turn, reduce fatty acid oxidation [67,68]. In the present study, calves fed via creep feeding ingested high amounts of starch (>500 g/day), which can be transformed into glucose. Therefore, a high amount of glucose reaching the muscle tissue of these animals may have reduced the expression of genes related to fatty acid oxidation (*CPT1A*, *CPT1B*, and *UCP3*).

We also observed the regulation of genes that participate in the AMPK pathway of energy metabolism. However, the upregulation of the *PRKAA2* and *PRKAB2* genes, which encode the catalytic subunits necessary for AMPK activation during fatty acid oxidation [69], observed in the present study in supplemented calves was contradictory. A comparison between Angus and crossbred Angus × Simmental cattle, with the former exhibiting greater marbling potential, showed lower expression of these AMPK regulatory genes [70]. However, a study using cardiac muscle cells of mice indicated that AMPK acts as a downstream signaling molecule of apelin [71]. The latter plays an important role in energy metabolism by improving insulin sensitivity [72]. Another important enzyme in energy metabolism and fatty acid metabolism regulated by AMPK is malonyl-CoA decarboxylase [73]. This mitochondrial enzyme encoded by the *MLYCD* gene, which is responsible for increasing fatty acid oxidation by converting malonyl-CoA to acetyl-CoA [74], was downregulated in supplemented calves (Table 5). Studies on humans [74] and mice [75] have shown that silencing of this gene in muscle tissue increases the concentration of malonyl-CoA, thus increasing the utilization of glucose while reducing fatty acid oxidation. This fact may explain the downregulation of genes related to β-oxidation (*CPT1A*, *CPT1B*, and *UCP3*) in supplemented calves and may represent a protection mechanism against the development of diet-induced insulin resistance [76].

Higher expression of the complement 3 (*C3*) gene has been demonstrated in individuals with insulin resistance or hyperinsulinemia [77,78]. In the present study, calves supplemented at 1% of body weight showed downregulation of *C3* despite higher consumption of rapidly fermentable carbohydrates (starch). This finding may suggest that the insulin signaling cascade in muscle tissue is not dysregulated by high-carbohydrate intake. In addition to this reasoning, the activation of the apelin signaling pathway by upregulation of *PRKAA2* and *PRKAB2* and downregulation of *MLYCD* in supplemented calves suggests that these animals utilize more glucose as an energy source instead of fatty acids, a fact that may be closely related to changes in muscle fiber metabolism.

Skeletal muscle exhibits a certain metabolic plasticity, which allows to change substrate utilization (fat or glucose) for ATP production depending on the growth pattern of the animal [79] or nutritional stimuli [80]. During periods of accelerated muscle growth, energy expenditure for intramuscular fat and protein deposition is expected to increase [70]. Supplementation with high amounts of carbohydrates (starch) during the suckling period of calves probably stimulates glycolytic metabolism in skeletal muscle. Thus, a reduction in the fatty acid oxidation pathway for ATP production may increase the accumulation of lipids in adipose tissue. Skeletal muscle consists of a heterogeneous group of fibers that contain different myosin heavy-chain isoforms used to identify contractile and metabolic activity of muscle cells [81]. Although these isoforms were not identified in the present study, the downregulation of *CPT1A*, *CPT1B,* and *UCP3* in supplemented animals suggests a lower proportion of oxidative (type I) fibers in skeletal muscle since these transcripts are abundant in mitochondria [82]. According to Gagaoua and Picard [79], glycolytic (type II β) fibers contain a smaller number of mitochondria. These fibers are more abundant in muscles of animals with accelerated growth [83].

Fatty acid transport proteins (FATPs) are a family of six isoforms (FATP1–6). The isoform encoded by the *FATP1* gene (also known as *SLC27A1*) is highly expressed in muscle fibers, adipocytes, and hepatocytes due to the high absorption and accelerated metabolism of fatty acids in these cells [84]. In the present study, upregulation of *SLC27A1* was observed in skeletal muscle collected at weaning from animals supplemented during the cow-calf phase, and these animals thus exhibited higher IMF and MS (Table 2). These results agree with other studies on cattle that showed upregulation of *SLC27A1* in animals with higher intramuscular fat deposition [70,85]. Despite evidence that *FATP1* (*SLC27A1*) expression in muscle tissue is significantly associated with lipid accumulation [70,85,86,87], the results are contradictory since some studies have shown the opposite [88,89]. This effect on lipid metabolism (oxidation or esterification) can be explained by the localization of FATP1 that varies according to cell type and is tissue-specific [84]. For example, in muscle cells, FATP1 is most abundant in mitochondria since it is the key protein for supplying energy from fatty acid oxidation [89]. On the other hand, the cytoplasm is the site of highest abundance of FATP1 in adipocytes. When stimulated with insulin, these transporters are translocated to the plasma membrane for uptake, esterification, and accumulation of lipids [90]. An increase in FATP1 in adipose tissue increases the clearance of triglycerides from the vascular bed of muscle, thus reducing the availability of this substrate to be broken down into fatty acids [91]. Considering the negative effect of fatty acids on insulin-mediated glucose metabolism, a reduction in the flow of this substrate into muscle fibers may improve insulin sensitivity [91]. Since skeletal muscle is a heterogeneous tissue composed of muscle fibers, adipocytes, and fibroblasts, among other cell groups, the upregulated *SLC27A1* (*FATP1*) in the supplemented animals of the present study may be derived from adipocytes and not from muscle fibers. Furthermore, lower expression of *SLC27A1* in muscle fibers has been shown to be related to downregulation of *CPT1A*. Consequently, a lower rate of fatty acid oxidation in muscle fibers leads to a greater accumulation of intramuscular fat [88], if upregulation of *SLC27A1* (*FATP1*) in supplemented calves of the present study occurred in intramuscular adipose tissue. The supply of energy produced by the uptake and oxidation of fatty acids to muscle fibers may be reduced, a fact that partly explains the downregulation of pro-oxidative genes (*CPT1A, CPT1B*, and *UCP3*) in muscle of these animals.

In addition to stimulating lipogenesis, *FATP1* (*SLC27A1*) participates in the differentiation of pre-adipocytes regulated by *PPAR* [86,92]. During adipogenesis, *FATP1* increases the release of triglycerides into plasma and promotes the uptake of fatty acids by pre-adipocytes [93]. Once inside the cells, fatty acids bind to the ligand-binding domain that modifies the structure of PPAR, forming a heterodimer with the retinoid X receptor (RXR) [56]. Finally, the PPAR/RXR complex binds to the specific promoter regions of the target genes, inducing or inhibiting their expression [94]. In the present study, animals supplemented by creep feeding consumed approximately 375 g of soybean meal (Table 1). Diets containing soybean meal are rich in oleic, linoleic, and linolenic acids [58], which are potent regulators of *PPARA* [56]. It is therefore possible that, during the marbling window (250 days of age), the upregulation of *PPARA* and *RXRG* in supplemented animals is an indicator of greater recruitment of intramuscular adipocytes (adipogenesis).

The *FOXO1* gene encodes a transcription factor of the forkhead box class O family and plays an important role in the regulation of glucose metabolism through insulin signaling, in fatty acid metabolism (oxidation), and in the recruitment of pre-adipocytes in adipogenesis [95,96]. The role of *FOXO1* in adipogenesis may be either a promotor or an inhibitor depending on the stage of differentiation. Thus, in the post-mitotic stage, upregulation of *FOXO1* inhibits adipogenesis by activating the transcription of p21, a cell cycle inhibitor [97]. On the other hand, during the final differentiation and maturation of adipocytes (lipid metabolism), the inhibition of *FOXO1* impairs its binding to *PPARγ*, which leads to the formation of the *PPARγ–RXRα*–DNA complex in the transcription program [97]. In the present study, downregulation of *FOXO1* in calves supplemented during the lactation period suggests that the intramuscular adipose tissue collected at weaning may be in the final stage of differentiation and that adipocytes were ready to start fat accumulation during the finishing phase. Within this context, a larger number of differentiated intramuscular adipocytes in supplemented animals may also support the upregulation of genes related to the biosynthesis of fatty acids and cholesterol discussed in the previous paragraphs.

The *INSIG1* gene encodes an endoplasmic reticulum membrane protein that regulates glucose homeostasis and provides a negative feedback mechanism for cholesterol biosynthesis and lipogenesis [70,98]. Murine models [99] indicated that *INSIG1* expression is activated relatively late by the regulation of *PPARG* and *SREBF1* during adipogenesis and that its role in adipose tissue is to block the release of SREBP1 into the endoplasmic reticulum [100]. SREBP1 is a key protein in the control of *ACACA*, *FASN,* and *SCD* gene expression during de novo fatty acid synthesis [101]. However, the present findings do not support this mechanism since, despite the upregulation of *INSIG1* in supplemented calves, higher abundance of *FASN, SCD,* and *SCD5* was also observed, which would explain the higher IMF and MS (Table 2). Similarly, in a study on Angus and crossbred Angus × Simmental cattle, upregulation of *INSIG1* was observed in Angus animals with higher IMF and MS [70]. This fact suggests that overexpression of *INSIG1* in ruminants acts as a pro-lipogenic factor [70] and not as an anti-lipogenic gene, as demonstrated in mouse models [99].

Lipin is a protein encoded by a family of genes (*LPIN1*, *LPIN2*, and *LPIN3*) that play a key role in the lipogenesis and energy metabolism of adipose and muscle tissue [102,103]. Studies on transgenic mice with lipin deficiency or exclusive overexpression in muscle have shown that the upregulation of lipin reduces the utilization of fatty acids as energy, increasing the expression of lipogenic genes in adipocytes. Thus, the expression of lipin reduces muscle tissue energy expenditure and fat oxidation, slightly increasing the obesity of individuals [104,105]. In addition to the effects on fat deposition, altered lipin expression in muscle and adipose tissue affects insulin sensitivity. Lipin deficiency promotes insulin resistance, probably as a consequence of low leptin and adiponectin levels and impaired glucose absorption [106,107]. In contrast, lipin overexpression in transgenic mice was found to increase insulin sensitivity, although adipose tissue mass was doubled compared to normal mice [104]. This finding demonstrates that lipin improves the efficiency of fatty acid storage in adipocytes and predicts ectopic deposition of lipids in muscle tissue and consequent insulin impairment [105], in agreement with the results of the present study. Another study on cattle reported that starch-rich diets increased the expression of *LPIN2* and that this expression was positively correlated with insulin sensitivity and therefore associated with greater adipogenesis [70].

## 5. Conclusions

Supplementation of F1 Angus × Nellore calves during the cow-calf (lactation) phase prepares intramuscular adipose tissue for fat deposition during the postweaning period. The contribution of fatty acids such as linoleic and linolenic acids and high amounts of starch through ingredients such as soybean meal and ground corn included in supplements via creep feeding largely affected gene expression in the LT muscle at weaning, including adipogenic and lipogenic genes. Therefore, the upregulation of these genes that participate in critical metabolic pathways and biological processes related to PPAR signaling in adipogenesis and the insulin response, biosynthesis of fatty acids, and cholesterol in lipogenesis, contributes to the long-term intramuscular fat deposition during the finishing phase.

## Figures and Tables

**Table 1:** Means and respective standard errors of body weight at the beginning of the cow-calf phase, body weight at weaning, body weight at the end of the feedlot period, pre- and post-weaning average daily gain, and hot carcass yield obtained for the two treatments.

	BWi (kg)	WW (kg)	ADG1 (kg)	BWf (kg)	ADG2 (kg)	HCW (kg)
G1 ^1^	61.29 ± 2.41	228.92 ± 5.07 ^b^	0.93 ± 0.02 ^b^	484.64 ± 5.96	1.36 ± 0.02	269.22 ± 8.23
G2 ^1^	57.55 ± 2.61	243.57 ± 5.70 ^a^	1.03 ± 0.03 ^a^	491.85 ± 7.85	1.32 ± 0.03	273.62 ± 9.28

^1^ G1: no creep feeding, G2: creep feeding, BWi: initial body weight, WW: weaning weight, ADG1: average daily gain between WW and BWi, BWf: final body weight, ADG2: average daily gain between BWf and WW, HCW: hot carcass weight. ^a,b^ Means followed by different superscript letters significantly differ (*p* < 0.05).

**Table 2:** Means and respective standard errors of carcass backfat thickness and rib eye area, fat percentage, marbling index, and shear force at 7 and 14 days of aging obtained for the two treatments.

	BFT (mm)	IMF (%)	MS	REA (cm^2^)	WBSF7 (kg)	WBSF14 (kg)
G1 ^1^	10.61 ± 0.42 ^b^	4.95 ± 0.20 ^b^	321.50 ± 13.65 ^b^	67.94 ± 1.16	4.52 ± 0.11	3.45 ± 0.11
G2 ^1^	12.96 ± 0.83 ^a^	5.80 ± 0.23 ^a^	366.11 ± 12.39 ^a^	65.50 ± 0.93	4.28 ± 0.12	3.42 ± 0.09

^1^ G1: group 1, G2: group 2, BFT: backfat thickness, IMF: fat percentage, MS: marbling score, REA: rib eye area, WBSF7 and WBSF14: Warner–Bratzler shear force at 7 and 14 days post-mortem, respectively. ^a,b^ Means followed by different superscript letters significantly differ (*p* < 0.05).

**Table 3:** Total number of generated PE reads aligned to the reference genome, and total number and percentage of uniquely mapped PE reads in samples collected from G1 animals (no creep feeding) at weaning.

Animal/Sample	No. of Generated PE Reads	No. of Mapped PE Reads	No. of Uniquely Mapped PE Reads	% of Uniquely Mapped PE Reads
1/RC1	11,679,440	11,506,606	11,203,820	95.93
2/RC2	10,814,468	10,674,013	10,406,095	96.22
3/RC3	9,853,062	9,675,863	9,402,096	95.42
4/RC4	10,773,912	10,497,727	10,223,070	94.89
5/RC5	10,407,984	10,276,901	10,003,083	96.11
6/RC6	9,827,847	9,684,304	9,411,731	95.77
7/RC7	10,068,925	9,867,642	9,628,738	95.63
8/RC8	9,866,300	9,636,103	9,384,361	95.12
9/RC9	9,846,398	9,631,769	9,327,987	94.74
10/RC10	9,854,664	9,722,289	9,476,470	96.16
11/RC11	9,880,030	9,722,399	9,479,344	95.94
12/RC12	12,482,586	12,229,115	11,918,101	95.48
Mean	10,446,301	10,260,394	9,988,741	95.62

**Table 4:** Total number of generated PE reads aligned to the reference genome, and total number and percentage of uniquely mapped PE reads in samples collected from G2 animals (creep feeding) at weaning.

Animal/Sample	No. of Generated PE Reads	No. of Mapped PE Reads	No. of Uniquely Mapped PE Reads	% of Uniquely Mapped PE Reads
13/RC13	9,479,645	9,250,146	9,033,774	95.30
14/RC14	10,640,508	10,341,889	10,068,523	94.62
15/RC15	9,553,313	9,373,266	9,141,017	95.68
16/RC16	9,333,592	9,207,412	8,986,543	96.28
17/RC17	9,129,520	9,022,713	8,783,636	96.21
18/RC18	10,213,502	10,046,519	9,797,829	95.93
19/RC19	10,152,415	10,000,483	9,757,874	96.11
20/RC20	10,134,139	9,981,276	9,736,555	96.08
21/RC21	9,651,481	9,260,150	9,018,951	93.45
22/RC22	8,606,966	8,434,895	8,233,201	95.66
23/RC23	9,538,157	9,359,896	9,132,297	95.74
24/RC24	9,435,252	9,215,591	8,995,317	95.34
Mean	9,655,708	9,457,853	9,223,793	95.53

**Table 5:** Top 30 genes identified as differentially expressed between G1 (no creep feeding) × G2 (creep feeding) at weaning.

Gene ID Ensembl	Gene Symbol	Regulated ^1^	log_2_ FC ^2^	FDR ^3^
ENSBTAG00000017280	*C3*	Down	−2.88	2.35 × 10^−19^
ENSBTAG00000046307	*CEBPD*	Down	−2.56	2.31 × 10^−16^
ENSBTAG00000021672	*RGS1*	Down	−2.51	2.70 × 10^−16^
ENSBTAG00000048501	*ST8SIA2*	Up	2.37	8.32 × 10^−15^
ENSBTAG00000011121	*CLCN4*	Up	1.02	1.52 × 10^−14^
ENSBTAG00000014069	*PDK4*	Down	−3.34	1.52 × 10^−14^
ENSBTAG00000004248	*MLYCD*	Down	−1.89	2.63 × 10^−14^
ENSBTAG00000013242	*MYMK*	Up	1.47	2.67 × 10^−14^
ENSBTAG00000002834	*CCDC69*	Up	1.13	6.93 × 10^−14^
ENSBTAG00000022989	*FAM174B*	Up	1.12	8.62 × 10^−14^
ENSBTAG00000013631	*GLUL*	Down	−1.54	1.08 × 10^−13^
ENSBTAG00000017956	*KCTD15*	Up	1.02	1.27 × 10^−13^
ENSBTAG00000013860	*GADD45A*	Down	−1.96	1.75 × 10^−13^
ENSBTAG00000002783	*PCYOX1*	Up	0.83	6.15 × 10^−13^
ENSBTAG00000007890	*SEC14L5*	Up	2.07	6.85 × 10^−13^
ENSBTAG00000021999	*CPT1A*	Down	−1.70	7.95 × 10^−13^
ENSBTAG00000004118	*ALAS1*	Up	0.74	1.13 × 10^−12^
ENSBTAG00000046548	*ST6GALNAC4*	Up	0.86	8.42 × 10^−12^
ENSBTAG00000007578	*SHTN1*	Down	−1.69	2.05 × 10^−11^
ENSBTAG00000050158	*----------*	Up	0.86	2.05 × 10^−11^
ENSBTAG00000011909	*ACVR1*	Up	0.62	3.64 × 10^−11^
ENSBTAG00000012314	*LDLR*	Up	2.71	4.10 × 10^−11^
ENSBTAG00000015942	*DNAJA4*	Up	1.33	4.11 × 10^−11^
ENSBTAG00000014265	*SREBF2*	Up	0.90	4.15 × 10^−11^
ENSBTAG00000050852	*CXCL9*	Down	−2.34	4.23 × 10^−11^
ENSBTAG00000000163	*DDIT4*	Down	−1.16	5.15 × 10^−11^
ENSBTAG00000011437	*----------*	Down	−1.54	6.69 × 10^−11^
ENSBTAG00000016819	*FABP3*	Up	1.41	7.36 × 10^−11^
ENSBTAG00000048728	*----------*	Up	1.36	7.55 × 10^−11^
ENSBTAG00000017280	*PMEPA1*	Up	0.90	1.19 × 10^−10^

^1^ Downregulated or upregulated in G2; ^2^ log_2_ fold change; ^3^ adjusted *p*-value (FDR) obtained by edgeR used to rank the differentially expressed genes presented in the table.

**Table 6:** Enriched metabolic pathways (*p* < 0.05) highlighted in over-representation analysis of up- and down-regulated differentially expressed genes identified in G2 at weaning.

Term (KEGG)	Up/Down	No. of Genes	*p*-Value	Genes
PPAR signaling pathway	Up	9	<0.001	*FADS2*, *FABP3*, *SLC27A1*, *SCD*, *SCD5*, *AQP7*, *DBI*, *PPARA, RXRG*
Steroid biosynthesis	Up	5	<0.001	*SQLE*, *EBP*, *CYP51A1*, *DHCR24*, *LSS*
Biosynthesis of unsaturated fatty acids	Up	4	0.029	*FADS2*, *SCD*, *SCD5, FADS1*
Apelin signaling pathway	Up	8	0.039	*PRKAB2, SMAD3, PRKAA2, CCND1, MYL2, MYL3, APLNR, CALM3*
Fatty acid metabolism	Up	5	0.041	*FADS2*, *SCD*, *FASN*, *SCD5, FADS1*
AMPK signaling pathway	Down	8	0.023	*PFKFB4, CPT1A, PFKFB3, EIF4EBP1, MLYCD, CPT1B, FBP1, FOXO1*
Glucagon signaling pathway	Down	7	0.031	*CPT1A, GCGR, SIK1, CPT1B, FBP1, PLCB2, FOXO1*
PPAR signaling pathway	Down	6	0.044	*CPT1A, APOA1, ME3, ANGPTL4, CPT1B, PLIN5*

**Table 7:** Enriched biological processes (*p* < 0.05) highlighted in over-representation analysis of up- and down-regulated differentially expressed genes identified in G2 at weaning.

Term (GO_BP)	Up/Down	No. of Genes	*p*-Value	Genes
Cholesterol biosynthetic process	Up	5	0.002	*EBP, INSIG1, CYP51A1, DHCR24, LSS*
Unsaturated fatty acid biosynthetic process	Up	4	0.003	*FADS2, SCD, SCD5, FADS1*
Sterol biosynthetic process	Up	3	0.021	*SQLE, EBP, INSIG1*
Cellular response to insulin stimulus	Up	5	0.032	*GOT1, INSIG1, INHBB, LPIN2, HDAC9*
Positive regulation of lipid storage	Down	4	<0.001	*C3, IKBKE, FAM71F2, PLIN5*
Fatty acid metabolic process	Down	6	0.005	*C3, CPT1A, ACOT7, UCP3, HACL1, CPT1B*
Negative regulation of glycolytic process	Down	3	0.022	*DDIT4, NUPR1, FBP1*

## Data Availability

All relevant data are presented within the paper. RNA-Seq data may be made available by contacting the corresponding author. Data is not publicly due to privacy.

## Supplementary Materials

The following supporting information can be downloaded at: https://www.mdpi.com/article/10.3390/metabo13020160/s1. Table S1: Ingredients and chemical-bromatological composition of the feedlot diet. Table S2: Complete list of differentially expressed genes (DEGs) between groups (G1 × G2) at weaning. Table S3: Enriched KEGG pathways for upregulated genes in group 2 at weaning. Table S4: Enriched biological processes (GO terms) for upregulated genes in group 2 at weaning. Table S5: Enriched KEGG pathways for downregulated genes in group 2 at weaning. Table S6: Enriched biological processes (GO terms) for downregulated genes in group 2 at weaning. Figure S1: Visual differences in relation to the fat content between *Longissimus thoracis* muscle samples collected from animals of G1 and G2. Figure S2: Number of genes by functional category detected by RNA sequencing considering the application of a filter that excludes genes with a low relative read count after normalization. Figure S3: Log2 boxplot of the read count normalized by the size factor per sample in G1 (control, no creep feeding) and G2 (creep feeding).
