# Supplementary material for: Effect of Cow-Calf Supplementation on Gene Expression, Processes, and Pathways Related to Adipogenesis and Lipogenesis in Longissimus thoracis Muscle of F1 Angus × Nellore Cattle at Weaning
Source: Metabolites. 2023 Jan 21;13(2):160. doi: 10.3390/metabo13020160 (PMC9962728; doi:10.3390/metabo13020160)
Supplement: Supplementary file 1 [file metabolites-13-00160-s001.zip › metabolites-2140013-supplementary.pdf]

Table S1. Ingredients and chemical-bromatological composition of the feedlot diet;

**Ingredients**	**(g/kg of DM)**
Sugarcane bagasse	90
Tifton-85 hay	30
Ground corn	660.35
Soybean meal	175.3
Urea	3.80
Mineral-vitaminic supplement	37.4
**Chemical composition**	**(g/kg of DM)**
Crude Protein	153.4
Neutral Detergent Fiber	256.8
Ether Extract	35.3

DM: Dry Matter

Table S2. Complete list of differentially expressed genes (DEGs) between groups (G1 x G2) at weaning

Gene ID ensembl	Gene symbol	Gene type	log2 FC	P value	FDR	Expression
ENSBTAG00000017280	*C3*	protein-coding	-2,88718161	3,28E-23	2,35E-19	down
ENSBTAG00000046307	*CEBPD*	protein-coding	-2,566877263	4,85E-20	2,31E-16	down
ENSBTAG00000021672	*RGS1*	protein-coding	-2,518130222	7,55E-20	2,70E-16	down
ENSBTAG00000048501	*ST8SIA2*	protein-coding	2,375477906	2,91E-18	8,32E-15	up
ENSBTAG00000014069	*PDK4*	protein-coding	-3,342182588	6,36E-18	1,52E-14	down
ENSBTAG00000011121	*CLCN4*	protein-coding	1,024354349	7,41E-18	1,52E-14	up
ENSBTAG00000004248	*MLYCD*	protein-coding	-1,897125771	1,47E-17	2,63E-14	down
ENSBTAG00000013242	*MYMK*	protein-coding	1,475865923	1,68E-17	2,67E-14	up
ENSBTAG00000002834	*CCDC69*	protein-coding	1,133803659	4,84E-17	6,93E-14	up
ENSBTAG00000022989	*FAM174B*	protein-coding	1,125965312	6,62E-17	8,62E-14	up
ENSBTAG00000013631	*GLUL*	protein-coding	-1,545774043	9,04E-17	1,08E-13	down
ENSBTAG00000017956	*KCTD15*	protein-coding	1,025873817	1,15E-16	1,27E-13	up
ENSBTAG00000013860	*GADD45A*	protein-coding	-1,966398097	1,71E-16	1,75E-13	down
ENSBTAG00000002783	*PCYOX1*	protein-coding	0,837936678	6,45E-16	6,15E-13	up
ENSBTAG00000007890	*SEC14L5*	protein-coding	2,07964646	7,66E-16	6,85E-13	up
ENSBTAG00000021999	*CPT1A*	protein-coding	-1,700805903	9,44E-16	7,95E-13	down
ENSBTAG00000004118	*ALAS1*	protein-coding	0,740760051	1,42E-15	1,13E-12	up
ENSBTAG00000046548	*ST6GALNAC4*	protein-coding	0,863185363	1,12E-14	8,42E-12	up
ENSBTAG00000007578	*SHTN1*	protein-coding	-1,699762289	2,86E-14	2,05E-11	down
ENSBTAG00000050158	*NA*	NA	0,867731249	3,00E-14	2,05E-11	up
ENSBTAG00000011909	*ACVR1*	protein-coding	0,62535742	5,59E-14	3,64E-11	up
ENSBTAG00000012314	*LDLR*	protein-coding	2,713619863	6,59E-14	4,10E-11	up
ENSBTAG00000015942	*DNAJA4*	protein-coding	1,330841201	6,88E-14	4,11E-11	up
ENSBTAG00000014265	*SREBF2*	protein-coding	0,909734781	7,25E-14	4,15E-11	up
ENSBTAG00000050852	*CXCL9*	protein-coding	-2,348000597	7,68E-14	4,23E-11	down
ENSBTAG00000000163	*DDIT4*	protein-coding	-1,168143523	9,72E-14	5,15E-11	down
ENSBTAG00000011437	*SIK1*	protein-coding	-1,54764372	1,31E-13	6,69E-11	down
ENSBTAG00000016819	*FABP3*	protein-coding	1,411449047	1,49E-13	7,36E-11	up
ENSBTAG00000048728	*NA*	NA	1,369514399	1,58E-13	7,55E-11	up
ENSBTAG00000054628	*PMEPA1*	protein-coding	0,901141108	2,57E-13	1,19E-10	up
ENSBTAG00000015505	*FADS2*	protein-coding	1,337692091	3,33E-13	1,49E-10	up
ENSBTAG00000051540	*NA*	NA	0,810432371	3,91E-13	1,69E-10	up
ENSBTAG00000039190	*SLC9A5*	protein-coding	1,01730456	4,94E-13	2,08E-10	up
ENSBTAG00000053951	*SPX*	protein-coding	1,543214361	7,89E-13	3,23E-10	up
ENSBTAG00000009139	*LDLRAD4*	protein-coding	1,08020193	1,02E-12	4,05E-10	up
ENSBTAG00000046468	*ACTBL2*	protein-coding	1,670333266	1,12E-12	4,32E-10	up
ENSBTAG00000051860	*NA*	NA	1,124292448	1,39E-12	5,24E-10	up
ENSBTAG00000039335	*ARRDC2*	protein-coding	-1,617886611	1,96E-12	7,18E-10	down
ENSBTAG00000018294	*DNASE1L3*	protein-coding	-2,560923671	2,02E-12	7,23E-10	down
ENSBTAG00000020773	*CTSW*	protein-coding	-1,478110289	2,51E-12	8,76E-10	down
ENSBTAG00000016060	*CREM*	protein-coding	-1,41747262	2,62E-12	8,93E-10	down
ENSBTAG00000017718	*CCL22*	protein-coding	-3,069907623	2,89E-12	9,57E-10	down
ENSBTAG00000005259	*UCP3*	protein-coding	-1,693284565	2,94E-12	9,57E-10	down
ENSBTAG00000002473	*ANGPTL4*	protein-coding	-2,677105019	3,36E-12	1,07E-09	down
ENSBTAG00000011196	*C1QB*	protein-coding	-1,613205323	4,25E-12	1,32E-09	down
ENSBTAG00000008394	*MYL3*	protein-coding	1,559541659	4,49E-12	1,37E-09	up
ENSBTAG00000007159	*ESR1*	protein-coding	0,825858185	6,24E-12	1,86E-09	up
ENSBTAG00000030845	*MPEG1*	protein-coding	-1,395940037	7,17E-12	2,09E-09	down
ENSBTAG00000000329	*MEGF10*	protein-coding	0,798113835	7,44E-12	2,09E-09	up
ENSBTAG00000003786	*GCGR*	protein-coding	-2,788629459	7,44E-12	2,09E-09	down
ENSBTAG00000001176	*LRRN1*	protein-coding	1,17796197	8,01E-12	2,20E-09	up
ENSBTAG00000019669	*CD163*	protein-coding	-1,799704744	8,21E-12	2,22E-09	down
ENSBTAG00000025258	*LOC515676*	protein-coding	-1,993771978	9,25E-12	2,43E-09	down
ENSBTAG00000032719	*TFRC*	protein-coding	1,136473435	9,35E-12	2,43E-09	up
ENSBTAG00000003692	*UCP2*	protein-coding	-1,135749755	1,04E-11	2,65E-09	down
ENSBTAG00000000092	*HIF1AN*	protein-coding	0,613708437	1,19E-11	2,98E-09	up
ENSBTAG00000044106	*SPIDR*	protein-coding	-1,272848512	1,29E-11	3,19E-09	down
ENSBTAG00000046332	*ACTA1*	protein-coding	1,138015452	1,53E-11	3,71E-09	up
ENSBTAG00000017824	*IRF8*	protein-coding	-1,278644016	1,90E-11	4,53E-09	down
ENSBTAG00000022294	*FADS1*	protein-coding	1,52930826	2,29E-11	5,38E-09	up
ENSBTAG00000020632	*NQO1*	protein-coding	0,783901876	2,59E-11	5,97E-09	up
ENSBTAG00000008913	*TMEM98*	protein-coding	0,968379079	2,76E-11	6,28E-09	up

ENSBTAG00000010155	*LOC504773*	protein-coding	-1,411695562	3,03E-11	6,79E-09	down
ENSBTAG00000012777	*SRF*	protein-coding	0,584704637	3,19E-11	7,02E-09	up
ENSBTAG00000016683	*NFKBIA*	protein-coding	-0,991603264	4,17E-11	8,94E-09	down
ENSBTAG00000009517	*DBI*	protein-coding	0,767322876	4,18E-11	8,94E-09	up
ENSBTAG00000021143	*DNMT3A*	protein-coding	0,591863734	4,42E-11	9,30E-09	up
ENSBTAG00000019110	*PNRC1*	protein-coding	-0,934340785	5,24E-11	1,08E-08	down
ENSBTAG00000021978	*PARVB*	protein-coding	0,660723864	5,30E-11	1,08E-08	up
ENSBTAG00000032089	*CDC42EP2*	protein-coding	1,12374019	5,50E-11	1,11E-08	up
ENSBTAG00000053595	*HOXA9*	protein-coding	-0,689014174	6,40E-11	1,27E-08	down
ENSBTAG00000002885	*MSR1*	protein-coding	-1,737760726	1,00E-10	1,96E-08	down
ENSBTAG00000003033	*GADD45G*	protein-coding	-1,549481504	1,15E-10	2,22E-08	down
ENSBTAG00000007153	*C1QA*	protein-coding	-1,493387518	1,21E-10	2,30E-08	down
ENSBTAG00000018513	*FHL1*	protein-coding	0,749352137	1,22E-10	2,30E-08	up
ENSBTAG00000004824	*REEP1*	protein-coding	0,923141593	1,27E-10	2,37E-08	up
ENSBTAG00000049058	*INHBB*	protein-coding	1,098704848	1,36E-10	2,50E-08	up
ENSBTAG00000018936	*LSS*	protein-coding	0,960768516	1,42E-10	2,57E-08	up
ENSBTAG00000004688	*DHCR24*	protein-coding	1,320771166	1,48E-10	2,66E-08	up
ENSBTAG00000007268	*F13A1*	protein-coding	-1,290444644	1,66E-10	2,93E-08	down
ENSBTAG00000012919	*MMP15*	protein-coding	0,812760959	1,85E-10	3,22E-08	up
ENSBTAG00000014005	*PPIP5K1*	protein-coding	0,575692526	3,13E-10	5,39E-08	up
ENSBTAG00000017528	*SNAI3*	protein-coding	1,475491458	3,48E-10	5,93E-08	up
ENSBTAG00000018316	*ZMYND15*	protein-coding	-1,672963626	3,70E-10	6,23E-08	down
ENSBTAG00000054394	*NA*	NA	0,819614344	3,76E-10	6,25E-08	up
ENSBTAG00000022808	*CACUL1*	protein-coding	0,696289685	4,21E-10	6,91E-08	up
ENSBTAG00000048228	*SNHG12*	ncRNA	-0,769296644	4,25E-10	6,91E-08	down
ENSBTAG00000031998	*CXCL16*	protein-coding	-0,735160431	4,60E-10	7,40E-08	down
ENSBTAG00000020313	*FNBP1*	protein-coding	-0,653927867	5,58E-10	8,88E-08	down
ENSBTAG00000015214	*CA3*	protein-coding	0,98625013	5,93E-10	9,32E-08	up
ENSBTAG00000021976	*NDUFS1*	protein-coding	0,714910092	6,09E-10	9,45E-08	up
ENSBTAG00000001770	*ST8SIA5*	protein-coding	1,233953001	6,14E-10	9,45E-08	up
ENSBTAG00000031217	*MYL6B*	protein-coding	1,765015168	6,44E-10	9,80E-08	up
ENSBTAG00000000601	*COL11A2*	protein-coding	2,2749789	7,72E-10	1,16E-07	up
ENSBTAG00000020060	*TXNIP*	protein-coding	-0,924738688	8,29E-10	1,24E-07	down
ENSBTAG00000020159	*NKAIN1*	protein-coding	1,174036619	8,70E-10	1,28E-07	up
ENSBTAG00000005364	*HMBS*	protein-coding	0,651407645	9,14E-10	1,33E-07	up
ENSBTAG00000017863	*SRGN*	protein-coding	-0,953723557	9,26E-10	1,34E-07	down
ENSBTAG00000006440	*IGDCC4*	protein-coding	1,000394634	9,57E-10	1,37E-07	up
ENSBTAG00000008940	*NPTX1*	protein-coding	-3,023095421	1,04E-09	1,47E-07	down
ENSBTAG00000001078	*SRL*	protein-coding	0,687623849	1,14E-09	1,60E-07	up
ENSBTAG00000002478	*AGMAT*	protein-coding	-1,276658252	1,18E-09	1,63E-07	down
ENSBTAG00000012771	*CSF1R*	protein-coding	-1,264840129	1,19E-09	1,63E-07	down
ENSBTAG00000015405	*DCHS1*	protein-coding	0,72129315	1,27E-09	1,72E-07	up
ENSBTAG00000019788	*TEAD4*	protein-coding	0,620847661	1,28E-09	1,72E-07	up
ENSBTAG00000051744	*NA*	NA	0,841490839	1,42E-09	1,89E-07	up
ENSBTAG00000011659	*PIGZ*	protein-coding	1,185344552	1,43E-09	1,89E-07	up
ENSBTAG00000011193	*C1QC*	protein-coding	-1,349635832	1,86E-09	2,44E-07	down
ENSBTAG00000018369	*MYL2*	protein-coding	0,766297531	2,59E-09	3,37E-07	up
ENSBTAG00000015361	*CNTFR*	protein-coding	-1,297296833	2,85E-09	3,68E-07	down
ENSBTAG00000046218	*KLF11*	protein-coding	-1,637595744	3,18E-09	4,03E-07	down
ENSBTAG00000034689	*ZNRF1*	protein-coding	0,514787813	3,35E-09	4,20E-07	up
ENSBTAG00000017363	*SAT1*	protein-coding	-0,582996314	3,79E-09	4,72E-07	down
ENSBTAG00000005897	*FNTB*	protein-coding	-0,937186566	4,12E-09	5,04E-07	down
ENSBTAG00000020737	*SOX8*	protein-coding	1,126253175	4,46E-09	5,41E-07	up
ENSBTAG00000016345	*IL2RB*	protein-coding	-0,940005133	4,83E-09	5,80E-07	down
ENSBTAG00000052673	*NA*	NA	0,811570275	4,86E-09	5,80E-07	up
ENSBTAG00000033217	*TPM3*	protein-coding	0,636028574	5,07E-09	5,97E-07	up
ENSBTAG00000046333	*C6H4orf54*	protein-coding	0,981116994	5,09E-09	5,97E-07	up
ENSBTAG00000049190	*NA*	NA	0,887266666	5,18E-09	6,02E-07	up
ENSBTAG00000004249	*TANC2*	protein-coding	0,918632659	5,98E-09	6,91E-07	up
ENSBTAG00000015509	*NAMPT*	protein-coding	-1,001232044	6,53E-09	7,47E-07	down
ENSBTAG00000013341	*GYG2*	protein-coding	0,854191765	7,42E-09	8,43E-07	up
ENSBTAG00000027654	*EIF4EBP1*	protein-coding	-0,97456298	7,55E-09	8,51E-07	down
ENSBTAG00000004159	*SIX2*	protein-coding	1,105736091	7,95E-09	8,89E-07	up
ENSBTAG00000030973	*TUBA1D*	protein-coding	0,626721074	8,14E-09	8,99E-07	up
ENSBTAG00000003275	*NA*	NA	0,858045564	8,16E-09	8,99E-07	up

ENSBTAG00000047107	*TNIP3*	protein-coding	-1,295291186	8,77E-09	9,58E-07	down
ENSBTAG00000053278	*NA*	NA	0,772361609	9,07E-09	9,84E-07	up
ENSBTAG00000011102	*TPCN1*	protein-coding	0,910309366	9,16E-09	9,86E-07	up
ENSBTAG00000014898	*HK3*	protein-coding	-1,251466989	9,49E-09	1,01E-06	down
ENSBTAG00000009663	*YBX3*	protein-coding	-0,575806838	9,55E-09	1,01E-06	down
ENSBTAG00000003808	*HDAC9*	protein-coding	0,914051848	9,62E-09	1,01E-06	up
ENSBTAG00000007592	*RARG*	protein-coding	0,50065408	1,01E-08	1,05E-06	up
ENSBTAG00000054019	*NA*	NA	0,661772844	1,15E-08	1,19E-06	up
ENSBTAG00000046335	*NA*	NA	0,663042337	1,18E-08	1,22E-06	up
ENSBTAG00000049122	*COL6A5*	protein-coding	-2,933867616	1,20E-08	1,23E-06	down
ENSBTAG00000052187	*NA*	NA	-0,609712094	1,44E-08	1,45E-06	down
ENSBTAG00000011885	*NNT*	protein-coding	0,622653474	1,45E-08	1,45E-06	up
ENSBTAG00000011982	*BATF3*	protein-coding	-1,494143122	1,53E-08	1,52E-06	down
ENSBTAG00000004079	*ZNF106*	protein-coding	0,908917616	1,78E-08	1,75E-06	up
ENSBTAG00000012970	*FAM53B*	protein-coding	0,539568195	1,83E-08	1,79E-06	up
ENSBTAG00000011843	*EEF1G*	protein-coding	0,577816627	1,84E-08	1,79E-06	up
ENSBTAG00000030974	*TUBA4A*	protein-coding	0,624698785	2,10E-08	2,02E-06	up
ENSBTAG00000049408	*NA*	NA	-1,078251573	2,14E-08	2,04E-06	down
ENSBTAG00000020554	*AIF1*	protein-coding	-1,12128051	2,24E-08	2,11E-06	down
ENSBTAG00000019623	*GRID1*	protein-coding	0,760858289	2,54E-08	2,38E-06	up
ENSBTAG00000020050	*MLEC*	protein-coding	0,718051072	2,84E-08	2,64E-06	up
ENSBTAG00000006233	*NIM1K*	protein-coding	0,888235945	3,31E-08	3,06E-06	up
ENSBTAG00000002199	*CORIN*	protein-coding	0,70907678	3,35E-08	3,08E-06	up
ENSBTAG00000001305	*ATP2B2*	protein-coding	1,39607656	3,42E-08	3,12E-06	up
ENSBTAG00000011392	*MYBPC1*	protein-coding	0,516487373	3,55E-08	3,19E-06	up
ENSBTAG00000051834	*NA*	NA	0,510589745	3,68E-08	3,29E-06	up
ENSBTAG00000017719	*AKAP6*	protein-coding	0,750135699	3,88E-08	3,45E-06	up
ENSBTAG00000009951	*PGP*	protein-coding	0,669696037	3,92E-08	3,46E-06	up
ENSBTAG00000009115	*SPSB4*	protein-coding	0,810634149	4,13E-08	3,63E-06	up
ENSBTAG00000006349	*EXTL1*	protein-coding	1,155592587	4,21E-08	3,67E-06	up
ENSBTAG00000011483	*SCARF1*	protein-coding	-0,712792856	4,41E-08	3,81E-06	down
ENSBTAG00000017715	*HOMER2*	protein-coding	0,669315588	4,60E-08	3,94E-06	up
ENSBTAG00000014396	*KLF10*	protein-coding	-0,950498177	4,93E-08	4,18E-06	down
ENSBTAG00000012228	*SEMA4A*	protein-coding	-1,159469601	4,93E-08	4,18E-06	down
ENSBTAG00000032051	*SULT1C4*	protein-coding	-1,293246698	4,97E-08	4,18E-06	down
ENSBTAG00000012509	*DYRK1B*	protein-coding	0,533224177	5,23E-08	4,37E-06	up
ENSBTAG00000011182	*PDLIM1*	protein-coding	0,652420055	5,28E-08	4,37E-06	up
ENSBTAG00000008240	*GALNT15*	protein-coding	-1,637316162	5,28E-08	4,37E-06	down
ENSBTAG00000046153	*NA*	NA	-0,999320432	5,36E-08	4,41E-06	down
ENSBTAG00000007079	*LCP1*	protein-coding	-0,522197337	5,58E-08	4,57E-06	down
ENSBTAG00000020223	*CASQ1*	protein-coding	0,669536372	5,85E-08	4,73E-06	up
ENSBTAG00000009631	*ZNF683*	protein-coding	-1,08903761	5,85E-08	4,73E-06	down
ENSBTAG00000026779	*LYZ*	protein-coding	-1,298173681	6,22E-08	5,00E-06	down
ENSBTAG00000014583	*CALM3*	protein-coding	0,652601382	6,31E-08	5,05E-06	up
ENSBTAG00000019146	*OSBP2*	protein-coding	0,99292305	6,45E-08	5,13E-06	up
ENSBTAG00000055207	*SCD*	protein-coding	1,35560706	6,68E-08	5,28E-06	up
ENSBTAG00000011400	*DBN1*	protein-coding	0,658889864	6,79E-08	5,32E-06	up
ENSBTAG00000012259	*PNPO*	protein-coding	0,695744549	6,81E-08	5,32E-06	up
ENSBTAG00000006561	*RASGEF1B*	protein-coding	-0,916358453	7,33E-08	5,70E-06	down
ENSBTAG00000047502	*FKBP5*	protein-coding	-1,555363477	7,71E-08	5,96E-06	down
ENSBTAG00000017069	*GASK1B*	protein-coding	0,696280568	7,79E-08	5,99E-06	up
ENSBTAG00000008204	*CARNMT1*	protein-coding	0,72111271	7,92E-08	6,06E-06	up
ENSBTAG00000007090	*MYH2*	protein-coding	0,796362032	8,84E-08	6,73E-06	up
ENSBTAG00000047412	*TNFSF18*	protein-coding	1,334703592	9,01E-08	6,79E-06	up
ENSBTAG00000021872	*RASGRP1*	protein-coding	0,720968957	9,02E-08	6,79E-06	up
ENSBTAG00000036260	*LPXN*	protein-coding	-1,083849408	9,50E-08	7,12E-06	down
ENSBTAG00000010877	*ARMC12*	protein-coding	-2,049332701	9,61E-08	7,16E-06	down
ENSBTAG00000018747	*PRKAA2*	protein-coding	0,658033056	1,01E-07	7,52E-06	up
ENSBTAG00000016240	*RNF157*	protein-coding	0,527491146	1,07E-07	7,86E-06	up
ENSBTAG00000009159	*PLXNA1*	protein-coding	0,611246738	1,12E-07	8,20E-06	up
ENSBTAG00000012163	*ITGB1BP2*	protein-coding	0,665717324	1,14E-07	8,29E-06	up
ENSBTAG00000053969	*NA*	NA	0,629698026	1,26E-07	9,07E-06	up
ENSBTAG00000001592	*INSIG1*	protein-coding	1,24680273	1,27E-07	9,14E-06	up
ENSBTAG00000035998	*CKB*	protein-coding	-1,418030637	1,32E-07	9,42E-06	down
ENSBTAG00000023074	*C28H1orf198*	protein-coding	0,502510228	1,36E-07	9,71E-06	up

ENSBTAG00000010411	*ADAMTS8*	protein-coding	2,821314632	1,37E-07	9,71E-06	up
ENSBTAG00000010978	*PROX1*	protein-coding	0,85532529	1,41E-07	9,93E-06	up
ENSBTAG00000038048	*MRC1*	protein-coding	-1,213348429	1,44E-07	1,01E-05	down
ENSBTAG00000007450	*C2*	protein-coding	-1,412846723	1,45E-07	1,01E-05	down
ENSBTAG00000054241	*LOC100300896*	protein-coding	-1,078263672	1,48E-07	1,03E-05	down
ENSBTAG00000011580	*DAG1*	protein-coding	0,520630434	1,54E-07	1,07E-05	up
ENSBTAG00000009239	*SLC16A10*	protein-coding	0,615780741	1,60E-07	1,10E-05	up
ENSBTAG00000046544	*SLC7A10*	protein-coding	1,10698262	1,81E-07	1,24E-05	up
ENSBTAG00000038889	*RILP*	protein-coding	-0,514639994	1,87E-07	1,28E-05	down
ENSBTAG00000037765	*TNFAIP8L1*	protein-coding	0,776727348	2,09E-07	1,40E-05	up
ENSBTAG00000001471	*POC1A*	protein-coding	-0,716858523	2,20E-07	1,46E-05	down
ENSBTAG00000000894	*PGK1*	protein-coding	0,767422387	2,24E-07	1,49E-05	up
ENSBTAG00000017509	*MYPN*	protein-coding	0,526640654	2,33E-07	1,54E-05	up
ENSBTAG00000021669	*SOGA1*	protein-coding	0,541861557	2,49E-07	1,63E-05	up
ENSBTAG00000024291	*FAM71F2*	protein-coding	-1,598143247	2,58E-07	1,68E-05	down
ENSBTAG00000002362	*APOLD1*	protein-coding	-0,952754882	2,59E-07	1,68E-05	down
ENSBTAG00000023600	*APOD*	protein-coding	-0,519057832	2,70E-07	1,73E-05	down
ENSBTAG00000054721	*NA*	NA	0,64727468	2,71E-07	1,73E-05	up
ENSBTAG00000011088	*SLC6A6*	protein-coding	-0,979634214	2,76E-07	1,75E-05	down
ENSBTAG00000047420	*BTNL9*	protein-coding	-1,120421158	2,91E-07	1,84E-05	down
ENSBTAG00000013306	*ZBTB7C*	protein-coding	0,601006247	3,45E-07	2,17E-05	up
ENSBTAG00000005498	*SQLE*	protein-coding	0,999112079	3,54E-07	2,19E-05	up
ENSBTAG00000053570	*NA*	NA	0,541326238	3,71E-07	2,29E-05	up
ENSBTAG00000014387	*PRKAB2*	protein-coding	0,651602908	3,73E-07	2,29E-05	up
ENSBTAG00000034366	*RGS2*	protein-coding	-1,02238794	3,83E-07	2,33E-05	down
ENSBTAG00000010361	*DLL4*	protein-coding	0,837377651	3,84E-07	2,33E-05	up
ENSBTAG00000019079	*PLCB2*	protein-coding	-1,06307285	3,93E-07	2,37E-05	down
ENSBTAG00000004792	*CHMP4C*	protein-coding	0,63610589	3,94E-07	2,37E-05	up
ENSBTAG00000007665	*NPR3*	protein-coding	1,844228383	3,96E-07	2,37E-05	up
ENSBTAG00000005871	*MECOM*	protein-coding	0,60233278	3,98E-07	2,37E-05	up
ENSBTAG00000007439	*TENM4*	protein-coding	1,146696029	4,12E-07	2,44E-05	up
ENSBTAG00000027246	*UBD*	protein-coding	-0,945770564	4,42E-07	2,61E-05	down
ENSBTAG00000016775	*SLC27A1*	protein-coding	0,515673662	4,61E-07	2,70E-05	up
ENSBTAG00000012451	*BOLA-DMB*	protein-coding	-0,589499864	4,64E-07	2,70E-05	down
ENSBTAG00000014357	*SDC2*	protein-coding	0,61025531	4,65E-07	2,70E-05	up
ENSBTAG00000008735	*VASH1*	protein-coding	1,100227535	4,73E-07	2,74E-05	up
ENSBTAG00000001003	*CKMT2*	protein-coding	0,5873481	4,76E-07	2,74E-05	up
ENSBTAG00000008063	*PPARA*	protein-coding	0,780455815	4,78E-07	2,74E-05	up
ENSBTAG00000021508	*LMOD3*	protein-coding	0,560284777	4,81E-07	2,74E-05	up
ENSBTAG00000000804	*MOB3B*	protein-coding	-0,738526726	4,81E-07	2,74E-05	down
ENSBTAG00000050863	*NA*	NA	-1,600682568	5,02E-07	2,84E-05	down
ENSBTAG00000004177	*TANC1*	protein-coding	0,506744996	5,05E-07	2,85E-05	up
ENSBTAG00000053775	*NA*	NA	-0,663852188	5,47E-07	3,06E-05	down
ENSBTAG00000010994	*CTSF*	protein-coding	-0,57990403	5,48E-07	3,06E-05	down
ENSBTAG00000006817	*CBL*	protein-coding	0,698807042	5,79E-07	3,23E-05	up
ENSBTAG00000013004	*ITIH5*	protein-coding	0,585167761	5,82E-07	3,23E-05	up
ENSBTAG00000020219	*MSS51*	protein-coding	0,831812503	6,33E-07	3,49E-05	up
ENSBTAG00000053812	*NA*	NA	0,939347031	6,52E-07	3,58E-05	up
ENSBTAG00000048488	*NA*	NA	0,55929227	6,93E-07	3,79E-05	up
ENSBTAG00000001842	*GSTM3*	protein-coding	-1,264616003	7,04E-07	3,83E-05	down
ENSBTAG00000017081	*MIEF2*	protein-coding	0,56627132	7,63E-07	4,11E-05	up
ENSBTAG00000040347	*GPC6*	protein-coding	-0,579715473	7,64E-07	4,11E-05	down
ENSBTAG00000033679	*HLCS*	protein-coding	0,55394624	7,69E-07	4,12E-05	up
ENSBTAG00000004564	*MBNL1*	protein-coding	-0,527023505	8,07E-07	4,31E-05	down
ENSBTAG00000019574	*MAPK12*	protein-coding	-0,541852503	8,19E-07	4,34E-05	down
ENSBTAG00000018594	*LIN37*	protein-coding	-0,515692403	8,29E-07	4,38E-05	down
ENSBTAG00000003066	*NSA2*	protein-coding	-0,556537205	8,35E-07	4,39E-05	down
ENSBTAG00000016991	*EFNB2*	protein-coding	0,593755967	8,40E-07	4,40E-05	up
ENSBTAG00000054218	*IGFBP5*	protein-coding	1,086494129	8,91E-07	4,64E-05	up
ENSBTAG00000002515	*TNNI3*	protein-coding	1,374718534	9,07E-07	4,70E-05	up
ENSBTAG00000018572	*FLT3*	protein-coding	-1,617944958	9,34E-07	4,81E-05	down
ENSBTAG00000023289	*SLC26A10*	protein-coding	-0,68668429	9,35E-07	4,81E-05	down
ENSBTAG00000047155	*C28H10orf71*	protein-coding	0,547880705	9,54E-07	4,89E-05	up
ENSBTAG00000025210	*COL4A2*	protein-coding	0,539011543	1,03E-06	5,24E-05	up
ENSBTAG00000002203	*NDUFS2*	protein-coding	0,502410194	1,07E-06	5,44E-05	up

ENSBTAG00000006619	*MATK*	protein-coding	-1,02088975	1,12E-06	5,68E-05	down
ENSBTAG00000019900	*LNPEP*	protein-coding	0,657558845	1,13E-06	5,68E-05	up
ENSBTAG00000022255	*AR*	protein-coding	0,87727431	1,13E-06	5,68E-05	up
ENSBTAG00000052746	*NA*	NA	0,892864689	1,13E-06	5,68E-05	up
ENSBTAG00000039080	*SLC22A3*	protein-coding	-1,842888551	1,16E-06	5,74E-05	down
ENSBTAG00000013496	*CPM*	protein-coding	-0,780241989	1,18E-06	5,83E-05	down
ENSBTAG00000008688	*HEYL*	protein-coding	0,507701808	1,22E-06	6,01E-05	up
ENSBTAG00000003074	*SLC16A6*	protein-coding	-1,281475595	1,22E-06	6,01E-05	down
ENSBTAG00000001725	*CXCL10*	protein-coding	-1,339962918	1,23E-06	6,05E-05	down
ENSBTAG00000011500	*CASQ2*	protein-coding	1,121365175	1,30E-06	6,34E-05	up
ENSBTAG00000011338	*NREP*	protein-coding	0,809988467	1,34E-06	6,51E-05	up
ENSBTAG00000000895	*TAF9B*	protein-coding	0,698465058	1,36E-06	6,56E-05	up
ENSBTAG00000019121	*IFT122*	protein-coding	0,579911135	1,44E-06	6,93E-05	up
ENSBTAG00000016048	*CPT1B*	protein-coding	-0,635590912	1,45E-06	6,95E-05	down
ENSBTAG00000046122	*NRARP*	protein-coding	0,932489986	1,49E-06	7,11E-05	up
ENSBTAG00000003889	*PER1*	protein-coding	-0,808053091	1,52E-06	7,18E-05	down
ENSBTAG00000038238	*LOC101902340*	pseudo	0,930646289	1,53E-06	7,21E-05	up
ENSBTAG00000017256	*CD2*	protein-coding	-1,001744326	1,79E-06	8,30E-05	down
ENSBTAG00000017071	*C1QTNF3*	protein-coding	0,850462324	1,92E-06	8,92E-05	up
ENSBTAG00000033367	*DDO*	protein-coding	-0,769745366	2,13E-06	9,78E-05	down
ENSBTAG00000038844	*ANKRD35*	protein-coding	0,771324698	2,14E-06	9,78E-05	up
ENSBTAG00000037500	*MIR1842*	ncRNA	0,604131238	2,18E-06	9,86E-05	up
ENSBTAG00000020141	*FREM1*	protein-coding	0,729847386	2,18E-06	9,86E-05	up
ENSBTAG00000007506	*CACNG7*	protein-coding	1,289468375	2,22E-06	0,000100172	up
ENSBTAG00000014465	*SERPINE1*	protein-coding	-1,072072097	2,23E-06	0,000100172	down
ENSBTAG00000027676	*IL18BP*	protein-coding	-0,697751975	2,32E-06	0,00010381	down
ENSBTAG00000017938	*LOC112449293*	protein-coding	1,398693488	2,34E-06	0,000104395	up
ENSBTAG00000015769	*VSIG4*	protein-coding	-1,783446589	2,36E-06	0,000104609	down
ENSBTAG00000001193	*UNC93B1*	protein-coding	-0,67619997	2,39E-06	0,000105635	down
ENSBTAG00000054808	*MT1A*	protein-coding	-2,177883684	2,55E-06	0,00011104	down
ENSBTAG00000007635	*PLCL1*	protein-coding	0,533301754	2,59E-06	0,000112111	up
ENSBTAG00000020126	*MYO10*	protein-coding	0,574504417	2,63E-06	0,000113922	up
ENSBTAG00000048959	*LOC786372*	protein-coding	-0,800236072	2,65E-06	0,000114252	down
ENSBTAG00000034885	*RGCC*	protein-coding	-0,760366526	2,68E-06	0,000115285	down
ENSBTAG00000015996	*GPC1*	protein-coding	0,508266831	2,78E-06	0,000119135	up
ENSBTAG00000054012	*NA*	NA	0,579258889	2,89E-06	0,000122644	up
ENSBTAG00000006491	*AGL*	protein-coding	0,661917526	2,90E-06	0,000122644	up
ENSBTAG00000016396	*PDE11A*	protein-coding	1,275252683	2,96E-06	0,000124698	up
ENSBTAG00000013337	*DHDH*	protein-coding	0,777316128	2,97E-06	0,000124698	up
ENSBTAG00000050644	*NA*	NA	-0,522668402	3,09E-06	0,000129495	down
ENSBTAG00000012263	*ASAP3*	protein-coding	0,663540409	3,53E-06	0,000145635	up
ENSBTAG00000040190	*CCDC88B*	protein-coding	-1,095421581	3,53E-06	0,000145635	down
ENSBTAG00000054895	*LOC112445469*	pseudo	-1,326590595	3,54E-06	0,000145635	down
ENSBTAG00000008793	*RNASE1*	protein-coding	-0,934752751	3,62E-06	0,000147854	down
ENSBTAG00000047449	*LOC104968634*	protein-coding	-0,952496382	3,65E-06	0,000149012	down
ENSBTAG00000001197	*SLAMF7*	protein-coding	-0,932931335	3,70E-06	0,000150415	down
ENSBTAG00000030426	*LOC100848575*	protein-coding	-0,909223443	3,71E-06	0,000150415	down
ENSBTAG00000051972	*CEBPB*	protein-coding	-0,597635996	3,87E-06	0,000155812	down
ENSBTAG00000006419	*TNNT1*	protein-coding	0,558128912	3,97E-06	0,000158525	up
ENSBTAG00000021474	*GSDMD*	protein-coding	-0,603847621	3,98E-06	0,00015869	down
ENSBTAG00000008739	*SAMD11*	protein-coding	0,817800583	4,15E-06	0,000164521	up
ENSBTAG00000004742	*RUNX1*	protein-coding	-0,982364477	4,23E-06	0,00016728	down
ENSBTAG00000001061	*PRKCA*	protein-coding	0,579840529	4,24E-06	0,00016735	up
ENSBTAG00000018167	*NA*	NA	0,652841431	4,64E-06	0,00018088	up
ENSBTAG00000008437	*PCDH12*	protein-coding	1,1501743	4,80E-06	0,000186076	up
ENSBTAG00000017616	*ADSS1*	protein-coding	0,56203486	4,88E-06	0,000188619	up
ENSBTAG00000010507	*SLC22A16*	protein-coding	-1,019488707	4,97E-06	0,000191074	down
ENSBTAG00000048699	*NA*	NA	-1,072346968	5,08E-06	0,00019449	down
ENSBTAG00000018082	*QPRT*	protein-coding	1,182007814	5,14E-06	0,000196065	up
ENSBTAG00000009703	*MYH7*	protein-coding	0,59904503	5,19E-06	0,000197431	up
ENSBTAG00000025136	*MYOZ3*	protein-coding	0,610103677	5,32E-06	0,000201695	up
ENSBTAG00000052443	*NA*	NA	0,704141496	5,33E-06	0,000201695	up
ENSBTAG00000012135	*LARP1B*	protein-coding	0,603298316	5,44E-06	0,0002055	up
ENSBTAG00000012322	*INKA1*	protein-coding	0,613280883	5,58E-06	0,000209534	up
ENSBTAG00000001063	*HOXA4*	protein-coding	-0,678674482	5,58E-06	0,000209534	down

ENSBTAG00000010442	*PANK1*	protein-coding	0,806082761	5,59E-06	0,000209534	up
ENSBTAG00000021141	*CD8A*	protein-coding	-1,027907929	5,61E-06	0,000209646	down
ENSBTAG00000001051	*NA*	NA	-0,969153393	5,68E-06	0,000211668	down
ENSBTAG00000027684	*FOLR2*	protein-coding	-1,268654564	5,84E-06	0,000217195	down
ENSBTAG00000051102	*KCNK3*	protein-coding	-1,643575396	5,90E-06	0,000218089	down
ENSBTAG00000037921	*OVCH2*	protein-coding	1,101635749	6,15E-06	0,000225528	up
ENSBTAG00000012007	*SOCS2*	protein-coding	-0,659515437	6,15E-06	0,000225528	down
ENSBTAG00000038735	*LHB*	protein-coding	0,822296649	6,18E-06	0,000225528	up
ENSBTAG00000002037	*ATXN7L1*	protein-coding	-0,675654061	6,21E-06	0,000226237	down
ENSBTAG00000012969	*DKK2*	protein-coding	1,039122038	6,38E-06	0,000231329	up
ENSBTAG00000027151	*ARHGAP26*	protein-coding	-0,644735553	6,62E-06	0,000237333	down
ENSBTAG00000020602	*IDO1*	protein-coding	-0,808562232	6,63E-06	0,000237333	down
ENSBTAG00000053365	*CD300E*	protein-coding	-1,58047718	6,92E-06	0,000246209	down
ENSBTAG00000052404	*NA*	NA	0,715202202	7,12E-06	0,000252221	up
ENSBTAG00000019033	*CD84*	protein-coding	-1,083105584	7,25E-06	0,00025626	down
ENSBTAG00000003219	*NA*	NA	0,587749392	7,52E-06	0,000263888	up
ENSBTAG00000017871	*GNA12*	protein-coding	0,649295549	8,26E-06	0,000284985	up
ENSBTAG00000006579	*P4HA3*	protein-coding	-1,577620254	8,38E-06	0,00028821	down
ENSBTAG00000014466	*VTCN1*	protein-coding	0,85992335	8,46E-06	0,000289545	up
ENSBTAG00000053898	*NA*	NA	0,965658708	8,80E-06	0,000300654	up
ENSBTAG00000052514	*LOC100336869*	protein-coding	-1,168134665	9,09E-06	0,000308374	down
ENSBTAG00000009417	*ZFAND5*	protein-coding	-0,630227874	9,26E-06	0,000310923	down
ENSBTAG00000010284	*ETNPPL*	protein-coding	0,658438032	9,30E-06	0,00031103	up
ENSBTAG00000010943	*SLC22A23*	protein-coding	0,548171478	9,30E-06	0,00031103	up
ENSBTAG00000032140	*SBK1*	protein-coding	0,925801371	9,59E-06	0,000319808	up
ENSBTAG00000021310	*NA*	NA	1,00916434	9,85E-06	0,000326871	up
ENSBTAG00000017994	*MAX*	protein-coding	-0,668194114	9,87E-06	0,000326871	down
ENSBTAG00000008294	*KCNJ2*	protein-coding	0,548945504	1,01E-05	0,000333737	up
ENSBTAG00000045943	*CHRNG*	protein-coding	0,870669381	1,01E-05	0,000334377	up
ENSBTAG00000006752	*PFKFB4*	protein-coding	-0,724970203	1,03E-05	0,000338381	down
ENSBTAG00000011810	*ANGPTL2*	protein-coding	-0,503131891	1,04E-05	0,000339855	down
ENSBTAG00000012757	*GCNT1*	protein-coding	-0,699478862	1,08E-05	0,000352305	down
ENSBTAG00000008535	*SOCS7*	protein-coding	0,55979153	1,10E-05	0,000358325	up
ENSBTAG00000036028	*BEX2*	protein-coding	0,739768784	1,13E-05	0,000365119	up
ENSBTAG00000010637	*ARSG*	protein-coding	-1,170316087	1,14E-05	0,000366878	down
ENSBTAG00000052982	*NA*	NA	0,746058654	1,17E-05	0,000375855	up
ENSBTAG00000045604	*TTC9*	protein-coding	-0,626420674	1,18E-05	0,000377953	down
ENSBTAG00000009206	*FOXS1*	protein-coding	-0,698172008	1,21E-05	0,000385485	down
ENSBTAG00000032292	*CCDC159*	protein-coding	1,071478101	1,23E-05	0,000390024	up
ENSBTAG00000048650	*NA*	NA	0,830411172	1,25E-05	0,000394749	up
ENSBTAG00000011803	*MYH3*	protein-coding	0,643503208	1,27E-05	0,000397905	up
ENSBTAG00000021045	*E2F3*	protein-coding	0,523972019	1,28E-05	0,000399847	up
ENSBTAG00000018723	*SLC25A34*	protein-coding	-0,575373631	1,28E-05	0,000400198	down
ENSBTAG00000004048	*MCHR1*	protein-coding	-1,0195736	1,30E-05	0,000405243	down
ENSBTAG00000001465	*P2RY1*	protein-coding	0,814819923	1,32E-05	0,000410015	up
ENSBTAG00000005604	*POPDC2*	protein-coding	0,764306041	1,35E-05	0,000416408	up
ENSBTAG00000008061	*RILPL1*	protein-coding	-0,595803682	1,35E-05	0,000416408	down
ENSBTAG00000026408	*NA*	NA	0,632110003	1,36E-05	0,000419766	up
ENSBTAG00000018349	*IFI30*	protein-coding	-0,986621702	1,38E-05	0,000425216	down
ENSBTAG00000021048	*ADM*	protein-coding	-0,609995557	1,40E-05	0,000430443	down
ENSBTAG00000052455	*MIR12056*	ncRNA	0,658817981	1,41E-05	0,0004312	up
ENSBTAG00000003015	*SESN1*	protein-coding	-0,692521854	1,41E-05	0,0004312	down
ENSBTAG00000009839	*GSTK1*	protein-coding	0,532303387	1,45E-05	0,00043887	up
ENSBTAG00000014229	*SYT17*	protein-coding	0,974315901	1,47E-05	0,000443187	up
ENSBTAG00000010366	*HCRTR1*	protein-coding	-0,998579051	1,49E-05	0,000445345	down
ENSBTAG00000023429	*PLS1*	protein-coding	0,718343153	1,51E-05	0,000448392	up
ENSBTAG00000052359	*NA*	NA	0,540575	1,52E-05	0,000449465	up
ENSBTAG00000050535	*ZNF385D*	protein-coding	0,700207127	1,59E-05	0,000466216	up
ENSBTAG00000007417	*GDF11*	protein-coding	0,726028949	1,59E-05	0,000466216	up
ENSBTAG00000000575	*TNC*	protein-coding	-1,271716175	1,64E-05	0,000478815	down
ENSBTAG00000001060	*CXCR4*	protein-coding	0,660429378	1,65E-05	0,000479651	up
ENSBTAG00000020330	*BTG1*	protein-coding	-0,687737675	1,65E-05	0,000479651	down
ENSBTAG00000007281	*SEMA6C*	protein-coding	0,788513115	1,70E-05	0,00049173	up
ENSBTAG00000033315	*DNAJC1*	protein-coding	-0,514657593	1,70E-05	0,00049173	down
ENSBTAG00000008006	*RASGRP3*	protein-coding	0,602796676	1,71E-05	0,000493513	up

ENSBTAG00000019203	*S100A4*	protein-coding	-0,75117395	1,72E-05	0,000494415	down
ENSBTAG00000020829	*PTPRK*	protein-coding	-0,582451091	1,73E-05	0,00049485	down
ENSBTAG00000008409	*MYC*	protein-coding	-0,835378744	1,80E-05	0,000514197	down
ENSBTAG00000005666	*LRRC20*	protein-coding	0,545538626	1,84E-05	0,000522247	up
ENSBTAG00000050967	*AADAT*	protein-coding	0,509152564	1,89E-05	0,000534301	up
ENSBTAG00000023028	*WDFY4*	protein-coding	-0,584708104	1,96E-05	0,000548171	down
ENSBTAG00000047755	*FNDC10*	protein-coding	0,842276262	1,97E-05	0,000548171	up
ENSBTAG00000019901	*RAD54L*	protein-coding	0,973531598	1,98E-05	0,000551851	up
ENSBTAG00000015386	*REL*	protein-coding	0,841546454	1,99E-05	0,000554294	up
ENSBTAG00000027181	*LAMA3*	protein-coding	0,619570184	2,03E-05	0,000563385	up
ENSBTAG00000015681	*RGR*	protein-coding	2,912751715	2,12E-05	0,000583654	up
ENSBTAG00000010742	*PKD2L1*	protein-coding	1,500166177	2,13E-05	0,000584302	up
ENSBTAG00000013573	*BIRC5*	protein-coding	0,834231886	2,15E-05	0,000590206	up
ENSBTAG00000009341	*CCDC146*	protein-coding	-0,520090387	2,17E-05	0,000592754	down
ENSBTAG00000015339	*ENDOV*	protein-coding	-0,961440535	2,23E-05	0,000606049	down
ENSBTAG00000000995	*TENT5A*	protein-coding	-0,747505205	2,25E-05	0,000610779	down
ENSBTAG00000012060	*LPIN2*	protein-coding	0,558469319	2,26E-05	0,000610779	up
ENSBTAG00000016801	*RXRG*	protein-coding	0,646399783	2,32E-05	0,000626078	up
ENSBTAG00000003177	*SLC25A33*	protein-coding	-0,897301355	2,34E-05	0,000628362	down
ENSBTAG00000008169	*C24H18orf54*	protein-coding	-0,562252422	2,34E-05	0,000629418	down
ENSBTAG00000045862	*FERMT3*	protein-coding	-0,651349517	2,36E-05	0,000632002	down
ENSBTAG00000033221	*CCDC152*	protein-coding	-0,680010115	2,37E-05	0,000633956	down
ENSBTAG00000012519	*XDH*	protein-coding	-0,777670991	2,41E-05	0,000640982	down
ENSBTAG00000050392	*FAM171B*	protein-coding	-0,836475628	2,61E-05	0,000688193	down
ENSBTAG00000054688	*SOX7*	protein-coding	0,50522845	2,64E-05	0,000694014	up
ENSBTAG00000052707	*NA*	NA	0,601508034	2,65E-05	0,000694624	up
ENSBTAG00000004735	*CCNE1*	protein-coding	0,578765427	2,69E-05	0,000702752	up
ENSBTAG00000009733	*FBP1*	protein-coding	-1,259915628	2,77E-05	0,000713574	down
ENSBTAG00000002894	*TNFSF4*	protein-coding	1,027336952	2,78E-05	0,000714233	up
ENSBTAG00000001881	*SLC10A1*	protein-coding	-0,728508219	2,78E-05	0,000714233	down
ENSBTAG00000002092	*PI16*	protein-coding	0,709610823	2,79E-05	0,000715533	up
ENSBTAG00000046158	*CFB*	protein-coding	-1,382319953	2,79E-05	0,000715533	down
ENSBTAG00000017450	*KLHL24*	protein-coding	0,543558682	2,88E-05	0,000735558	up
ENSBTAG00000051111	*NA*	NA	0,757147145	3,02E-05	0,000765181	up
ENSBTAG00000004126	*MLF1*	protein-coding	0,531620407	3,05E-05	0,000768421	up
ENSBTAG00000005048	*DHRS7C*	protein-coding	0,590264746	3,07E-05	0,000772598	up
ENSBTAG00000017223	*MEST*	protein-coding	0,762736504	3,10E-05	0,000778924	up
ENSBTAG00000050334	*CD83*	protein-coding	-1,020170588	3,22E-05	0,00080554	down
ENSBTAG00000002699	*KIT*	protein-coding	0,682341427	3,25E-05	0,000811288	up
ENSBTAG00000033731	*PRCP*	protein-coding	0,52739454	3,28E-05	0,000817993	up
ENSBTAG00000052838	*NA*	NA	0,648688023	3,34E-05	0,000832133	up
ENSBTAG00000008954	*PSMB9*	protein-coding	-0,538249645	3,35E-05	0,0008324	down
ENSBTAG00000004394	*PKIB*	protein-coding	-1,036862662	3,39E-05	0,000840448	down
ENSBTAG00000052248	*NA*	NA	0,567552707	3,48E-05	0,000858339	up
ENSBTAG00000049855	*NA*	NA	-0,697424482	3,49E-05	0,000860803	down
ENSBTAG00000008571	*CUX2*	protein-coding	1,099640718	3,58E-05	0,000879053	up
ENSBTAG00000022169	*PREX2*	protein-coding	0,501638065	3,61E-05	0,000879545	up
ENSBTAG00000010849	*ANKRD23*	protein-coding	0,540686997	3,61E-05	0,000879545	up
ENSBTAG00000002983	*NT5C1A*	protein-coding	0,665503991	3,62E-05	0,000879545	up
ENSBTAG00000010760	*REV3L*	protein-coding	-0,525981856	3,65E-05	0,000884532	down
ENSBTAG00000020696	*HECW2*	protein-coding	0,630570046	3,78E-05	0,000911642	up
ENSBTAG00000020037	*CYB5RL*	protein-coding	0,607513972	3,81E-05	0,000918521	up
ENSBTAG00000051082	*DST*	protein-coding	0,502457658	3,98E-05	0,000955208	up
ENSBTAG00000004862	*TUB*	protein-coding	0,869665875	4,00E-05	0,000957184	up
ENSBTAG00000050493	*NA*	NA	-0,701417567	4,00E-05	0,000957184	down
ENSBTAG00000021073	*KIAA1549*	protein-coding	0,649160802	4,08E-05	0,000971562	up
ENSBTAG00000009353	*EVI2B*	protein-coding	-0,78269647	4,09E-05	0,000971805	down
ENSBTAG00000039196	*LOC510860*	protein-coding	-1,638872386	4,14E-05	0,000976444	down
ENSBTAG00000010138	*SEMA3B*	protein-coding	-0,616830917	4,14E-05	0,000976444	down
ENSBTAG00000014831	*PPP1R3C*	protein-coding	0,993613778	4,15E-05	0,000976444	up
ENSBTAG00000044606	*MIR2382*	ncRNA	0,582217743	4,16E-05	0,000976444	up
ENSBTAG00000013666	*SLC25A29*	protein-coding	0,599551237	4,17E-05	0,000977842	up
ENSBTAG00000019230	*PTGER3*	protein-coding	-1,863746627	4,20E-05	0,000982445	down
ENSBTAG00000036127	*AS3MT*	protein-coding	0,585022946	4,64E-05	0,001070411	up
ENSBTAG00000008636	*PDE4B*	protein-coding	-0,665427843	4,66E-05	0,001073338	down

ENSBTAG00000014224	*IGSF9B*	protein-coding	1,415607868	4,67E-05	0,0010746	up
ENSBTAG00000009846	*MTHFD1L*	protein-coding	-0,723901783	4,79E-05	0,00109641	down
ENSBTAG00000051103	*NA*	NA	0,706855996	4,80E-05	0,001098127	up
ENSBTAG00000012738	*ZNF827*	protein-coding	0,732178506	4,85E-05	0,00110682	up
ENSBTAG00000026326	*SPN*	protein-coding	-0,745972221	4,95E-05	0,001125371	down
ENSBTAG00000018248	*MGLL*	protein-coding	-0,550457719	5,02E-05	0,001139738	down
ENSBTAG00000049378	*NA*	NA	1,079973298	5,05E-05	0,001142749	up
ENSBTAG00000007668	*AGPAT4*	protein-coding	0,875617236	5,06E-05	0,00114509	up
ENSBTAG00000008714	*CLSTN2*	protein-coding	0,68149513	5,25E-05	0,00118341	up
ENSBTAG00000051237	*NA*	NA	-1,083490031	5,31E-05	0,00119432	down
ENSBTAG00000002096	*FCGR3A*	protein-coding	-0,866365674	5,33E-05	0,001196628	down
ENSBTAG00000009634	*HOXC5*	protein-coding	0,94843252	5,53E-05	0,001235352	up
ENSBTAG00000016711	*PPIF*	protein-coding	0,735148389	5,53E-05	0,001235352	up
ENSBTAG00000011622	*C24H18orf21*	protein-coding	-0,660727449	5,62E-05	0,001245593	down
ENSBTAG00000049909	*AGBL1*	protein-coding	0,732403446	5,63E-05	0,001245892	up
ENSBTAG00000017752	*DYNLRB2*	protein-coding	-1,043102501	5,89E-05	0,001292582	down
ENSBTAG00000003341	*DOK2*	protein-coding	-0,917809229	6,01E-05	0,001315346	down
ENSBTAG00000032899	*NA*	NA	0,688624238	6,04E-05	0,00131726	up
ENSBTAG00000021173	*CCDC134*	protein-coding	0,644764973	6,13E-05	0,001328479	up
ENSBTAG00000054018	*NA*	NA	-1,084951598	6,15E-05	0,00133156	down
ENSBTAG00000010389	*STBD1*	protein-coding	0,562405253	6,26E-05	0,001347233	up
ENSBTAG00000013017	*GNAT2*	protein-coding	0,501778931	6,35E-05	0,001362738	up
ENSBTAG00000047621	*NA*	NA	-1,090195903	6,56E-05	0,001404714	down
ENSBTAG00000011766	*C7*	protein-coding	-0,598385352	6,81E-05	0,001447603	down
ENSBTAG00000000604	*GPNMB*	protein-coding	-1,600928143	6,86E-05	0,001455219	down
ENSBTAG00000038700	*FAM124B*	protein-coding	0,985017777	6,87E-05	0,001455219	up
ENSBTAG00000008592	*FCGR1A*	protein-coding	-1,129120105	6,87E-05	0,001455219	down
ENSBTAG00000008401	*PFKFB3*	protein-coding	-1,330827236	7,09E-05	0,001493902	down
ENSBTAG00000004361	*ZBTB37*	protein-coding	0,724395962	7,15E-05	0,00150507	up
ENSBTAG00000014555	*CASS4*	protein-coding	-1,234110525	7,24E-05	0,001521883	down
ENSBTAG00000009844	*CCN1*	protein-coding	-0,738894768	7,33E-05	0,001535925	down
ENSBTAG00000000917	*BMP1*	protein-coding	0,502717241	7,35E-05	0,001537569	up
ENSBTAG00000021263	*CYP4V2*	protein-coding	-0,53843528	7,46E-05	0,00155767	down
ENSBTAG00000004468	*BARX1*	protein-coding	0,974709146	7,55E-05	0,001573259	up
ENSBTAG00000029906	*MIR133A-2*	ncRNA	0,701854331	7,67E-05	0,001593039	up
ENSBTAG00000002024	*ASB18*	protein-coding	0,709015102	7,87E-05	0,001624298	up
ENSBTAG00000015326	*GSDME*	protein-coding	-0,613026318	7,96E-05	0,001635375	down
ENSBTAG00000039340	*SCN4B*	protein-coding	0,789257288	8,25E-05	0,001688114	up
ENSBTAG00000011238	*CD48*	protein-coding	-0,680092987	8,34E-05	0,001704436	down
ENSBTAG00000014548	*NIBAN1*	protein-coding	0,507633186	8,37E-05	0,001707174	up
ENSBTAG00000003650	*NR4A2*	protein-coding	-0,871856552	8,60E-05	0,001746713	down
ENSBTAG00000017187	*TMEM255A*	protein-coding	-0,783945237	8,68E-05	0,001755415	down
ENSBTAG00000050876	*NA*	NA	0,657708255	9,02E-05	0,001807046	up
ENSBTAG00000015821	*AMPD3*	protein-coding	-1,107446692	9,10E-05	0,001813257	down
ENSBTAG00000037778	*CXCL3*	protein-coding	-0,907228935	9,12E-05	0,001815674	down
ENSBTAG00000000246	*ME3*	protein-coding	-0,650599623	9,22E-05	0,001829933	down
ENSBTAG00000051012	*LOC104973829*	ncRNA	1,565281671	9,56E-05	0,001894173	up
ENSBTAG00000002965	*NEURL3*	protein-coding	-1,162100788	9,61E-05	0,001895084	down
ENSBTAG00000002574	*MYOZ2*	protein-coding	0,620543837	9,62E-05	0,001895084	up
ENSBTAG00000006260	*KIAA1614*	protein-coding	0,553681759	9,63E-05	0,001895084	up
ENSBTAG00000052788	*NA*	NA	0,845600929	9,64E-05	0,001895852	up
ENSBTAG00000015739	*MRC2*	protein-coding	0,556053933	9,71E-05	0,00190588	up
ENSBTAG00000019533	*ADTRP*	protein-coding	0,791054817	9,83E-05	0,001926836	up
ENSBTAG00000011241	*HPDL*	protein-coding	1,431119642	9,99E-05	0,001953133	up
ENSBTAG00000051072	*AQP4*	protein-coding	1,084635553	9,99E-05	0,001953133	up
ENSBTAG00000019378	*KIAA1755*	protein-coding	-0,694952515	0,000100965	0,001967985	down
ENSBTAG00000053677	*HERC1*	protein-coding	0,533814575	0,000102308	0,001989308	up
ENSBTAG00000015656	*WSCD1*	protein-coding	0,581874741	0,000102777	0,001993661	up
ENSBTAG00000008605	*DNAH2*	protein-coding	0,893891396	0,000103338	0,002001169	up
ENSBTAG00000021971	*SNCAIP*	protein-coding	0,581030412	0,000104243	0,002013252	up
ENSBTAG00000050626	*NA*	NA	0,752094475	0,0001082	0,00207702	up
ENSBTAG00000007823	*TG*	protein-coding	-0,752809107	0,000111005	0,002115295	down
ENSBTAG00000012815	*CLEC12A*	protein-coding	-1,136968262	0,000115142	0,002175485	down
ENSBTAG00000016768	*SCN3B*	protein-coding	-1,2874549	0,000118251	0,002226691	down
ENSBTAG00000000570	*RET*	protein-coding	-0,679687219	0,000118811	0,002234301	down

ENSBTAG00000050446	*NA*	NA	0,809465599	0,000125007	0,002329389	up
ENSBTAG00000006957	*SLC2A8*	protein-coding	-0,731933878	0,000127611	0,002365684	down
ENSBTAG00000020739	*NXT2*	protein-coding	-0,603723984	0,000130857	0,002413275	down
ENSBTAG00000051245	*SOX4*	protein-coding	0,819254421	0,000134191	0,002453058	up
ENSBTAG00000047827	*RENBP*	protein-coding	-0,861110017	0,000134386	0,002453058	down
ENSBTAG00000011644	*THSD4*	protein-coding	0,54432921	0,000136827	0,002493846	up
ENSBTAG00000021041	*PRAM1*	protein-coding	-0,783568547	0,000136969	0,002493846	down
ENSBTAG00000011545	*GDA*	protein-coding	-0,521004561	0,000142191	0,002572561	down
ENSBTAG00000015980	*FASN*	protein-coding	0,948491304	0,000144693	0,002607446	up
ENSBTAG00000021487	*CIART*	protein-coding	-0,843060244	0,000144848	0,002607446	down
ENSBTAG00000013235	*TINAGL1*	protein-coding	-0,557171758	0,000146431	0,00262933	down
ENSBTAG00000054349	*NA*	NA	0,512257414	0,000148091	0,00265247	up
ENSBTAG00000008583	*PALD1*	protein-coding	0,636614213	0,000149261	0,002670091	up
ENSBTAG00000013205	*IL1RAP*	protein-coding	-0,855130242	0,000150292	0,002685177	down
ENSBTAG00000004849	*IGSF3*	protein-coding	0,532158625	0,000153232	0,0027241	up
ENSBTAG00000052600	*NA*	NA	0,949373079	0,000154355	0,002737239	up
ENSBTAG00000011788	*CXHXorf38*	protein-coding	0,571055913	0,000156986	0,002777035	up
ENSBTAG00000048075	*IGSF23*	protein-coding	0,604409468	0,000158941	0,002797798	up
ENSBTAG00000011359	*CD7*	protein-coding	-1,026546469	0,000160767	0,002816087	down
ENSBTAG00000021249	*LAT*	protein-coding	-0,741572379	0,000162853	0,002845647	down
ENSBTAG00000001462	*FLT4*	protein-coding	0,848849247	0,000163443	0,002849574	up
ENSBTAG00000046121	*LOC100196897*	protein-coding	1,332685889	0,000163476	0,002849574	up
ENSBTAG00000049004	*NA*	NA	-0,743335673	0,000163816	0,002852036	down
ENSBTAG00000019567	*IL21R*	protein-coding	-0,980320598	0,000165634	0,002876679	down
ENSBTAG00000037951	*KLHL6*	protein-coding	0,970457075	0,000178568	0,003071491	up
ENSBTAG00000009287	*EBP*	protein-coding	0,578520366	0,00018646	0,003184275	up
ENSBTAG00000010661	*DPYSL3*	protein-coding	0,611530906	0,000187197	0,003189264	up
ENSBTAG00000033386	*TEX26*	protein-coding	-0,655458658	0,000189727	0,003220859	down
ENSBTAG00000048122	*CFD*	protein-coding	-0,608440541	0,000194456	0,003285553	down
ENSBTAG00000038523	*ATP1A4*	protein-coding	0,810750421	0,000196069	0,003305002	up
ENSBTAG00000009852	*NAV3*	protein-coding	0,573818515	0,000198707	0,003334527	up
ENSBTAG00000020105	*AQP7*	protein-coding	0,530253946	0,000201724	0,003368573	up
ENSBTAG00000018223	*CHI3L1*	protein-coding	-0,917319997	0,000203684	0,003385505	down
ENSBTAG00000043570	*NA*	NA	-0,536195101	0,000206212	0,003415619	down
ENSBTAG00000004034	*SESN3*	protein-coding	0,539310354	0,000211783	0,003479716	up
ENSBTAG00000001229	*SLC25A22*	protein-coding	-0,783504221	0,000212072	0,003480458	down
ENSBTAG00000020713	*BACH2*	protein-coding	-0,555144021	0,000213147	0,003490101	down
ENSBTAG00000011482	*SLC43A2*	protein-coding	-1,164934935	0,000213483	0,003491603	down
ENSBTAG00000038124	*FCGR2A*	protein-coding	-1,269715996	0,000213886	0,003494211	down
ENSBTAG00000052719	*NA*	NA	0,613094692	0,000215902	0,003519111	up
ENSBTAG00000038286	*PPDPFL*	protein-coding	-1,070351816	0,000216804	0,003529782	down
ENSBTAG00000020661	*ABCA1*	protein-coding	-0,851898937	0,00021877	0,003553716	down
ENSBTAG00000027051	*PTAFR*	protein-coding	-0,847132029	0,00022114	0,003580207	down
ENSBTAG00000005504	*TP53INP2*	protein-coding	0,722447057	0,000221793	0,003580881	up
ENSBTAG00000004207	*CD93*	protein-coding	0,591627415	0,000224717	0,003613394	up
ENSBTAG00000013420	*FSD1L*	protein-coding	0,617092266	0,000225784	0,003626479	up
ENSBTAG00000007103	*ITGAL*	protein-coding	-0,577742067	0,000228091	0,003655336	down
ENSBTAG00000007467	*MIB1*	protein-coding	0,513037021	0,000230398	0,003676274	up
ENSBTAG00000008353	*CDKN1A*	protein-coding	-0,786349012	0,00023471	0,003732156	down
ENSBTAG00000000595	*FBN3*	protein-coding	0,961442441	0,000239788	0,003796029	up
ENSBTAG00000006777	*TLCD1*	protein-coding	-0,7525428	0,000241023	0,003807153	down
ENSBTAG00000017032	*FREM2*	protein-coding	0,717883379	0,000245389	0,003863324	up
ENSBTAG00000008792	*RNASE6*	protein-coding	-0,791392606	0,000250223	0,003910331	down
ENSBTAG00000011960	*GOT1*	protein-coding	0,64443418	0,000250347	0,003910331	up
ENSBTAG00000046555	*WAS*	protein-coding	-0,642173169	0,000250854	0,003910331	down
ENSBTAG00000014218	*DKK1*	protein-coding	-0,90966488	0,000260592	0,004044833	down
ENSBTAG00000000745	*AQP1*	protein-coding	0,665543237	0,000262488	0,004069842	up
ENSBTAG00000015032	*CD14*	protein-coding	-0,781273708	0,000264472	0,004091741	down
ENSBTAG00000003718	*HACL1*	protein-coding	-0,622107995	0,000266097	0,004112437	down
ENSBTAG00000015144	*GMIP*	protein-coding	-0,516671256	0,000270537	0,004167556	down
ENSBTAG00000005617	*SLC41A3*	protein-coding	0,652927848	0,000271128	0,004172161	up
ENSBTAG00000025398	*LOC504548*	protein-coding	-0,703383915	0,000272114	0,00417835	down
ENSBTAG00000019332	*SLC9C1*	protein-coding	0,733519344	0,000275862	0,004219376	up
ENSBTAG00000019053	*MAP9*	protein-coding	0,669767976	0,000282499	0,004287214	up
ENSBTAG00000014418	*FHIT*	protein-coding	-0,760439804	0,000284876	0,004309582	down

ENSBTAG00000023851	*FAM102A*	protein-coding	0,730332129	0,000290929	0,004382623	up
ENSBTAG00000019302	*BCL2*	protein-coding	-0,546952046	0,000293218	0,004393967	down
ENSBTAG00000012673	*CDK18*	protein-coding	0,630655464	0,000296651	0,004431499	up
ENSBTAG00000003088	*LANCL3*	protein-coding	0,755769486	0,000303647	0,004517147	up
ENSBTAG00000002025	*AMIGO2*	protein-coding	0,79596603	0,000304764	0,004529055	up
ENSBTAG00000008798	*DRC3*	protein-coding	0,805489061	0,000315035	0,004652702	up
ENSBTAG00000007379	*ALK*	protein-coding	-0,814871593	0,000317347	0,004663538	down
ENSBTAG00000007835	*GPT*	protein-coding	0,584100485	0,000317398	0,004663538	up
ENSBTAG00000055014	*SH3BGRL2*	protein-coding	-0,69925325	0,000319978	0,004691814	down
ENSBTAG00000012599	*SMAD3*	protein-coding	0,520779857	0,000325581	0,004750078	up
ENSBTAG00000012128	*AASS*	protein-coding	0,517824837	0,000328433	0,004773041	up
ENSBTAG00000009137	*NKG7*	protein-coding	-0,8369686	0,000329086	0,004773041	down
ENSBTAG00000023929	*FOSL2*	protein-coding	-0,650871533	0,000332847	0,004821235	down
ENSBTAG00000044348	*MIR2466*	ncRNA	0,555969209	0,00033325	0,004822183	up
ENSBTAG00000011266	*ZBTB16*	protein-coding	-0,520406787	0,000336051	0,004852906	down
ENSBTAG00000005431	*LMCD1*	protein-coding	0,59952641	0,000338727	0,004886619	up
ENSBTAG00000004950	*BRB*	protein-coding	-0,805253515	0,000340089	0,004901321	down
ENSBTAG00000033308	*NA*	NA	0,535012187	0,000342456	0,004925519	up
ENSBTAG00000005300	*TMEM51*	protein-coding	0,650772242	0,000344671	0,004939448	up
ENSBTAG00000012302	*RTN4RL1*	protein-coding	-0,613813813	0,000347488	0,004972898	down
ENSBTAG00000007094	*MINAR1*	protein-coding	0,929519724	0,000362784	0,00514549	up
ENSBTAG00000048796	*NA*	NA	0,573793782	0,000365393	0,005163356	up
ENSBTAG00000031777	*GDPD5*	protein-coding	-0,687830348	0,000366449	0,005171841	down
ENSBTAG00000038464	*PLIN5*	protein-coding	-0,598889821	0,000370414	0,00522266	down
ENSBTAG00000007415	*SLC7A8*	protein-coding	-1,107931319	0,000378517	0,005323321	down
ENSBTAG00000005284	*SERPINE3*	protein-coding	-0,541627277	0,000378799	0,005323321	down
ENSBTAG00000051059	*NA*	NA	0,682518309	0,000380378	0,005327313	up
ENSBTAG00000015160	*PLD4*	protein-coding	-0,762901318	0,000387699	0,005384319	down
ENSBTAG00000049281	*NA*	NA	0,714064514	0,000387824	0,005384319	up
ENSBTAG00000038938	*LOC781710*	protein-coding	-0,771306673	0,0003879	0,005384319	down
ENSBTAG00000052348	*NA*	NA	0,586360666	0,000390124	0,00539842	up
ENSBTAG00000049549	*NA*	NA	0,651646067	0,000391485	0,005407859	up
ENSBTAG00000000897	*IQGAP2*	protein-coding	-0,514686114	0,000394494	0,005426359	down
ENSBTAG00000005668	*SLC39A8*	protein-coding	-0,76735073	0,000395053	0,005426359	down
ENSBTAG00000013929	*RRAD*	protein-coding	0,726955568	0,000395099	0,005426359	up
ENSBTAG00000002333	*HOPX*	protein-coding	-0,638762561	0,000398681	0,005459831	down
ENSBTAG00000033335	*LOC617694*	pseudo	-1,745095483	0,000410781	0,005598746	down
ENSBTAG00000051075	*NA*	NA	0,628567256	0,000411603	0,005601072	up
ENSBTAG00000018424	*ACKR3*	protein-coding	0,54412314	0,000411734	0,005601072	up
ENSBTAG00000029826	*MIR133B*	ncRNA	0,748754392	0,000415035	0,005624582	up
ENSBTAG00000010981	*CIB2*	protein-coding	1,013645214	0,000418106	0,005660843	up
ENSBTAG00000049218	*NA*	NA	0,60781296	0,000421166	0,005696881	up
ENSBTAG00000016312	*LGALS4*	protein-coding	-0,514374789	0,00042282	0,005708466	down
ENSBTAG00000008509	*SLC38A3*	protein-coding	0,751319469	0,000439636	0,005891043	up
ENSBTAG00000017690	*CARNS1*	protein-coding	0,805037598	0,000441482	0,005910243	up
ENSBTAG00000046324	*LOC100297676*	protein-coding	-0,588267654	0,000443624	0,005929964	down
ENSBTAG00000012393	*AGT*	protein-coding	-0,939446673	0,000446692	0,005952153	down
ENSBTAG00000018571	*IL1RL1*	protein-coding	-1,440652266	0,000453508	0,006031743	down
ENSBTAG00000053497	*GNLY*	protein-coding	-0,707687697	0,000454221	0,006035612	down
ENSBTAG00000012409	*POSTN*	protein-coding	-1,134814026	0,000460981	0,006099559	down
ENSBTAG00000014237	*CYTH4*	protein-coding	-0,660986366	0,000466583	0,006137194	down
ENSBTAG00000011992	*PTH2R*	protein-coding	0,830375822	0,000474774	0,006193696	up
ENSBTAG00000047997	*PRPS2*	protein-coding	-0,534925449	0,000487722	0,006333957	down
ENSBTAG00000005461	*SOX15*	protein-coding	-0,519055532	0,000488574	0,00633906	down
ENSBTAG00000052721	*NA*	NA	-0,897062609	0,000492299	0,006375831	down
ENSBTAG00000047902	*ULBP21*	protein-coding	1,036069758	0,00049391	0,006390907	up
ENSBTAG00000051385	*NA*	NA	-0,814151564	0,00049942	0,00643892	down
ENSBTAG00000054325	*NA*	NA	-0,640317917	0,000501588	0,006455237	down
ENSBTAG00000005170	*ADGRG5*	protein-coding	-0,830548339	0,000515066	0,006587228	down
ENSBTAG00000007828	*SLA*	protein-coding	-0,70130839	0,000523988	0,006671529	down
ENSBTAG00000004705	*ILDR1*	protein-coding	-0,809685655	0,000528079	0,006711672	down
ENSBTAG00000021298	*NTMT2*	protein-coding	0,903396551	0,00053665	0,006802474	up
ENSBTAG00000026638	*CATSPER4*	protein-coding	0,578618527	0,000539385	0,0068311	up
ENSBTAG00000009830	*PLEKHB1*	protein-coding	0,577286588	0,000548985	0,006922048	up
ENSBTAG00000051933	*NA*	NA	-1,035396089	0,000558479	0,007017022	down

ENSBTAG00000001835	*GJA1*	protein-coding	0,645019263	0,000559443	0,007019575	up
ENSBTAG00000024503	*FCER1G*	protein-coding	-0,562935136	0,000560227	0,007020504	down
ENSBTAG00000009775	*PRRG3*	protein-coding	0,812375706	0,000571469	0,007136383	up
ENSBTAG00000016650	*TIGAR*	protein-coding	0,54623734	0,000585461	0,007273037	up
ENSBTAG00000024340	*TRIL*	protein-coding	0,719992367	0,000591212	0,007331745	up
ENSBTAG00000054043	*NA*	NA	0,911562617	0,00059291	0,007333734	up
ENSBTAG00000005708	*KIF20B*	protein-coding	-0,510550665	0,000612904	0,007528984	down
ENSBTAG00000023452	*B9D2*	protein-coding	0,502584808	0,000624492	0,007638547	up
ENSBTAG00000020872	*C5AR1*	protein-coding	-0,973474663	0,000629661	0,007683412	down
ENSBTAG00000019605	*OPA3*	protein-coding	0,607498592	0,000637134	0,007725239	up
ENSBTAG00000001852	*BREH1*	protein-coding	-0,602963961	0,000637191	0,007725239	down
ENSBTAG00000049048	*NA*	NA	0,70643523	0,000637517	0,007725239	up
ENSBTAG00000029926	*MIR1-2*	ncRNA	0,739482044	0,000639717	0,007739496	up
ENSBTAG00000001788	*PDPN*	protein-coding	-0,626602161	0,000639775	0,007739496	down
ENSBTAG00000015419	*ARHGEF37*	protein-coding	0,526764194	0,000640794	0,00774447	up
ENSBTAG00000006686	*NPNT*	protein-coding	0,697017538	0,00064154	0,00774447	up
ENSBTAG00000036363	*MIR133A-1*	ncRNA	0,753816155	0,000646726	0,007764504	up
ENSBTAG00000007262	*ZNF536*	protein-coding	0,648813199	0,000647801	0,007770898	up
ENSBTAG00000005215	*IL10RA*	protein-coding	-0,837224324	0,000649047	0,007779327	down
ENSBTAG00000006801	*TMEM106A*	protein-coding	-0,563441565	0,000651225	0,007792374	down
ENSBTAG00000034091	*LRRC72*	protein-coding	0,585103286	0,000652044	0,007795657	up
ENSBTAG00000034519	*SMIM29*	protein-coding	-0,514984856	0,000656823	0,00782664	down
ENSBTAG00000019002	*SLC2A12*	protein-coding	0,502038853	0,000657883	0,007832744	up
ENSBTAG00000020355	*KLF4*	protein-coding	-0,569426782	0,000659603	0,007846704	down
ENSBTAG00000045912	*NA*	NA	-0,534391017	0,00066077	0,007854055	down
ENSBTAG00000048565	*BASP1*	protein-coding	-0,576470599	0,000665869	0,007901515	down
ENSBTAG00000020644	*GPC4*	protein-coding	0,583124724	0,000666419	0,007901515	up
ENSBTAG00000019181	*BAIAP2L1*	protein-coding	-0,608573908	0,000671508	0,007935554	down
ENSBTAG00000007689	*LPIN1*	protein-coding	-0,612072227	0,000673962	0,007953253	down
ENSBTAG00000027205	*PGBD5*	protein-coding	-0,996656294	0,000675218	0,007959675	down
ENSBTAG00000001992	*CYP51A1*	protein-coding	0,515842461	0,00067673	0,007970928	up
ENSBTAG00000010955	*NUP54*	protein-coding	-0,508112173	0,000686013	0,008042797	down
ENSBTAG00000009816	*SCX*	protein-coding	0,88464989	0,00068908	0,00806331	up
ENSBTAG00000008192	*PLPPR2*	protein-coding	0,694449762	0,000697409	0,008154099	up
ENSBTAG00000006688	*RPS6KL1*	protein-coding	0,539625893	0,000699366	0,008168529	up
ENSBTAG00000051104	*CGREF1*	protein-coding	-0,735292778	0,000702305	0,008184797	down
ENSBTAG00000021029	*PRR5L*	protein-coding	-0,857697587	0,00071414	0,008255292	down
ENSBTAG00000014551	*TNFAIP8*	protein-coding	-0,573316951	0,000715396	0,008257661	down
ENSBTAG00000033304	*TCIM*	protein-coding	-0,583484468	0,000715499	0,008257661	down
ENSBTAG00000021202	*ACOT7*	protein-coding	-0,72381563	0,000723008	0,008330897	down
ENSBTAG00000013152	*NIPSNAP1*	protein-coding	0,563513692	0,000727332	0,008368342	up
ENSBTAG00000009850	*GIMAP7*	protein-coding	-0,666261516	0,000730102	0,008385629	down
ENSBTAG00000034182	*LOC782966*	protein-coding	0,515438975	0,000734354	0,008427698	up
ENSBTAG00000031573	*NMRK2*	protein-coding	0,757227837	0,00074531	0,008512473	up
ENSBTAG00000023398	*UBASH3A*	protein-coding	-0,837844755	0,000761259	0,0086532	down
ENSBTAG00000027516	*APLNR*	protein-coding	0,560397843	0,000764883	0,00867373	up
ENSBTAG00000019988	*GNA15*	protein-coding	-0,792738415	0,000767894	0,008700969	down
ENSBTAG00000016185	*ENAH*	protein-coding	0,587716393	0,000780446	0,008829217	up
ENSBTAG00000049422	*TIFA*	protein-coding	-0,594338044	0,000800325	0,009004286	down
ENSBTAG00000021481	*CA14*	protein-coding	0,521769881	0,000806083	0,009054829	up
ENSBTAG00000044105	*FOXO1*	protein-coding	-0,889390383	0,0008324	0,009284861	down
ENSBTAG00000000133	*CD68*	protein-coding	-0,609691677	0,000840558	0,009339456	down
ENSBTAG00000051562	*NA*	NA	-0,833080405	0,000857788	0,009472069	down
ENSBTAG00000000987	*OTOG*	protein-coding	0,736836488	0,000859803	0,009479694	up
ENSBTAG00000000983	*ABCC8*	protein-coding	0,540686963	0,000868301	0,009558655	up
ENSBTAG00000018146	*VAT1L*	protein-coding	0,511800286	0,000871339	0,009580431	up
ENSBTAG00000054745	*NA*	NA	0,646558965	0,00087796	0,009637566	up
ENSBTAG00000019347	*PLXDC1*	protein-coding	0,564291897	0,000879989	0,009646975	up
ENSBTAG00000014127	*PTGS2*	protein-coding	-0,970362163	0,000882045	0,009657949	down
ENSBTAG00000053294	*NA*	NA	0,594010038	0,000886733	0,009701862	up
ENSBTAG00000003408	*NA*	NA	-1,105423695	0,000888709	0,009708634	down
ENSBTAG00000006937	*NA*	NA	-0,524499864	0,000891947	0,009736578	down
ENSBTAG00000043962	*SLC2A13*	protein-coding	-0,875803013	0,000894695	0,00975913	down
ENSBTAG00000054498	*NA*	NA	0,58777497	0,000896506	0,009771432	up
ENSBTAG00000013118	*CD86*	protein-coding	-0,649424808	0,000897613	0,009776053	down

ENSBTAG00000002605	*TRIM16*	protein-coding	0,501864035	0,000898805	0,009781592	up
ENSBTAG00000039652	*PTGDR2*	protein-coding	-0,563439595	0,000914646	0,009890881	down
ENSBTAG00000026637	*CCDC162P*	pseudo	0,559101267	0,000924472	0,009950306	up
ENSBTAG00000051767	*NA*	NA	0,508113692	0,000924986	0,009950306	up
ENSBTAG00000016688	*NA*	NA	0,524205429	0,000927444	0,009964456	up
ENSBTAG00000020087	*CAMK2A*	protein-coding	0,587653435	0,000961863	0,010251235	up
ENSBTAG00000021709	*SPI1*	protein-coding	-0,663911695	0,000962016	0,010251235	down
ENSBTAG00000014172	*FGD2*	protein-coding	-0,608726726	0,000969349	0,010314113	down
ENSBTAG00000031658	*SEMA6B*	protein-coding	0,515046673	0,000969358	0,010314113	up
ENSBTAG00000012991	*NA*	NA	0,899544472	0,000981552	0,010436102	up
ENSBTAG00000011896	*GRIP2*	protein-coding	0,529439809	0,001000316	0,010569899	up
ENSBTAG00000045925	*CITED1*	protein-coding	-0,625933744	0,001000784	0,010569899	down
ENSBTAG00000038584	*OLFM1*	protein-coding	0,914620488	0,00101553	0,010686212	up
ENSBTAG00000020747	*ASS1*	protein-coding	-0,590263039	0,001019908	0,01071652	down
ENSBTAG00000007827	*RCOR2*	protein-coding	0,725954313	0,001021057	0,010720723	up
ENSBTAG00000013476	*CPA5*	protein-coding	0,556559048	0,001022367	0,010726609	up
ENSBTAG00000037756	*TET1*	protein-coding	0,585391767	0,001054566	0,010960006	up
ENSBTAG00000003016	*PTPN7*	protein-coding	-0,790437754	0,001054568	0,010960006	down
ENSBTAG00000018948	*HIF3A*	protein-coding	-0,755855544	0,001064336	0,0110451	down
ENSBTAG00000011987	*ERG28*	protein-coding	0,538601601	0,001075344	0,01110335	up
ENSBTAG00000049736	*NA*	NA	0,75346933	0,001107695	0,011388096	up
ENSBTAG00000012338	*FAH*	protein-coding	-0,716805421	0,001118698	0,011476481	down
ENSBTAG00000034645	*PON3*	protein-coding	-0,519064721	0,001153115	0,011718005	down
ENSBTAG00000002151	*ASB15*	protein-coding	0,560344744	0,001163735	0,011769757	up
ENSBTAG00000012534	*BEND3*	protein-coding	0,555158746	0,001192954	0,012005887	up
ENSBTAG00000019497	*ILDR2*	protein-coding	0,590484097	0,001197179	0,012039936	up
ENSBTAG00000045579	*LOC100295750*	protein-coding	-0,605632279	0,00120572	0,012091842	down
ENSBTAG00000039050	*P2RY2*	protein-coding	0,536044731	0,001213966	0,012157495	up
ENSBTAG00000010954	*ART3*	protein-coding	-0,659508975	0,001217594	0,012168286	down
ENSBTAG00000010158	*NA*	NA	0,560887651	0,001242461	0,012382215	up
ENSBTAG00000008271	*MEDAG*	protein-coding	-0,555835345	0,001254446	0,012473816	down
ENSBTAG00000054310	*NA*	NA	0,522776706	0,001255139	0,012473816	up
ENSBTAG00000000181	*SUSD3*	protein-coding	-0,60636259	0,00127171	0,012603489	down
ENSBTAG00000012285	*ESRRB*	protein-coding	0,578603881	0,001275815	0,012621997	up
ENSBTAG00000003749	*KCNMB4*	protein-coding	-0,55109195	0,001277622	0,01262711	down
ENSBTAG00000048956	*NA*	NA	0,62664434	0,001278919	0,012631197	up
ENSBTAG00000019741	*C3AR1*	protein-coding	-1,004569602	0,001287235	0,012669613	down
ENSBTAG00000017060	*ITGB2*	protein-coding	-0,515841422	0,001294924	0,012727782	down
ENSBTAG00000010042	*RIOK3*	protein-coding	-0,501748986	0,001303531	0,012786041	down
ENSBTAG00000021189	*ARHGAP36*	protein-coding	0,996475848	0,001308344	0,012824456	up
ENSBTAG00000020984	*RAPGEF4*	protein-coding	-0,520270528	0,001309246	0,012824519	down
ENSBTAG00000015060	*GBP5*	protein-coding	-0,518330801	0,001312605	0,012848625	down
ENSBTAG00000009587	*NRG3*	protein-coding	-0,548755724	0,001319781	0,012892413	down
ENSBTAG00000052475	*NA*	NA	0,882941999	0,001326309	0,012947345	up
ENSBTAG00000048740	*LOC518495*	protein-coding	0,515032753	0,001332515	0,012999057	up
ENSBTAG00000018724	*TMEM82*	protein-coding	-0,900100501	0,001336869	0,013032653	down
ENSBTAG00000005812	*TP73*	protein-coding	-0,682734746	0,001351314	0,01313767	down
ENSBTAG00000011178	*NA*	NA	-0,659262517	0,001366848	0,013270666	down
ENSBTAG00000021144	*PSTPIP1*	protein-coding	-0,570044389	0,001373887	0,013314633	down
ENSBTAG00000014328	*MGC138914*	protein-coding	-0,625317081	0,001378503	0,013347606	down
ENSBTAG00000051330	*WC-7*	protein-coding	-1,15249335	0,001385483	0,013389845	down
ENSBTAG00000008464	*ABCB9*	protein-coding	0,746643108	0,001395297	0,013464662	up
ENSBTAG00000000144	*LOC100335205*	protein-coding	-0,615925791	0,001456695	0,013887921	down
ENSBTAG00000001094	*MTUS2*	protein-coding	0,719128205	0,001457576	0,013887921	up
ENSBTAG00000007101	*F3*	protein-coding	-0,980505682	0,001474288	0,013991072	down
ENSBTAG00000021313	*RUNDC3B*	protein-coding	-0,691692406	0,001511518	0,014271909	down
ENSBTAG00000002947	*LY9*	protein-coding	-0,56666201	0,001527868	0,014366176	down
ENSBTAG00000031814	*SDS*	protein-coding	0,64022676	0,001542459	0,014470957	up
ENSBTAG00000008057	*PARVG*	protein-coding	-0,654802851	0,001544752	0,014473209	down
ENSBTAG00000044087	*NA*	NA	0,610973872	0,001545319	0,014473209	up
ENSBTAG00000006800	*FGF6*	protein-coding	1,011250919	0,001590482	0,014818107	up
ENSBTAG00000005857	*SLC6A1*	protein-coding	0,65316213	0,001597119	0,014852134	up
ENSBTAG00000043951	*IMPA2*	protein-coding	-0,609641839	0,001623712	0,015055157	down
ENSBTAG00000049266	*NA*	NA	-0,609135548	0,001625548	0,015055157	down
ENSBTAG00000051359	*NA*	NA	0,86930878	0,00162639	0,015055157	up

ENSBTAG00000048660	*C19H17orf113*	protein-coding	-0,855376032	0,001671812	0,015371847	down
ENSBTAG00000012695	*LCK*	protein-coding	-0,516544969	0,001722966	0,01578577	down
ENSBTAG00000017370	*ARFGEF3*	protein-coding	0,522144208	0,001751311	0,015984068	up
ENSBTAG00000020854	*BCL6B*	protein-coding	0,502164136	0,001768073	0,016085752	up
ENSBTAG00000008827	*SPOCK2*	protein-coding	-0,906160378	0,001772146	0,01610234	down
ENSBTAG00000019686	*NCKAP1L*	protein-coding	-0,594141106	0,001804975	0,016307444	down
ENSBTAG00000018235	*SLC45A2*	protein-coding	0,707237994	0,001807054	0,016315927	up
ENSBTAG00000004206	*LRRC55*	protein-coding	0,852730016	0,001832035	0,016468754	up
ENSBTAG00000050193	*NA*	NA	-1,041272392	0,00186938	0,016699561	down
ENSBTAG00000031214	*LOC618409*	protein-coding	-0,561217356	0,001880253	0,016755228	down
ENSBTAG00000009381	*LCP2*	protein-coding	-0,56736198	0,001880295	0,016755228	down
ENSBTAG00000039722	*LOC782061*	protein-coding	0,966477074	0,001901278	0,016910622	up
ENSBTAG00000015649	*TMEM156*	protein-coding	-0,581817536	0,001924341	0,017062728	down
ENSBTAG00000009405	*TRPC4*	protein-coding	-0,590794568	0,0019389	0,017159921	down
ENSBTAG00000010408	*IKBKE*	protein-coding	-0,751906259	0,001958234	0,017298939	down
ENSBTAG00000052744	*C18H16orf74*	protein-coding	0,610471806	0,001967883	0,017352046	up
ENSBTAG00000011120	*NA*	NA	0,592983994	0,001969676	0,017357163	up
ENSBTAG00000013918	*ADGRL3*	protein-coding	0,679398553	0,001999671	0,017578185	up
ENSBTAG00000006963	*RPL12*	protein-coding	-0,675841171	0,002057716	0,018010992	down
ENSBTAG00000004727	*PAQR4*	protein-coding	0,5087773	0,002065515	0,018055138	up
ENSBTAG00000016444	*RETREG1*	protein-coding	0,693842131	0,002079166	0,018143263	up
ENSBTAG00000001235	*CD200R1L*	protein-coding	-0,878846689	0,002128569	0,018450602	down
ENSBTAG00000030340	*IFFO2*	protein-coding	0,510326826	0,002196074	0,018909759	up
ENSBTAG00000053361	*KLHL30*	protein-coding	-0,644951372	0,002217504	0,019059877	down
ENSBTAG00000019137	*SCN7A*	protein-coding	-0,986684552	0,002256194	0,019288166	down
ENSBTAG00000007397	*FOLH1B*	protein-coding	0,525042629	0,002271518	0,019396001	up
ENSBTAG00000013191	*AGRN*	protein-coding	0,512493714	0,002288889	0,019509405	up
ENSBTAG00000050237	*NA*	NA	-0,766681531	0,002299435	0,01957794	down
ENSBTAG00000002009	*NA*	NA	-0,766682805	0,002299666	0,01957794	down
ENSBTAG00000020590	*FZD2*	protein-coding	0,535479095	0,002342617	0,019860892	up
ENSBTAG00000027397	*PPP1R3D*	protein-coding	0,625784593	0,002368617	0,020010201	up
ENSBTAG00000051340	*NA*	NA	-0,978200599	0,002398125	0,020148319	down
ENSBTAG00000007325	*TPSB2*	protein-coding	-0,814263355	0,002403269	0,020148319	down
ENSBTAG00000014470	*ALDH1L1*	protein-coding	0,911126317	0,002412581	0,020190905	up
ENSBTAG00000045854	*LOC100852077*	protein-coding	-0,851989208	0,002445214	0,020380583	down
ENSBTAG00000016998	*ARL11*	protein-coding	0,653672155	0,002486718	0,020582662	up
ENSBTAG00000052038	*NA*	NA	-1,14153032	0,002517369	0,020740395	down
ENSBTAG00000034609	*NA*	NA	-0,741494864	0,002555151	0,02095517	down
ENSBTAG00000013439	*ARHGEF26*	protein-coding	0,731578389	0,002557551	0,020962836	up
ENSBTAG00000020198	*METRN*	protein-coding	0,638605892	0,002567581	0,021008947	up
ENSBTAG00000053836	*CCDC71L*	protein-coding	-0,925541119	0,002570931	0,021013584	down
ENSBTAG00000045767	*LAT2*	protein-coding	-0,531644233	0,002600827	0,021182725	down
ENSBTAG00000004908	*CHRNE*	protein-coding	0,611816094	0,00260214	0,021182725	up
ENSBTAG00000000868	*ZFP1*	protein-coding	-0,585993589	0,002613872	0,021266134	down
ENSBTAG00000047743	*KCNIP4*	protein-coding	-1,932533945	0,002617237	0,02128141	down
ENSBTAG00000020028	*NA*	NA	0,553182066	0,002624834	0,02130686	up
ENSBTAG00000055017	*SCIMP*	protein-coding	-0,822712144	0,002664177	0,021540702	down
ENSBTAG00000050510	*LOC112442215*	protein-coding	-0,874745593	0,002825181	0,022474244	down
ENSBTAG00000014897	*UNC5A*	protein-coding	0,638853603	0,002887032	0,022877252	up
ENSBTAG00000015290	*BAIAP2L2*	protein-coding	-0,630046248	0,002893262	0,022889507	down
ENSBTAG00000026437	*NA*	NA	1,274235377	0,003008947	0,023556696	up
ENSBTAG00000008807	*FBXL22*	protein-coding	0,545325373	0,003008989	0,023556696	up
ENSBTAG00000050462	*NA*	NA	0,63687306	0,003032597	0,023689684	up
ENSBTAG00000012444	*ADAM12*	protein-coding	0,779536308	0,003041187	0,023743821	up
ENSBTAG00000046257	*GIMAP4*	protein-coding	-0,626959863	0,003061108	0,023849395	down
ENSBTAG00000008441	*SOCS3*	protein-coding	-0,590448402	0,003112396	0,024128656	down
ENSBTAG00000021835	*PRICKLE2*	protein-coding	0,548629295	0,003147195	0,024319386	up
ENSBTAG00000054842	*MIR2285AH*	ncRNA	0,597760091	0,003158282	0,024347586	up
ENSBTAG00000014090	*ARID5A*	protein-coding	-0,535788253	0,003170036	0,024382318	down
ENSBTAG00000018366	*SLC15A3*	protein-coding	-0,528054644	0,003219523	0,024626309	down
ENSBTAG00000011602	*RASAL3*	protein-coding	-0,598428611	0,003253143	0,024750519	down
ENSBTAG00000053478	*LOC112449602*	protein-coding	-0,524752566	0,003323384	0,025137918	down
ENSBTAG00000051967	*LOC112443014*	ncRNA	0,858364319	0,003353798	0,025314452	up
ENSBTAG00000053974	*NA*	NA	0,543562649	0,003358964	0,025340078	up
ENSBTAG00000016520	*LOC786948*	protein-coding	-0,700127588	0,003401227	0,025497444	down

ENSBTAG00000021329	*ZBTB3*	protein-coding	0,64097296	0,003446537	0,025716054	up
ENSBTAG00000009489	*CACNA2D2*	protein-coding	0,620822237	0,003509319	0,026021695	up
ENSBTAG00000015991	*CDH1*	protein-coding	-0,949164195	0,00351957	0,026070687	down
ENSBTAG00000047531	*ARHGAP4*	protein-coding	-0,536218136	0,003548549	0,026203967	down
ENSBTAG00000013103	*COL1A1*	protein-coding	0,531490348	0,003566617	0,026294778	up
ENSBTAG00000051401	*NA*	NA	-0,510732052	0,003571569	0,026295351	down
ENSBTAG00000000243	*HEBP2*	protein-coding	0,769299978	0,003603585	0,026433063	up
ENSBTAG00000047225	*BCL2L14*	protein-coding	-0,904521657	0,003613149	0,026454158	down
ENSBTAG00000013736	*PROM1*	protein-coding	-0,801084225	0,003640065	0,026605193	down
ENSBTAG00000050953	*LOC515828*	protein-coding	-0,614111818	0,003656052	0,026653976	down
ENSBTAG00000020620	*RGS2*	protein-coding	-0,628296471	0,003662305	0,026672393	down
ENSBTAG00000018016	*NUPR1*	protein-coding	-0,640861825	0,00367935	0,026773346	down
ENSBTAG00000014051	*ANGPT1*	protein-coding	0,554026453	0,00384012	0,027699576	up
ENSBTAG00000002623	*SAMSN1*	protein-coding	-0,656595541	0,00384882	0,027730577	down
ENSBTAG00000011390	*CHRND*	protein-coding	-0,72991247	0,003850231	0,027730577	down
ENSBTAG00000003845	*CSRNP1*	protein-coding	-0,577284511	0,003931291	0,028149419	down
ENSBTAG00000027204	*LOC100300510*	protein-coding	-0,621223678	0,003951516	0,028275074	down
ENSBTAG00000049718	*NA*	NA	0,539540202	0,003957904	0,028292488	up
ENSBTAG00000051333	*ARID5B*	protein-coding	-0,71686222	0,003995158	0,028416854	down
ENSBTAG00000040590	*NA*	NA	0,556155228	0,004019179	0,02855932	up
ENSBTAG00000047078	*NTF4*	protein-coding	0,57089343	0,004094521	0,028950936	up
ENSBTAG00000053653	*NA*	NA	1,060370696	0,004117229	0,029032452	up
ENSBTAG00000017733	*CA2*	protein-coding	0,570905007	0,004118222	0,029032452	up
ENSBTAG00000015582	*HMOX1*	protein-coding	-0,636181226	0,004127368	0,029068291	down
ENSBTAG00000017411	*AK5*	protein-coding	-0,866304049	0,004182327	0,029426394	down
ENSBTAG00000050861	*NA*	NA	0,560253944	0,004250035	0,029740203	up
ENSBTAG00000006563	*KLHL40*	protein-coding	-0,54250597	0,004388993	0,030510657	down
ENSBTAG00000050417	*LOC788334*	protein-coding	0,517106054	0,004393723	0,030510657	up
ENSBTAG00000055197	*NA*	NA	-0,585088516	0,00439449	0,030510657	down
ENSBTAG00000049469	*MIR12003*	ncRNA	-0,864353864	0,004402912	0,030510657	down
ENSBTAG00000010866	*BMPER*	protein-coding	0,757938933	0,004405419	0,030510657	up
ENSBTAG00000012896	*METTL7B*	protein-coding	-0,952887412	0,004425433	0,030551071	down
ENSBTAG00000007921	*DAPP1*	protein-coding	-0,538960465	0,004440187	0,030625168	down
ENSBTAG00000039686	*NA*	NA	0,667691884	0,004547939	0,031216097	up
ENSBTAG00000019076	*PAK6*	protein-coding	0,820642215	0,004631675	0,031654202	up
ENSBTAG00000013125	*PLAUR*	protein-coding	-0,794559423	0,004817971	0,032615885	down
ENSBTAG00000006762	*SH3RF2*	protein-coding	-0,598599732	0,004832244	0,032661568	down
ENSBTAG00000043580	*NA*	NA	0,680165587	0,004844003	0,032699304	up
ENSBTAG00000001801	*SYT4*	protein-coding	-0,558429133	0,004937702	0,033183179	down
ENSBTAG00000031809	*DENND1C*	protein-coding	-0,541566199	0,004959928	0,033293401	down
ENSBTAG00000039922	*ARAP2*	protein-coding	-0,561207333	0,004966892	0,033308899	down
ENSBTAG00000022449	*SCD5*	protein-coding	0,581697781	0,005001597	0,033463235	up
ENSBTAG00000008004	*NCF2*	protein-coding	-0,705132865	0,005029431	0,033570982	down
ENSBTAG00000027563	*ALX4*	protein-coding	0,660252814	0,00505732	0,033650822	up
ENSBTAG00000009656	*BOLA-DQA2*	protein-coding	-0,554490521	0,005066011	0,033650822	down
ENSBTAG00000007253	*TSPAN33*	protein-coding	-0,504557503	0,005072896	0,033672641	down
ENSBTAG00000017020	*S100G*	protein-coding	-1,519065397	0,005091428	0,033748689	down
ENSBTAG00000002647	*RGS10*	protein-coding	-0,545452225	0,005198166	0,034297354	down
ENSBTAG00000046900	*GGT1*	protein-coding	0,545457313	0,005230457	0,03442783	up
ENSBTAG00000012526	*APBB1IP*	protein-coding	-0,507551668	0,005232376	0,03442783	down
ENSBTAG00000044073	*CD248*	protein-coding	0,506222989	0,005273211	0,034609505	up
ENSBTAG00000021272	*ABCG1*	protein-coding	-0,703060295	0,005406739	0,035218864	down
ENSBTAG00000001034	*IL18R1*	protein-coding	-0,850523784	0,005477744	0,03553581	down
ENSBTAG00000014536	*SFXN1*	protein-coding	-0,70243733	0,005490549	0,035602741	down
ENSBTAG00000008597	*SLC45A4*	protein-coding	0,562639322	0,005773139	0,037032451	up
ENSBTAG00000019960	*TM4SF18*	protein-coding	0,685423489	0,005806212	0,037118528	up
ENSBTAG00000020745	*HIVEP3*	protein-coding	0,597220447	0,005867707	0,037404348	up
ENSBTAG00000012349	*LAX1*	protein-coding	-0,629454001	0,005928703	0,037654155	down
ENSBTAG00000009907	*MAPK4*	protein-coding	0,575161218	0,005992927	0,037960426	up
ENSBTAG00000010732	*MMP23*	protein-coding	-0,603822649	0,006002686	0,037960426	down
ENSBTAG00000020147	*BBS1*	protein-coding	0,506456416	0,006066651	0,038179349	up
ENSBTAG00000005078	*UCHL1*	protein-coding	-0,858160421	0,006082728	0,038240011	down
ENSBTAG00000039462	*PCLAF*	protein-coding	-0,546202529	0,006170647	0,038663802	down
ENSBTAG00000008897	*BIN2*	protein-coding	-0,533158114	0,00618917	0,038762891	down
ENSBTAG00000030322	*NA*	NA	0,733767834	0,00622198	0,038900288	up

ENSBTAG00000051251	*NA*	NA	0,667903885	0,006271296	0,039097318	up
ENSBTAG00000047426	*LYPD6*	protein-coding	0,573174006	0,006274997	0,039097318	up
ENSBTAG00000004175	*HPD*	protein-coding	0,789750106	0,006382596	0,039627477	up
ENSBTAG00000047664	*GNAZ*	protein-coding	0,622114216	0,006404401	0,039692943	up
ENSBTAG00000002471	*CHL1*	protein-coding	-0,891787961	0,006440752	0,039867473	down
ENSBTAG00000007809	*PPP1R36*	protein-coding	0,539833841	0,0064816	0,040078978	up
ENSBTAG00000014912	*FMOD*	protein-coding	0,538432956	0,00655767	0,040354854	up
ENSBTAG00000026080	*LAIR1*	protein-coding	-0,73863141	0,006563518	0,040354854	down
ENSBTAG00000008092	*RAB37*	protein-coding	-0,57549231	0,006564608	0,040354854	down
ENSBTAG00000000328	*TPPP2*	protein-coding	0,74556738	0,006652029	0,040752218	up
ENSBTAG00000016704	*SLC37A2*	protein-coding	-0,577486233	0,006658362	0,040756124	down
ENSBTAG00000001562	*NFE2*	protein-coding	0,690115119	0,006725777	0,041072806	up
ENSBTAG00000003696	*BICDL1*	protein-coding	-0,506519185	0,006749889	0,041105386	down
ENSBTAG00000012834	*ARSI*	protein-coding	0,612431526	0,006870639	0,04166149	up
ENSBTAG00000012837	*NA*	NA	0,539482393	0,006919125	0,041815709	up
ENSBTAG00000051798	*NA*	NA	-0,536625647	0,006977273	0,042004186	down
ENSBTAG00000005419	*AOC1*	protein-coding	-0,761589754	0,00699881	0,042031038	down
ENSBTAG00000013334	*NA*	NA	0,637532056	0,007016182	0,042117693	up
ENSBTAG00000044071	*HRH2*	protein-coding	0,80338848	0,007043256	0,042227076	up
ENSBTAG00000005628	*CD52*	protein-coding	-0,504397426	0,007087973	0,042353228	down
ENSBTAG00000053581	*SUCNR1*	protein-coding	-0,579276642	0,007101168	0,042371357	down
ENSBTAG00000008380	*ITGA11*	protein-coding	0,506296005	0,007133682	0,042524743	up
ENSBTAG00000003920	*TGM1*	protein-coding	-0,555865157	0,007137477	0,042524743	down
ENSBTAG00000054461	*ITGAX*	protein-coding	-0,641102455	0,007161656	0,042609154	down
ENSBTAG00000017514	*CCND1*	protein-coding	0,555637266	0,007233724	0,042866178	up
ENSBTAG00000052033	*NA*	NA	0,526702498	0,007331116	0,043335644	up
ENSBTAG00000005244	*RASL11A*	protein-coding	-0,610041053	0,007782886	0,045121906	down
ENSBTAG00000002258	*APOA1*	protein-coding	-0,740284512	0,007818297	0,045261993	down
ENSBTAG00000001900	*NA*	NA	0,729867665	0,007896159	0,045620479	up
ENSBTAG00000030940	*GIMAP7*	protein-coding	-0,524571867	0,008212748	0,046900496	down
ENSBTAG00000035064	*C4H7orf57*	protein-coding	0,688670017	0,008274236	0,047176329	up
ENSBTAG00000049851	*PMAIP1*	protein-coding	-0,72609995	0,008328557	0,047448242	down
ENSBTAG00000044195	*SDK2*	protein-coding	0,549253876	0,008334445	0,047452047	up
ENSBTAG00000049393	*NA*	NA	-0,899096782	0,008347868	0,047483594	down
ENSBTAG00000004952	*MFSD4A*	protein-coding	0,585851782	0,008357988	0,047483594	up
ENSBTAG00000052931	*NA*	NA	-0,944550442	0,008536006	0,048207885	down
ENSBTAG00000007932	*HCK*	protein-coding	-0,564136747	0,008556521	0,048285637	down
ENSBTAG00000006515	*ESPN*	protein-coding	-0,545726794	0,008586238	0,048377027	down
ENSBTAG00000013160	*GFRA4*	protein-coding	-0,641099662	0,008667275	0,048642106	down
ENSBTAG00000050371	*NA*	NA	0,563619283	0,008734272	0,048875178	up

NA: Not Annoted

Table S3. Enriched KEGG pathways for up-regulated genes in group 2 at weaning

Category	Term	Count	P value	Genes
KEGG_PATHWAY	bta05410:Hypertrophic cardiomyopathy	11	3.85E-5	*CACNG7, PRKAB2, PRKAA2, TPM3, MYL2, MYL3, ITGA11, DAG1, CACNA2D2, TNNI3, MYH7*
KEGG_PATHWAY	bta04261:Adrenergic signaling in cardiomyocytes	13	1.37E-5	*TPM3, CAMK2A, ATP1A4, CACNA2D2, ATP2B2, PRKCA, CACNG7, MYL2, MYL3, TNNI3, CALM3, SCN4B, MYH7*
KEGG_PATHWAY	bta05200:Pathways in cancer	27	2.01E-5	*FLT4, LAMA3, CAMK2A, CXCR4, CBL, RASGRP1, RASGRP3, DLL4, FGF6, MECOM, CCND1, GNA12, E2F3, RXRG, NQO1, FZD2, SMAD3, TPM3, PRKCA, ESR1, AR, HEYL, COL4A2, CCNE1, KIT, BIRC5, CALM3*
KEGG_PATHWAY	bta03320:PPAR signaling pathway	9	5.75E-4	*FADS2, FABP3, SLC27A1, SCD, SCD5, AQP7, DBI, PPARA, RXRG*
KEGG_PATHWAY	bta04512:ECM-receptor interaction	9	8.47E-4	*COL1A1, COL4A2, LAMA3, ITGA11, DAG1, NPNT, AGRN, FREM1, FREM2*
KEGG_PATHWAY	bta00100:Steroid biosynthesis	5	9.08E-4	*SQLE, EBP, CYP51A1, DHCR24, LSS*
KEGG_PATHWAY	bta04260:Cardiac muscle contraction	9	0.00113	*CACNG7, TPM3, MYL2, MYL3, ATP1A4, CASQ2, CACNA2D2, TNNI3, MYH7*
KEGG_PATHWAY	bta05414:Dilated cardiomyopathy	9	0.00169	*CACNG7, TPM3, MYL2, MYL3, ITGA11, DAG1, CACNA2D2, TNNI3, MYH7*
KEGG_PATHWAY	bta04971:Gastric acid secretion	7	0.00712	*CA2, HRH2, CAMK2A, ATP1A4, CALM3, PRKCA, KCNJ2*
KEGG_PATHWAY	bta04921:Oxytocin signaling pathway	10	0.00758	*CACNG7, PRKAB2, PRKAA2, CCND1, CAMK2A, CACNA2D2, CALM3, PRKCA, MYL6B, KCNJ2*
KEGG_PATHWAY	bta04964:Proximal tubule bicarbonate reclamation	4	0.01260	*CA2, ATP1A4, SLC38A3, AQP1*
KEGG_PATHWAY	bta04360:Axon guidance	10	0.01964	*ENAH, EFNB2, SEMA6B, SEMA6C, UNC5A, CAMK2A, PAK6, PLXNA1, CXCR4, PRKCA*
KEGG_PATHWAY	bta05224:Breast cancer	9	0.01983	*DLL4, FGF6, HEYL, FZD2, CCND1, FLT4, KIT, E2F3, ESR1*
KEGG_PATHWAY	bta04020:Calcium signaling pathway	12	0.02686	*FGF6, HRH2, FLT4, CAMK2A, CASQ2, PPIF, CXCR4, ATP2B2, CALM3, PRKCA, CASQ1, TPCN1*
KEGG_PATHWAY	bta01040:Biosynthesis of unsaturated fatty acids	4	0.02915	*FADS2, SCD, SCD5, FADS1*
KEGG_PATHWAY	bta04010:MAPK signaling pathway	13	0.03196	*ANGPT1, SRF, FLT4, CACNA2D2, PRKCA, RASGRP1, RASGRP3, NTF4, CACNG7, FGF6, MECOM, KIT, GNA12*
KEGG_PATHWAY	bta04510:Focal adhesion	10	0.03761	*COL1A1, CCND1, COL4A2, MYL2, FLT4, LAMA3, ITGA11, PAK6, PRKCA, PARVB*
KEGG_PATHWAY	bta04371:Apelin signaling pathway	8	0.03974	*PRKAB2, SMAD3, PRKAA2, CCND1, MYL2, MYL3, APLNR, CALM3*
KEGG_PATHWAY	bta01212:Fatty acid metabolism	5	0.04109	*FADS2, SCD, FASN, SCD5, FADS1*
KEGG_PATHWAY	bta00760:Nicotinate and nicotinamide metabolism	4	0.04652	*NNT, NMRK2, NT5C1A, QPRT*
KEGG_PATHWAY	bta04014:Ras signaling pathway	11	0.04773	*NTF4, FGF6, ANGPT1, FLT4, KIT, REL, PAK6, CALM3, PRKCA, RASGRP1, RASGRP3*

Table S4. Enriched biological processes (GO Terms) for up-regulated genes in group 2 at weaning

Category	Term	Count	P value	Genes
GOTERM_BP_DIRECT	GO:0006695~cholesterol biosynthetic process	5	0,00231	*EBP, INSIG1, CYP51A1, DHCR24, LSS*
GOTERM_BP_DIRECT	GO:0045214~sarcomere organization	5	0,00265	*TNNT1, SRF, CASQ1, MYOZ2, MYPN*
GOTERM_BP_DIRECT	GO:0006636~unsaturated fatty acid biosynthetic process	4	0,00349	*FADS2, SCD, SCD5, FADS1*
GOTERM_BP_DIRECT	GO:0055010~ventricular cardiac muscle tissue morphogenesis	4	0,00506	*MYL2, MYL3, PROX1, MYH7*
GOTERM_BP_DIRECT	GO:0030240~skeletal muscle thin filament assembly	3	0,00627	*ACTA1, PROX1, LMOD3*
GOTERM_BP_DIRECT	GO:0043069~negative regulation of programmed cell death	3	0,00866	*TIGAR, MECOM, KIT*
GOTERM_BP_DIRECT	GO:0007507~heart development	8	0,01043	*ACVR1, PDLIM1, POPDC2, GJA1, MYL2, APLNR, SOX4, FREM2*
GOTERM_BP_DIRECT	GO:0010628~positive regulation of gene expression	14	0,01052	*RARG, SMAD3, ANGPT1, SCX, DLL4, AR, FGF6, ACTA1, GJA1, CUX2, DNAJA4, SOX8, ADTRP, LDLR*
GOTERM_BP_DIRECT	GO:0006936~muscle contraction	5	0,01153	*MYH2, MYBPC1, TNNT1, TPM3, LMOD3*
GOTERM_BP_DIRECT	GO:0001947~heart looping	5	0,01817	*GJA1, SMAD3, SRF, APLNR, MIB1*
GOTERM_BP_DIRECT	GO:0016126~sterol biosynthetic process	3	0,02145	*SQLE, EBP, INSIG1*
GOTERM_BP_DIRECT	GO:0043951~negative regulation of cAMP-mediated signaling	3	0,02539	*RNF157, PDE11A, APLNR*
GOTERM_BP_DIRECT	GO:0007512~adult heart development	3	0,02539	*GJA1, APLNR, MYH7*
GOTERM_BP_DIRECT	GO:0061351~neural precursor cell proliferation	3	0,02539	*BBS1, NDUFS2, DBN1*
GOTERM_BP_DIRECT	GO:0002026~regulation of the force of heart contraction	3	0,02539	*MYL2, MYL3, MYH7*
GOTERM_BP_DIRECT	GO:0007219~Notch signaling pathway	6	0,03078	*DLL4, HEYL, GOT1, NRARP, GRIP2, MIB1*
GOTERM_BP_DIRECT	GO:0022008~neurogenesis	4	0,03170	*NAV3, NDUFS2, CXCR4, DCHS1*
GOTERM_BP_DIRECT	GO:0032869~cellular response to insulin stimulus	5	0,03188	*GOT1, INSIG1, INHBB, LPIN2, HDAC9*
GOTERM_BP_DIRECT	GO:0001894~tissue homeostasis	3	0,03406	*TP53INP2, COL11A2, SCX*
GOTERM_BP_DIRECT	GO:0007154~cell communication	3	0,03406	*GJA1, FREM1, FREM2*
GOTERM_BP_DIRECT	GO:0060749~mammary gland alveolus development	3	0,03406	*AR, CCND1, ESR1*
GOTERM_BP_DIRECT	GO:0001701~in utero embryonic development	9	0,03408	*ACVR1, AR, GJA1, SMAD3, GNA12, SOX8, DBN1, TANC2, MIB1*
GOTERM_BP_DIRECT	GO:0001568~blood vessel development	4	0,03424	*COL1A1, BMPER, APLNR, MIB1*
GOTERM_BP_DIRECT	GO:0001649~osteoblast differentiation	5	0,03557	*COL1A1, GJA1, IGFBP5, TP53INP2, SOX8*
GOTERM_BP_DIRECT	GO:0006730~one-carbon metabolic process	4	0,03689	*ALDH1L1, CA3, CA2, CA14*
GOTERM_BP_DIRECT	GO:0000082~G1/S transition of mitotic cell cycle	4	0,03963	*CCND1, CCNE1, E2F3, CACUL1*

GOTERM_BP_DIRECT	GO:0007188~adenylate cyclase-modulating G-protein coupled receptor signaling pathway	4	0,03963	*GNAZ, GNAT2, GNA12, PTH2R*
GOTERM_BP_DIRECT	GO:0090263~positive regulation of canonical Wnt signaling pathway	6	0,04145	*COL1A1, FAM53B, NRARP, LYPD6, DKK2, SOX4*
GOTERM_BP_DIRECT	GO:0002042~cell migration involved in sprouting angiogenesis	3	0,04369	*EFNB2, SRF, ADTRP*
GOTERM_BP_DIRECT	GO:0002009~morphogenesis of an epithelium	3	0,04369	*DAG1, SOX8, FREM2*
GOTERM_BP_DIRECT	GO:0002027~regulation of heart rate	3	0,04369	*CASQ2, CALM3, MYH7*
GOTERM_BP_DIRECT	GO:0035914~skeletal muscle cell differentiation	4	0,04542	*HEYL, SCX, SOX8, HIVEP3*
GOTERM_BP_DIRECT	GO:0051965~positive regulation of synapse assembly	4	0,04846	*CUX2, CLSTN2, AMIGO2, LRRN1*
GOTERM_BP_DIRECT	GO:0003009~skeletal muscle contraction	3	0,04884	*MYH3, TNNT1, MYH7*

Table S5. Enriched KEGG pathways for down-regulated genes in group 2 at weaning

Category	Term	Count	P value	Genes
KEGG_PATHWAY	bta04610:Complement and coagulation cascades	17	2.84E-10	*CFD, C1QB, C1QA, C5AR1, ITGB2, SERPINE1, PLAUR, F13A1, F3, C2, C3, C7, ITGAX, C3AR1, VSIG4, CFB, C1QC*
KEGG_PATHWAY	bta05150:Staphylococcus aureus infection	18	3.30E-10	*CFD, C1QB, C1QA, C5AR1, PTAFR, ITGB2, BOLA-DQA2, ITGAL, C2, C3, FCGR3A, FCGR2A, LOC100300510, C3AR1, FCGR1A, BOLA-DMB, CFB, C1QC*
KEGG_PATHWAY	bta05202:Transcriptional misregulation in cancer	20	1.68E-7	*CD86, CSF1R, CEBPB, CDKN1A, SPI1, MAX, GADD45A, FLT3, ZBTB16, FOXO1, GADD45G, RUNX1, HOXA9, LOC100300510, MYC, IL2RB, NUPR1, CD14, PROM1, FCGR1A*
KEGG_PATHWAY	bta04936:Alcoholic liver disease	17	4.61E-7	*C1QB, C1QA, CPT1A, C5AR1, CXCL3, CPT1B, MAPK12, FOXO1, C2, C3, NFKBIA, C3AR1, MLYCD, CD14, LPIN1, IKBKE, C1QC*
KEGG_PATHWAY	bta05140:Leishmaniasis	11	1.09E-5	*C3, NFKBIA, FCGR3A, FCGR2A, NCF2, ITGB2, BOLA-DQA2, PTGS2, FCGR1A, BOLA-DMB, MAPK12*
KEGG_PATHWAY	bta04659:Th17 cell differentiation	12	7.09E-5	*NFKBIA, LOC618409, LOC100300510, LCK, IL2RB, IL21R, BOLA-DQA2, IL1RAP, BOLA-DMB, LAT, MAPK12, RUNX1*
KEGG_PATHWAY	bta05221:Acute myeloid leukemia	9	1.57E-4	*CSF1R, SPI1, FLT3, MYC, ZBTB16, EIF4EBP1, CD14, FCGR1A, RUNX1*
KEGG_PATHWAY	bta05152:Tuberculosis	15	2.70E-4	*CEBPB, FCER1G, IL10RA, ITGB2, BOLA-DQA2, MAPK12, C3, FCGR3A, FCGR2A, MRC1, ITGAX, BCL2, CD14, FCGR1A, BOLA-DMB*
KEGG_PATHWAY	bta04380:Osteoclast differentiation	12	3.34E-4	*NFKBIA, SOCS3, CSF1R, FCGR3A, FCGR2A, SPI1, NCF2, LCK, LCP2, FCGR1A, MAPK12, FOSL2*
KEGG_PATHWAY	bta05133:Pertussis	9	4.09E-4	*C3, C1QB, C1QA, ITGB2, IRF8, CD14, MAPK12, C1QC, C2*
KEGG_PATHWAY	bta04064:NF-kappa B signaling pathway	10	0,00109	*NFKBIA, GADD45A, LOC100300510, LCK, BCL2, CD14, PTGS2, CXCL3, LAT, GADD45G*
KEGG_PATHWAY	bta05222:Small cell lung cancer	9	0,00142	*NFKBIA, CDKN1A, GADD45A, MAX, MYC, BCL2, PTGS2, FHIT, GADD45G*
KEGG_PATHWAY	bta05142:Chagas disease	10	0,00199	*C3, C1QB, NFKBIA, C1QA, GNA15, LOC100300510, SERPINE1, PLCB2, MAPK12, C1QC*
KEGG_PATHWAY	bta04145:Phagosome	12	0,00207	*C3, MSR1, FCGR3A, FCGR2A, NCF2, ITGB2, MRC1, BOLA-DQA2, CD14, FCGR1A, RILP, BOLA-DMB*
KEGG_PATHWAY	bta05171:Coronavirus disease - COVID-19	16	0,00212	*CFD, C1QB, C1QA, RPL12, C5AR1, F13A1, MAPK12, C2, C3, NFKBIA, CXCL10, C7, C3AR1, IKBKE, CFB, C1QC*
KEGG_PATHWAY	bta05216:Thyroid cancer	6	0,00212	*RET, CDKN1A, GADD45A, CDH1, MYC, GADD45G*
KEGG_PATHWAY	bta04115:p53 signaling pathway	8	0,00239	*CDKN1A, GADD45A, SESN1, SERPINE1, BCL2, PMAIP1, TP73, GADD45G*
KEGG_PATHWAY	bta05200:Pathways in cancer	24	0,00337	*ALK, RET, CSF1R, GSTM3, CDKN1A, SPI1, MAX, GADD45A, FLT3, ZBTB16, PTGER3, PTGS2, AGT, FOXO1, GADD45G, RUNX1, NFKBIA, CDH1, MYC, IL2RB, BCL2, HMOX1, PMAIP1, PLCB2*
KEGG_PATHWAY	bta04640:Hematopoietic cell lineage	9	0,00382	*CD2, CSF1R, CD8A, FLT3, CD7, BOLA-DQA2, CD14, FCGR1A, BOLA-DMB*
KEGG_PATHWAY	bta04621:NOD-like receptor signaling pathway	12	0,00535	*GSDMD, LOC781710, NFKBIA, GBP5, PSTPIP1, NAMPT, BCL2, TXNIP, CXCL3, IKBKE, PLCB2, MAPK12*
KEGG_PATHWAY	bta04061:Viral protein interaction with cytokine and cytokine receptor	8	0,00623	*CXCL10, CSF1R, CXCL9, CCL22, IL10RA, IL2RB, CXCL3, IL18R1*

KEGG_PATHWAY	bta05322:Systemic lupus erythematosus	12	0,00728	*C3, C1QB, CD86, C1QA, FCGR3A, C7, LOC100300510, BOLA-DQA2, FCGR1A, BOLA-DMB, C1QC, C2*
KEGG_PATHWAY	bta04658:Th1 and Th2 cell differentiation	8	0,00778	*NFKBIA, LOC100300510, LCK, IL2RB, BOLA-DQA2, BOLA-DMB, LAT, MAPK12*
KEGG_PATHWAY	bta05169:Epstein-Barr virus infection	13	0,00813	*CDKN1A, GADD45A, BOLA-DQA2, ITGAL, MAPK12, GADD45G, NFKBIA, CXCL10, LOC100300510, MYC, BCL2, IKBKE, BOLA-DMB*
KEGG_PATHWAY	bta04514:Cell adhesion molecules	10	0,01304	*CD2, SPN, CD86, CD8A, LOC100300510, CDH1, ITGB2, BOLA-DQA2, ITGAL, BOLA-DMB*
KEGG_PATHWAY	bta05235:PD-L1 expression and PD-1 checkpoint pathway in cancer	7	0,02123	*NFKBIA, ALK, BATF3, LOC100300510, LCK, LAT, MAPK12*
KEGG_PATHWAY	bta04657:IL-17 signaling pathway	7	0,02225	*NFKBIA, CXCL10, CEBPB, PTGS2, CXCL3, IKBKE, MAPK12*
KEGG_PATHWAY	bta04152:AMPK signaling pathway	8	0,02346	*PFKFB4, CPT1A, PFKFB3, EIF4EBP1, MLYCD, CPT1B, FBP1, FOXO1*
KEGG_PATHWAY	bta04668:TNF signaling pathway	8	0,02439	*NFKBIA, SOCS3, CXCL10, CEBPB, PTGS2, CXCL3, IL18R1, MAPK12*
KEGG_PATHWAY	bta00330:Arginine and proline metabolism	5	0,02592	*AOC1, P4HA3, CKB, SAT1, AGMAT*
KEGG_PATHWAY	bta05223:Non-small cell lung cancer	6	0,02631	*RET, ALK, CDKN1A, GADD45A, FHIT, GADD45G*
KEGG_PATHWAY	bta04650:Natural killer cell mediated cytotoxicity	9	0,02869	*FCGR3A, LOC100336869, FCER1G, LCK, ITGB2, CD48, LCP2, ITGAL, LAT*
KEGG_PATHWAY	bta04015:Rap1 signaling pathway	11	0,02883	*APBB1IP, CSF1R, LOC100300510, CDH1, ITGB2, LCP2, ITGAL, PLCB2, LAT, MAPK12, RAPGEF4*
KEGG_PATHWAY	bta05167:Kaposi sarcoma-associated herpesvirus infection	11	0,03097	*C3, NFKBIA, CD86, HCK, CDKN1A, MYC, CD200R1L, PTGS2, CXCL3, IKBKE, MAPK12*
KEGG_PATHWAY	bta04933:AGE-RAGE signaling pathway in diabetic complications	7	0,03152	*SERPINE1, BCL2, F3, PLCB2, AGT, MAPK12, FOXO1*
KEGG_PATHWAY	bta04922:Glucagon signaling pathway	7	0,03152	*CPT1A, GCGR, SIK1, CPT1B, FBP1, PLCB2, FOXO1*
KEGG_PATHWAY	bta05220:Chronic myeloid leukemia	6	0,03217	*NFKBIA, CDKN1A, GADD45A, MYC, RUNX1, GADD45G*
KEGG_PATHWAY	bta05416:Viral myocarditis	6	0,03376	*CD86, LOC100300510, ITGB2, BOLA-DQA2, ITGAL, BOLA-DMB*
KEGG_PATHWAY	bta05323:Rheumatoid arthritis	7	0,03556	*CD86, LOC100300510, ITGB2, BOLA-DQA2, ITGAL, CXCL3, BOLA-DMB*
KEGG_PATHWAY	bta04218:Cellular senescence	9	0,03695	*CDKN1A, LIN37, GADD45A, MYC, SERPINE1, EIF4EBP1, MAPK12, FOXO1, GADD45G*
KEGG_PATHWAY	bta04660:T cell receptor signaling pathway	7	0,03993	*NFKBIA, CD8A, LOC100300510, LCK, LCP2, LAT, MAPK12*
KEGG_PATHWAY	bta03320:PPAR signaling pathway	6	0,04427	*CPT1A, APOA1, ME3, ANGPTL4, CPT1B, PLIN5*
KEGG_PATHWAY	bta05213:Endometrial cancer	5	0,04670	*CDKN1A, GADD45A, CDH1, MYC, GADD45G*
KEGG_PATHWAY	bta05134:Legionellosis	5	0,04670	*C3, NFKBIA, ITGB2, CD14, CXCL3*
KEGG_PATHWAY	bta05166:Human T-cell leukemia virus 1 infection	11	0,04858	*NFKBIA, CDKN1A, SPI1, LOC100300510, LCK, MYC, ITGB2, IL2RB, BOLA-DQA2, ITGAL, BOLA-DMB*
KEGG_PATHWAY	bta05417:Lipid and atherosclerosis	11	0,04976	*NFKBIA, ABCA1, NCF2, BCL2, APOA1, CD14, CXCL3, IKBKE, PLCB2, MAPK12, ABCG1*

Table S6. Enriched biological processes (GO Terms) for down-regulated genes in group 2 at weaning

Category	Term	Count	P value	Genes
GOTERM_BP_DIRECT	GO:0006954~inflammatory response	17	3.12E-5	*CSF1R, CXCL9, CCL22, LOC504773, C5AR1, PTAFR, PTGER3, PTGS2, CXCL3, AIF1, C3, CXCL10, C3AR1, CHI3L1, CD14, LAT, IDO1*
GOTERM_BP_DIRECT	GO:0030593~neutrophil chemotaxis	9	4.39E-5	*CXCL10, FCER1G, CCL22, ITGB2, LOC504773, C5AR1, PDE4B, NCKAP1L, CXCL3*
GOTERM_BP_DIRECT	GO:0009617~response to bacterium	9	1.76E-4	*C3, CFD, CXCL9, BAIAP2L1, RGS1, SCN7A, TENT5A, CD14, FKBP5*
GOTERM_BP_DIRECT	GO:0071222~cellular response to lipopolysaccharide	9	7.29E-4	*CD86, CXCL10, TNIP3, MRC1, ARID5A, PDE4B, IRF8, CD14, CXCL3*
GOTERM_BP_DIRECT	GO:0010884~positive regulation of lipid storage	4	7.37E-4	*C3, IKBKE, FAM71F2, PLIN5*
GOTERM_BP_DIRECT	GO:0090501~RNA phosphodiester bond hydrolysis	4	7.37E-4	*BRB, RNASE6, RNASE1*
GOTERM_BP_DIRECT	GO:0045087~innate immune response	18	9.98E-4	*C1QB, BTNL9, C1QA, FCER1G, RIOK3, RNASE6, ARID5A, SLA, MPEG1, C2, HCK, C7, LOC100300510, LCK, TIFA, LOC515676, CD14, TMEM106A*
GOTERM_BP_DIRECT	GO:0007169~transmembrane receptor protein tyrosine kinase signaling pathway	9	0,00111	*RET, ALK, HCK, CSF1R, FLT3, LCK, CASS4, SLA, LCP2*
GOTERM_BP_DIRECT	GO:0071346~cellular response to interferon-gamma	8	0,00118	*LOC781710, GBP5, CCL22, LOC504773, MRC1, WAS, IRF8, AIF1*
GOTERM_BP_DIRECT	GO:0006957~complement activation, alternative pathway	4	0,00140	*C3, CFD, C7, CFB*
GOTERM_BP_DIRECT	GO:0098586~cellular response to virus	5	0,00182	*CXCL10, RIOK3, WDFY4, IKBKE, GSDME*
GOTERM_BP_DIRECT	GO:0006958~complement activation, classical pathway	6	0,00237	*C3, C1QB, C1QA, C7, LOC100300510, C2*
GOTERM_BP_DIRECT	GO:0072593~reactive oxygen species metabolic process	5	0,00279	*SESN1, DDIT4, PDK4, BCL2, CCN1*
GOTERM_BP_DIRECT	GO:0051017~actin filament bundle assembly	5	0,00319	*BAIAP2L2, BAIAP2L1, LCP1, ESPN, AIF1*
GOTERM_BP_DIRECT	GO:0042742~defense response to bacterium	10	0,00381	*GSDMD, SPN, CEBPB, FCER1G, LOC100300510, GNLY, LOC104968634, IRF8, LYZ, MPEG1*
GOTERM_BP_DIRECT	GO:0097242~beta-amyloid clearance	3	0,00436	*C3, MSR1, C5AR1*
GOTERM_BP_DIRECT	GO:0006631~fatty acid metabolic process	6	0,00536	*C3, CPT1A, ACOT7, UCP3, HACL1, CPT1B*
GOTERM_BP_DIRECT	GO:0007229~integrin-mediated signaling pathway	7	0,00568	*ITGB2, ITGAX, APOA1, ITGAL, PRAM1, LAT, FERMT3*
GOTERM_BP_DIRECT	GO:0032729~positive regulation of interferon-gamma production	6	0,00628	*CD2, ARID5A, PDE4B, IRF8, CD14, IL18R1*
GOTERM_BP_DIRECT	GO:0042832~defense response to protozoan	4	0,00966	*LOC781710, CCDC88B, IRF8, NKG7*
GOTERM_BP_DIRECT	GO:0034612~response to tumor necrosis factor	4	0,00966	*UBD, CHI3L1, LOC504548, CXCL16*
GOTERM_BP_DIRECT	GO:1990542~mitochondrial transmembrane transport	3	0,01170	*UCP3, UCP2, SFXN1*
GOTERM_BP_DIRECT	GO:0006898~receptor-mediated endocytosis	5	0,01210	*MSR1, MRC1, CD14, SCARF1, CXCL16*
GOTERM_BP_DIRECT	GO:0048008~platelet-derived growth factor receptor signaling pathway	4	0,01403	*CSRNP1, ZFAND5, TXNIP, ARID5B*
GOTERM_BP_DIRECT	GO:0006000~fructose metabolic process	3	0,01483	*PFKFB4, PFKFB3, FBP1*
GOTERM_BP_DIRECT	GO:0006935~chemotaxis	5	0,01651	*CXCL10, CXCL9, PTGDR2, PTAFR, C3AR1*
GOTERM_BP_DIRECT	GO:0045766~positive regulation of angiogenesis	7	0,01693	*C3, BTG1, C5AR1, C3AR1, CHI3L1, HMOX1, F3*
GOTERM_BP_DIRECT	GO:0034341~response to interferon-gamma	4	0,01747	*CITED1, UBD, LOC504548, CXCL16*

GOTERM_BP_DIRECT	GO:0050776~regulation of immune response	4	0,01747	*SPN, FCGR3A, FCGR2A, FCGR1A*
GOTERM_BP_DIRECT	GO:0016322~neuron remodeling	3	0,01828	*C3, C1QA, SCARF1*
GOTERM_BP_DIRECT	GO:0006909~phagocytosis	5	0,02040	*NCF2, ITGB2, IRF8, PLD4, ITGAL*
GOTERM_BP_DIRECT	GO:0050853~B cell receptor signaling pathway	5	0,02181	*LAT2, LOC100300510, LCK, BCL2, NCKAP1L*
GOTERM_BP_DIRECT	GO:0007155~cell adhesion	13	0,02190	*POSTN, TNC, ITGAL, PARVG, GPNMB, LOC112442215, CHL1, CASS4, ITGAX, LPXN, CCN1, FOLR2, FERMT3*
GOTERM_BP_DIRECT	GO:0051764~actin crosslink formation	3	0,02204	*BAIAP2L2, BAIAP2L1, AIF1*
GOTERM_BP_DIRECT	GO:0045820~negative regulation of glycolytic process	3	0,02204	*DDIT4, NUPR1, FBP1*
GOTERM_BP_DIRECT	GO:0031663~lipopolysaccharide-mediated signaling pathway	4	0,02793	*NFKBIA, SPI1, PTAFR, CD14*
GOTERM_BP_DIRECT	GO:0006919~activation of cysteine-type endopeptidase activity involved in apoptotic process	5	0,02804	*LCK, MYC, PMAIP1, XDH, F3*
GOTERM_BP_DIRECT	GO:0050729~positive regulation of inflammatory response	5	0,02804	*NFKBIA, IL1RL1, CEBPB, SUCNR1, NKG7*
GOTERM_BP_DIRECT	GO:0050919~negative chemotaxis	4	0,03287	*SEMA4A, RTN4RL1, SEMA3B, APOA1*
GOTERM_BP_DIRECT	GO:0071356~cellular response to tumor necrosis factor	6	0,03391	*LOC781710, CCL22, LOC504773, CHI3L1, YBX3, GSDME*
GOTERM_BP_DIRECT	GO:0034142~toll-like receptor 4 signaling pathway	3	0,03496	*NFKBIA, TNIP3, CD14*
GOTERM_BP_DIRECT	GO:0010759~positive regulation of macrophage chemotaxis	3	0,03496	*CSF1R, C5AR1, C3AR1*
GOTERM_BP_DIRECT	GO:0007166~cell surface receptor signaling pathway	8	0,03741	*SPN, CD86, FCGR3A, FCGR2A, CD8A, ADGRG5, GCGR, FCGR1A*
GOTERM_BP_DIRECT	GO:0007159~leukocyte cell-cell adhesion	3	0,03978	*ITGB2, ITGAL, FERMT3*
GOTERM_BP_DIRECT	GO:0090023~positive regulation of neutrophil chemotaxis	3	0,03978	*C5AR1, C3AR1, NCKAP1L*
GOTERM_BP_DIRECT	GO:0042493~response to drug	5	0,04126	*LCK, MYC, BCL2, APOD, NCKAP1L*
GOTERM_BP_DIRECT	GO:0050830~defense response to Gram-positive bacterium	6	0,04189	*GSDMD, LOC781710, C5AR1, RNASE6, LYZ, MPEG1*
GOTERM_BP_DIRECT	GO:0006915~apoptotic process	11	0,04281	*TNFAIP8, CITED1, TCIM, DDIT4, BCL2, CHI3L1, HMOX1, DNASE1L3, BCL2L14, FOXO1, GADD45G*
GOTERM_BP_DIRECT	GO:0006911~phagocytosis, engulfment	4	0,04400	*MSR1, BIN2, LOC100300510, AIF1*
GOTERM_BP_DIRECT	GO:2000379~positive regulation of reactive oxygen species metabolic process	3	0,04483	*CDKN1A, GADD45A, XDH*
GOTERM_BP_DIRECT	GO:0030335~positive regulation of cell migration	8	0,04621	*RET, SH3RF2, SEMA4A, PDPN, SEMA3B, CCN1, CXCL16, FERMT3*
GOTERM_BP_DIRECT	GO:0043065~positive regulation of apoptotic process	9	0,04905	*KLF11, TNFAIP8, GADD45A, ZBTB16, UBD, HMOX1, CCN1, LOC504548, FOXO1*

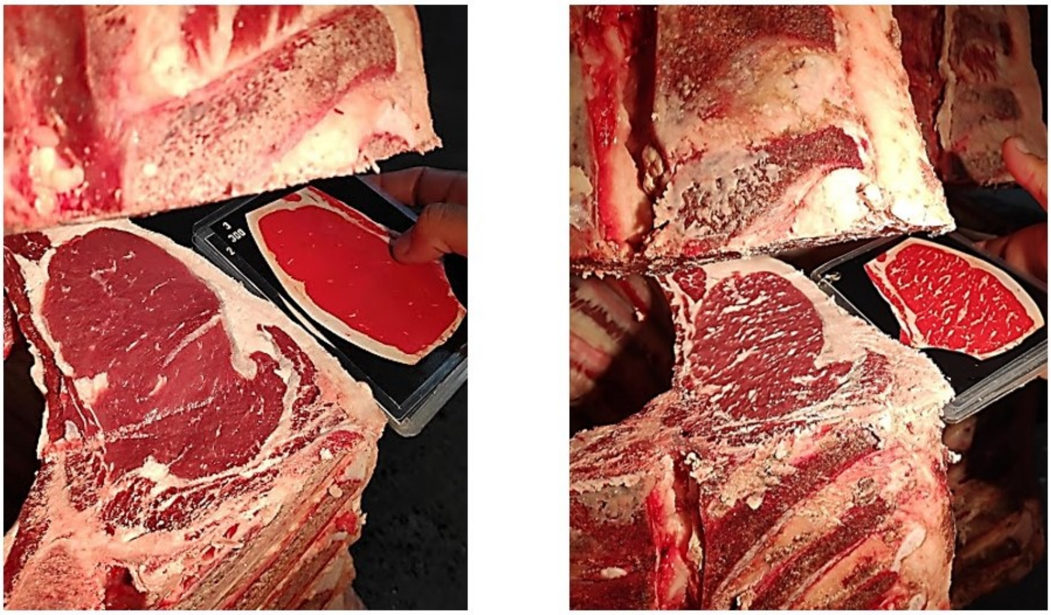

G1: control **G2: creep feeding**

Figure S1. Visual differences in relation to the fat content between *Longissimus thoracis* muscle samples collected from animals of G1 and G2.

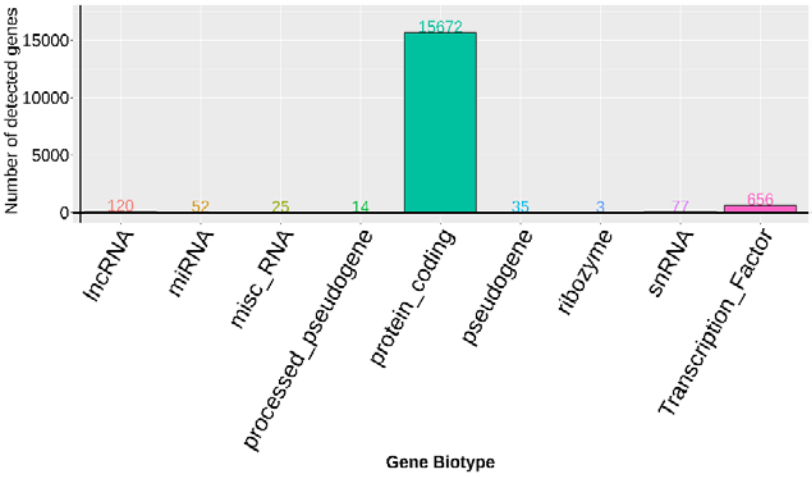

Figure S2. Number of genes by functional category detected in RNA sequencing considering the application of a filter that excludes genes with a low relative count for read count after normalization.

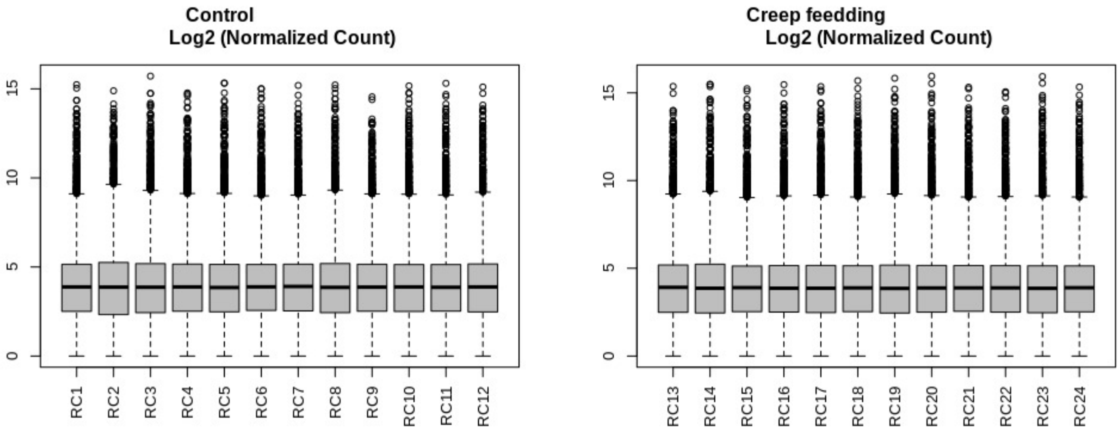

Figure S3. Log2 boxplot of the read count normalized by the size factor per sample in G1 (control, no creep feeding) and G2 (creep feeding).
